# An efficient and targeted synthetic approach towards new highly substituted 6-amino-pyrazolo[1,5-*a*]pyrimidines with *α*-glucosidase inhibitory activity

**DOI:** 10.1038/s41598-020-59079-z

**Published:** 2020-02-13

**Authors:** Fariba Peytam, Mehdi Adib, Reihaneh Shourgeshty, Loghman Firoozpour, Mahmoud Rahmanian-Jazi, Mehdi Jahani, Setareh Moghimi, Kouros Divsalar, Mohammad Ali Faramarzi, Somayeh Mojtabavi, Fatemeh Safari, Mohammad Mahdavi, Alireza Foroumadi

**Affiliations:** 1 School of Chemistry, College of Science, University of Tehran, Tehran, Iran; 2 Department of Medicinal Chemistry, Faculty of Pharmacy and The Institute of Pharmaceutical Sciences (TIPS), Tehran University of Medical Sciences, Tehran, Iran; 3 Neuroscience Research Center, Institute of Neuropharmacology, Kerman University of Medical Sciences, Kerman, Iran; 4 Department of Pharmaceutical Biotechnology, Faculty of Pharmacy, Tehran University of Medical Sciences, Tehran, Iran; 5 Department of Biology, Faculty of Science, University of Guilan, Rasht, Iran; 6 Endocrinology and Metabolism Research Center, Endocrinology and Metabolism Clinical Sciences Institute, Tehran University of Medical Sciences, Tehran, Iran

**Keywords:** Enzymes, Small molecules

## Abstract

In an attempt to find novel *α*-glucosidase inhibitors, an efficient, straightforward reaction to synthesize a library of fully substituted 6-amino-pyrazolo[1,5-*a*]pyrimidines **3** has been investigated. Heating a mixture of α-azidochalcones **1** and 3-aminopyrazoles **2** under the mild condition afforded desired compounds with a large substrate scope in good to excellent yields. All obtained products were evaluated as *α*-glucosidase inhibitors and exhibited excellent potency with IC_50_ values ranging from 15.2 ± 0.4 µM to 201.3 ± 4.2 µM. Among them, compound **3d** was around 50-fold more potent than acarbose (IC_50_ = 750.0 ± 1.5 µM) as standard inhibitor. Regarding product structures, kinetic study and molecular docking were carried out for two of the most potent ones.

## Introduction

Recently, the world health organization (WHO) has identified the diabetes mellitus (DM) as a critical health challenge in the 21st century. The prevalence of diabetes has been increasing at alarming rate over the past three decades. This metabolic disorder, characterized by chronic hyperglycemia, leads to further severe damages like abnormally great thrust, excessive appetite, overweigh, blindness, excessive urination, cardiovascular complications, as well as renal and neurodegenerative diseases^1–6^. There are three main diabetes types among which type 2 or non-insulin dependent (T2DM) is the most common one, mainly treated by controlling the digestive enzyme activities such as *α*-glucosidase^7–9^.

*α*-Glucosidase, found in the brush-border surface membrane of intestinal cells, plays catalyzing role in the carbohydrate digestion process by which the postprandial blood glucose levels increases. Preventing the glucose release in the bloodstream, the *α*-glucosidase inhibitors control T2DM^10^. Additionally, this enzyme has a pivotal role in the biosynthesis of glycoprotein, therefore, its inhibitors have possessed anticancer, antitumor, antiviral, and immunoregulatory properties^11–15^. Acrabose, miglitol, voglibose, and deoxynojirimycin have clinically been used to restrict the *α*-glucosidase activity^16^. Considering the side effects and absorption problems associated with these drugs, new scaffolds should be synthesized and evaluated by medicinal chemists to extend the library of compounds^17–25^.

**Figure 1:**
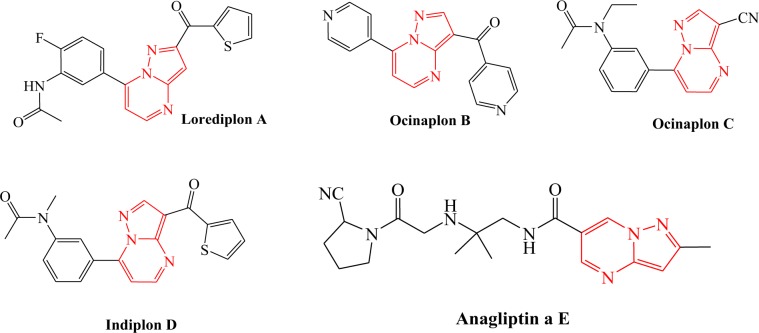
Example of marketed drugs with pyrazolo[1,5-*a*]pyrimidine core.

Pyrazoles are common structural motif in numerous drugs^26^ and biologically active compounds showing activities such as anti-cancer^27,28^, anti-inflammatory^29^, anti-hypertensive^30^, cannabinoid receptor antagonist^31^, dopaminergic receptor antagonist^32^, and *α*-glucosidase inhibitors^33^. Pyrazoles have been used to synthesize several other fused heterocycles. Pyrazolo[1,5-*a*]pyrimidines, in particular, have exhibited valuable pharmaceutical applications including various kinase inhibitors^34–37^, COX-2 inhibitors^38^, anti-viral (hepatetitis C and HIV)^39,40^, antimicrobial^41–43^, anxiolytic^44,45^, as well as positron emission tomography (PET) tumor imaging agents^46^. Some compounds containing this scaffold are approved and commercialized drugs, for example: Lorediplon **A** (for insomnia), Ocinaplon **B** (for anxiety), Zaleplon **C** and Indiplon **D** (for sedative and hypnotics), as well as Anagliptin **E** (for type 2 diabetes mellitus) Fig. 1)^47^. Furthermore, some pyrazolo[1,5-*a*]pyrimidines can be used in the treatment of diabetes^48^, obesity^49^, and CNS diseases^50^.

So far, different synthetic routes towards pyrazolo[1,5-*a*]pyrimidines have been reported. These methods have been mainly included the condensation of 3-aminopyrazoles with 1,3-bis electrophilic substrates^51–59^, 1,2-allenic lactones^60^, *β*-halovinyl aldehyde^61^, and activated alkynes^62,63^.

Through literature review, the reaction of *α*-azidochalcones **1** with 3-aminopyrazoles **2** to produce this scaffold has not proposed yet. Considering the significant mentioned roles in drug discovery process, synthesis of new derivatives of this scaffold is of increasing importance in medicinal organic chemistry. Therefore, we herein carried out an efficient, facile method to obtain highly substituted 6-amino-pyrazolo[1,5-*a*]pyrimidines **3** which have been studied to inhibit *α*-glucosidase.

## Results

### Chemistry

**Scheme 1:**
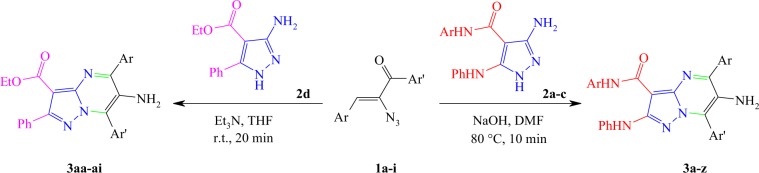
The general synthetic route for highly substituted 6-amino-pyrazolo[1,5-*a*]pyrimidines **3**.

In this paper, we described a targeted reaction for the synthesis of a series of novel poly functionalized 6-amino-pyrazolo[1,5-*a*]pyrimidines **3** by Michael-addition-cyclization of *α*-azidochalcones **1** with 3-aminopyrazoles **2** (Scheme 1). It should be also noted that *α*-azidochalcones **1** have been applied over the last decade to prepare several valuable nitrogen containing skeletons^64–75^. To probe the generality of this strategy, various derivatives of both starting materials were applied under the appropriate reaction condition to afford a large library of corresponding 6-amino-pyrazolo[1,5-*a*]pyrimidines **3** in 65–92% yields.

**Scheme 2:**
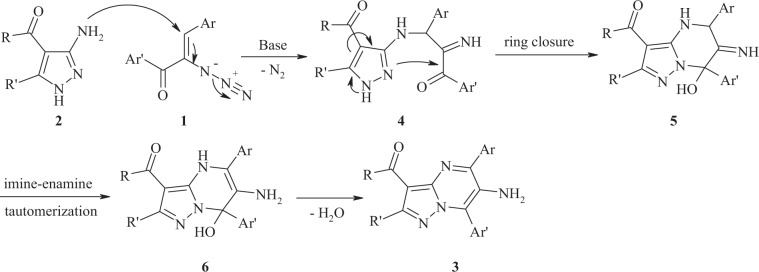
Proposed reaction mechanism.

A reasonable mechanism for this reaction is outlined in Scheme 2. Michael-addition of 3-aminopyrazole **2** from NH_2_ group to *α*-azidochalcones **1** and consequent removal of nitrogen molecule gives adduct **4**. Next, an intramolecular nucleophilic attack of NH group in the pyrazole moiety to adjacent carbonyl group takes place to form pyrazolo[1,5-*a*]pyrimidine skeleton (intermediate **5**). Finally, an imine-enamine tautomerization, followed by removal of a water molecule provides desired products **3**.

### *In vitro* α-glucosidase inhibitory activity

The obtained highly substituted 6-amino-pyrazolo[1,5-*a*]pyrimidines **3** were evaluated for their *in vitro* inhibitory activities against *α*-glucosidase (*Saccharomyces cerevisiae*, EC.3.2.1.20) and the results were compared with acarbose as the reference drug (Tables 1 and 2). As it can be seen, all the synthesized compounds showed good to excellent inhibitory activities with IC_50_ values of 15.2 ± 0.4−201.3 ± 4.2 µM in comparison to the standard drug IC_50_ = 750.0 ± 1.5 µM. To explain the structure and observed activity correlations, the 6-amino-pyrazolo[1,5-*a*]pyrimidines **3** were divided into two categories based on the substituents on the pyrazole moiety: the presence of amide functional group at C3-position **3a–z** (summarized in Table 1) along with ester functional group at C3-position **3aa-ai** (summarized in Table 2). Additionally, the substituents on the 5-phenyl and 7-phenyl rings of pyrimidine ring were changed in each series to optimize the *α*-glucosidase inhibition.

**Table 1:** Substrate scope and *in vitro* α-glucosidase inhibitory activity of compounds**3a–z**.


Compound	Ar	Ar'	Ar”	IC_50_ (µM)^a^
**3a**				37.8 ± 2.5
**3b**				40.0 ± 2.9
**3c**				44.5 ± 3.1
**3d**				15.2 ± 0.4
**3e**				63.3 ± 4.4
**3f**				85.7 ± 5.3
**3g**				75.6 ± 5.0
**3h**				25.7 ± 1.1
**3i**				28.1 ± 1.2
**3j**				30.3 ± 2.1
**3k**				57.4 ± 3.8
**3l**				49.3 ± 3.3
**3m**				24.7 ± 1.1
**3n**				42.6 ± 1.1
**3o**				56.2 ± 3.2
**3p**				33.2 ± 2.1
**3q**				36.5 ± 2.5
**3r**				18.4 ± 0.6
**3s**				42.3 ± 3.1
**3t**				60.0 ± 4.0
**3u**				55.6 ± 3.6
**3v**				17.6 ± 0.6
**3w**				53.7 ± 3.4
**3×**				80.3 ± 5.2
**3y**				38.2 ± 2.8
**3z**				19.3 ± 0.9
**Acarbose**	—	—	—	750.0 ± 1.5

^a^Values are the mean ± SD. All experiments were performed at least three times.

**Table 2:** Substrate scope and *in vitro* α-glucosidase inhibitory activity of compounds **3aa-ai**.


Compound	Ar	Ar'	IC_50_ (µM)^a^
**3aa**			94.0 ± 3.6
**3ab**			150.4 ± 4.0
**3ac**			185.0 ± 6.0
**3ad**			141.0 ± 7.0
**3ae**			116.3 ± 1.8
**3af**			65.5 ± 3.0
**3ag**			201.3 ± 4.2
**3ah**			161.7 ± 3.2
**3ai**			153.0 ± 5.0
**Acarbose**	—	—	750.0 ± 1.5

^a^Values are the mean ± SD. All experiments were performed at least three times.

In the first category, the compounds**3a–z** were classified into three series according to *N*-aryl-pyrazole-3-carboxamide moiety: 1) unsubstituted derivatives **3a−i**, 2) 4-methoxyphenyl derivatives **3j−r**, 3) 4-chlorophenyl derivatives **3s−z**.

Among the 6-amino-pyrazolo[1,5-*a*]pyrimidines **3a**−**i**, compound **3d** with 4-CH_3_ substitutent on the 5-aryl and 4-Br substituent on the 7-aryl ring showed the most inhibitory activity in this series (IC_50_ = 15.2 ± 0.4 µM). It is worth mentioning that this derivative showed the highest anti-*α*-glucosidase potency among all the synthesized compounds. Removal of the methyl group from 5-phenyl ring (compounds **3a** and **3b**) and also replacement of bromine with chlorine atom (compound **3c**) led to the significant decrease in inhibitory activity. 4-OCH_3_ substituent on the 5-phenyl ring resulted into a considerable deterioration in activity (compounds **3e** and **3f**). Compound **3g** with 4-Cl substituent on the 7-phenyl ring showed low activity (IC_50_ = 75.6 ± 5.0). Adding a chlorine atom to 5-position of phenyl ring, or replacing it with a heterocycle caused very good effect on the observed activities (compounds **3h** and **3i**).

In the second series, the 4-OCH_3_ substituted *N*-phenyl-pyrazolo-5-carboxamides **3j-r**, compound **3m**, which is the analog of compound **3d**, showed the best activity against *α*-glucosidase. There was the same trend for the activities of compounds **3j-l** with their analog in the first series. Compounds **3n** and **3o** with 4-OCH_3_ substituted 5-phenyl ring showed a moderate activity. The replacement of methoxy group with chlorine atom at 4-position of 5-phenyl ring led to the better performance (compounds **3p** and **3q**). Introducing a heterocycle on the 7-phenyl ring has a good effect to increase activity (compound **3r**).

Among the synthesized derivatives in third series, compound **3v** was found to be the most potent compound. Same as two previous series, removal of methyl group or addition of chlorine atom (compounds **3s-v**) had a destructive effect on the observed inhibitory activities. It was found that introduction an electron-donating group (OCH_3_) on the 4-postion of 5-phenyl ring (compound **3w**) causes a decrease in activity against *α*-glucosidase (IC_50_ = 53.7 ± 3.4). Finally, 4-Cl substituted 5-phenyl ring derivatives **3x-z** were investigated. Compound **3x** with unsubstituted 7-phenyl ring showed a weak inhibitory activity (IC_50_ = 80.3 ± 5.2). Introducing another chlorine atom to 4-position of this ring (compound **3y**), or thiophene (compound **3z**) led to a significant increase in inhibitory activity.

In the second category, the compound **3af** with 4-OCH_3_ and 4-Cl substituents on the respectively 5 and 7-phenyl rings showed the highest potency against the *α*-glucosidase. Further changes on this compound like removing and replacing 4-OCH_3_ with 4-CH_3_ and 4-Cl on the 5-phenyl ring (compounds **3aa**, **3ac** and **3ah**) as well as removing chlorine from 7-phenyl rings (compound **3ae**) made notable increase in IC_50_ value. Compound **3ag** with 4-Cl on the 5-phenyl ring was the weakest compound in this series. Addition of another chlorine atom to 4-position of this ring (compound **3ah**), or thiophene (compound **3ai**) improved the inhibition activities.

Thorough the comparison of IC_50_ values of synthesized**3a–z** with their analog **3aa-ai**, it can be found that substituents on the pyrazole moiety played a substantial role on the observed *α*-glucosidase inhibitory activities. Although the presence of 4-OCH_3_ on the 5-phenyl ring had destructive effect in the first category, the compounds containing this group showed the highest activities in the second category.

### Enzyme kinetic studies

**Figure 2:**
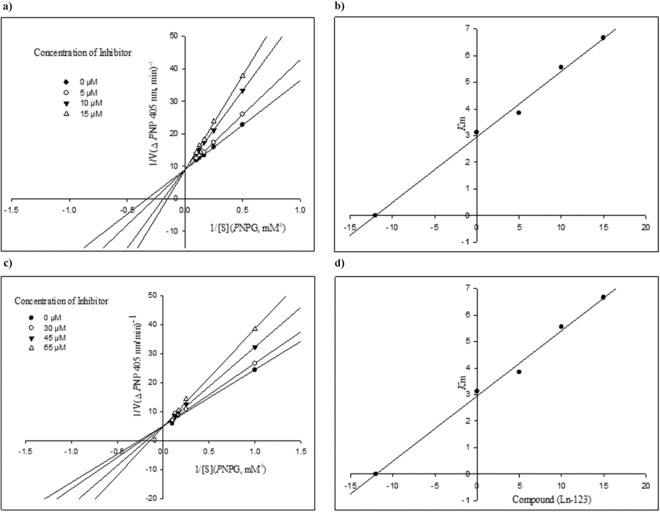
Enzyme Kinetic Studies: (**a**) The Lineweaver–Burk plot in the absence and presence of different concentrations of compound **3d**. (**b**) The secondary plot between *K*_m_ and various concentrations of compound **3d**. (**c**) The Lineweaver–Burk plot in the absence and presence of different concentrations of compound **3af**. (**d**) The secondary plot between *K*_m_ and various concentrations of compound **3af**.

The inhibition mode of the synthesized compounds **3** against *α*-glucosidase was investigated. For this purpose, kinetics analysis was carried out with reference drug, acarbose, and the most potent derivative in each category (**3d** and **3af**). The inhibition type was indicated on the basis of Michaelis-Menten and Lineweaver-Burk plots. As it can be seen in the Lineweaver-Burk plot of selected compounds (Fig. 2), with increasing inhibitor concentrations, the *K*_m_ value gradually increased while *V*_*max*_ value remained unchanged which indicated competitive inhibition. Accordingly, this study revealed both **3d** and **3af** compete with acarbose for binding to the enzyme active site. Furthermore, plot of the *K*_m_ versus different concentration of inhibitor gave an estimate of the inhibition constant, *K*_i_ of 12 µM and 65 µM for compounds **3d** and **3af**, respectively.

### In-silico ADME evaluation

The ADME properties for some of the synthesized highly substituted 6-amino-pyrazolo[1,5-*a*]pyrimidines **3** were computed using Swiss ADME online (http://www.swissadme.ch/index.php) toolkit^76^. Through this in-Silico study under the Lipniski’s rule, five determined drug-likeness parameters were compared with the known drugs^77^. These evaluated parameters are summarized in Table 3. On the basis of MW (<500), HBA (≤10), HBD (<5), and log P (<5) values, the good oral bioavailability of the selected compounds can be estimated. Lipophilicity is determined by Log P in which P is the octanol-water partition coefficient. As it can be seen in Table 3, all the studied compounds have the Log P values in the desirable range. The molecular flexibility can be proved regarding the number of rotatable bonds which should be less than 10 (nROTB <10) and regarding to Table 3, all the obtained numbers are 6 and 7. Topological polar surface area (TPSA) can reveal the surface contribution of polar fragments. The high value of TPSA (>140 Å^2^) may show low blood-brain barrier (BBB) penetration, and therefore, poor membrane permeability^78^. As it can be seen in Table 3, the TPSA values of the tested compounds are in the range 82.51–110.75 Å^2^ exhibiting their permeability in the cellular plasma membrane. The total number of hydrogen bond donors (HBD) should be <5, and the total number of hydrogen bond acceptors (HBA) should be ≤10. All compounds reveal HBDs of 1 and HBAs of 6 and 7 values. Additionally, according to the Veber rule^79^, the number of rotatable bonds should be ≤10 and TPSA <140 Å^2^, or sum of HBD and HBA<12, therefore, all compounds were shown to have good oral bioavailability. Finally, in consistent with ADME predictions, all the studied compounds were proved to have positive drug-likeness values.

**Table 3:** Computed ADME properties for the compounds **3**^**a**^.

Code	MW (g/mol)	HBA	HBD	nROTB	Log *P*_*o/w*_ (iLOGP)	Log *P*_*o/w*_ (mLOGP)	TPSA (Å^2^)	Bioavailability Score	Drug-likeness
**3aa**	468.93	4	1	6	4.27	4.49	82.51	0.55	Yes
**3ac**	482.96	4	1	6	4.34	4.68	82.51	0.55	Yes
**3ae**	464.53	5	1	7	4.07	3.69	91.74	0.55	Yes
**3af**	498.96	5	1	7	4.19	4.15	91.74	0.55	Yes
**3ag**	468.94	4	1	6	4.25	4.49	82.51	0.55	Yes
**3ai**	474.97	4	1	6	4.22	4.12	110.75	0.55	Yes

### Cytotoxicity studies

The cytotoxicity of some of potent compounds including **3d**, **3m**, **3v**, and **3af** was evaluated through use of the breast cancer cell line MDA-MB-231 and human pancreatic cancer cell line PANC-1. The results proved that at concentration of 100 µM, these selected compounds did not possess any cytotoxic activity against the mentioned cell lines (IC_50_ > 200 µM).

### Molecular docking studies

Since there was not any X-ray crystallographic structure of the *Saccharomyces cerevisiae α*-glucosidase in the RCSB protein data bank, a homology modeling method was performed by Auto Dock Tools (version 1.5.6) to study ligand-enzyme interactions^21,80^. Briefly, crystal structures of isomaltase from *Saccharomyces cerevisiae* (PDB code 3A4A), with 72% identical and shares 85% similarity with the *Saccharomyces cerevisiae α*-glucosidase, was designated for building modeled *α*-glucosidase. Afterward, the interaction modes of acarbose as standard inhibitor and the most potent compound in each category **3d** and **3af** in the active site of α-glucosidase were studied.

**Figure 3:**
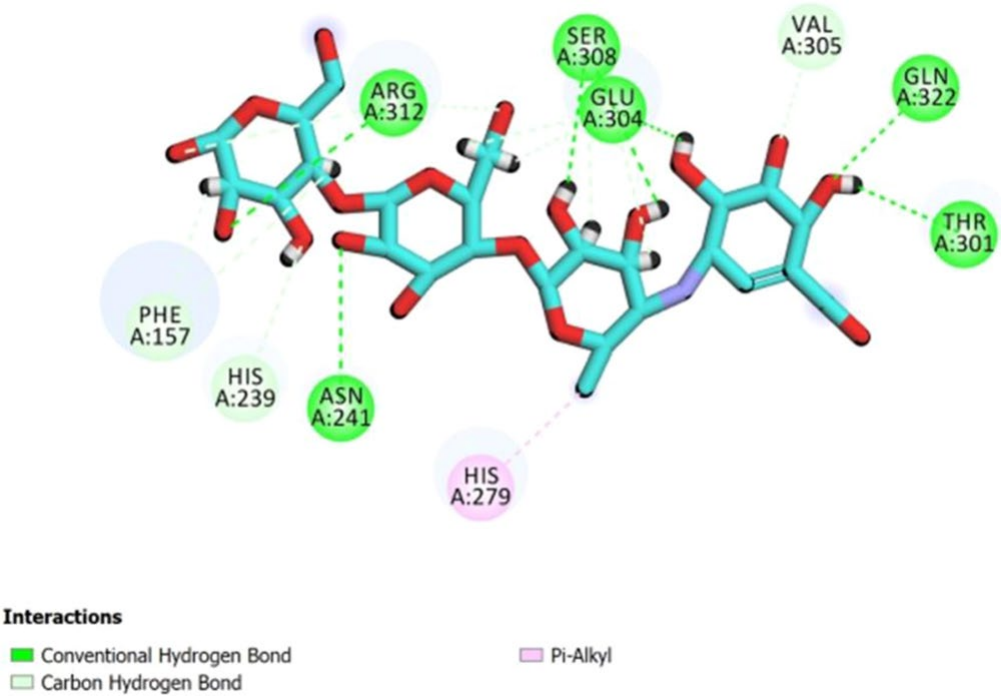
The predicted binding mode of acarbose in the active site pocket.

**Figure 4:**
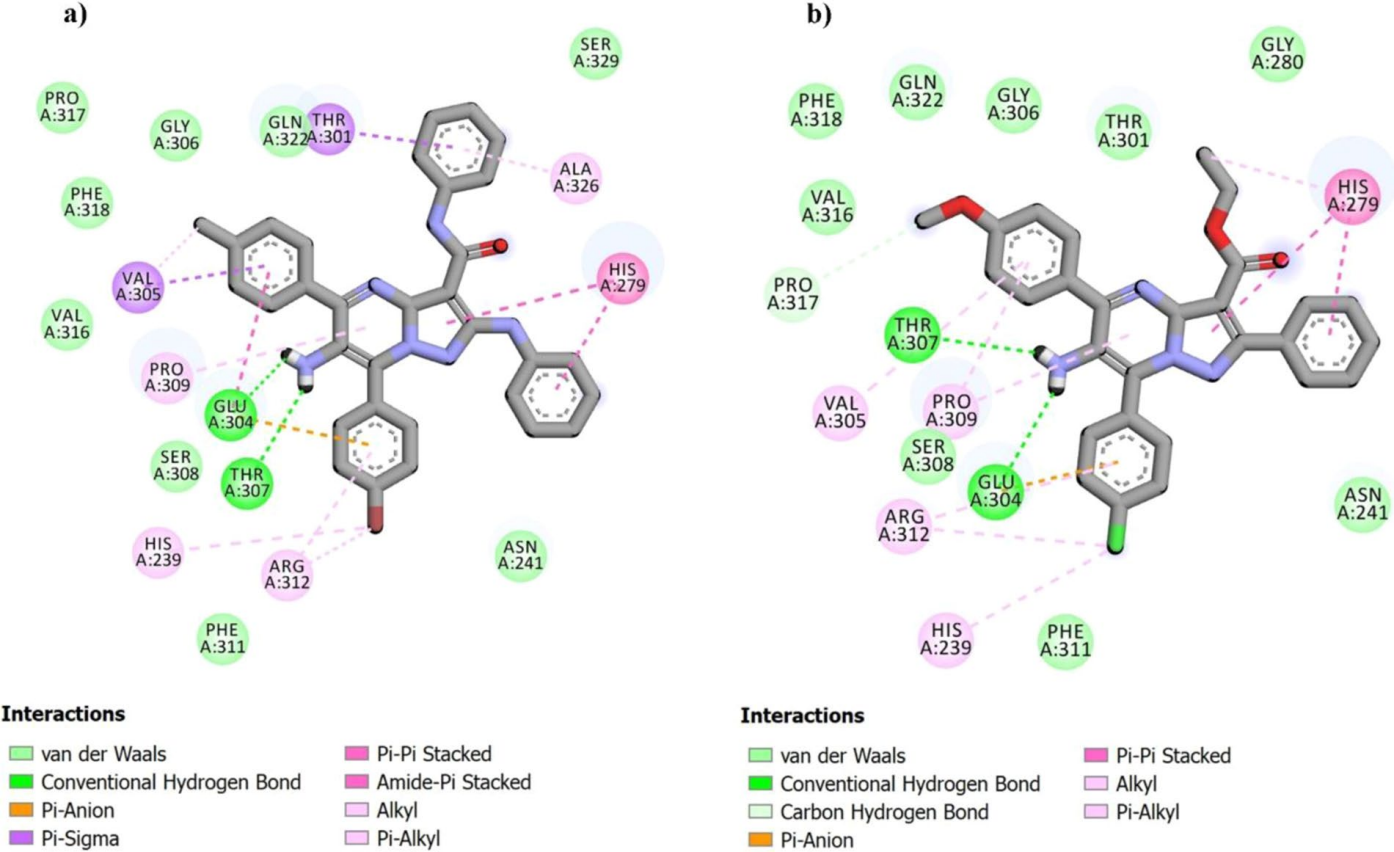
The predicted binding modes of compounds (**a**) **3d** and (**b**) **3af** in the active site pocket.

As shown in Fig. 3, acarbose formed interactions with Asn241, His279, Glu304, Arg312, Thr302, Thr307, Ser308, and Gln322 residues in the enzyme active site. For the most active compound **3d**, amino group established hydrogen bonds with active site residues Thr307 and Glu304. Furthermore, 4-bromo phenyl moiety formed a π-anion interaction with Glu304. Several hydrophobic interactions were also observed with the active site residues His239, Pro309, Arg312 and Ala326. The interactions of **3d** are shown in Fig. 4a. In the case of **3af** (Fig. 4b), hydrogen bonds between amino group and the active site residues Thr307 and Glu304 were formed. Glu304 interacted with 4-choloro phenyl moiety to form π-anion interaction. In addition, several hydrophobic interactions were observed between His239, Val305, Pro309, and Arg312 and 4-choloro phenyl moiety. Further studies on binding energies of compounds **3d**, **3af** and acarbose revealed that they have lower free binding energy (**3d**: −10.0 kcal/mol and **3af**: −9.57 kcal/mol) than acarbose (−4.04 kcal/mol). This means they can bond easier to the target enzyme in comparison to acarbose.

### Conclusion

In conclusion, we have represented a Michael-addition-cyclocondensation reaction between *α*-azidochalcones and 3-aminopyrazoles to prepare a novel library of fully substituted pyrazolo[1,5-*a*]pyrimidines and evaluated their *α*-glucosidase activities. Providing an efficient, simple protocol from readily available starting materials, this method led to new 6-amino-pyrazolo[1,5-*a*]pyrimidines in short time and under the mild conditions. Additionally, easy work-up without any need for chromatography purification processes and really good product yields are the significant features of this proposed reaction. The synthesized compounds were investigated by *α*-glucosidase inhibitory activity assay. All of them showed very good to excellent activities in comparison to the standard drug. Among these derivatives, **3d** was the most potent one with IC_50_ value of 15.2±0.4 µM. The kinetic analysis for the most active compound from each category (**3d** and **3af**) compound showed that there was a competitive mechanism to inhibit *α*-glucosidase. Furthermore, docking studies for these products revealed there were several interactions between desired compounds and important amino acids in the active site of the enzyme.

## Experimental

### Methods

All chemicals were purchased from Merck (Germany) and were used without further purification. Melting points were measured on an Electrothermal 9100 apparatus. Elemental analyses for C, H and N were performed using a Heraeus CHN-O-Rapid analyzer. Mass spectra were recorded on an Agilent Technologies (HP) 5973 mass spectrometer operating at an ionization potential of 20 eV. IR spectra were recorded on a Shimadzu IR-460 spectrometer. ^1^H and ^13^C NMR spectra were measured (in chloroform (CDCl_3_) and dime-thyl sulfoxide (DMSO-*d*_6_) solutions) with Bruker DRX-500 AVANCE (at 500.1 and 125.8 MHz) instruments. α-azidochalcones **1** as well as 3-amino-*N*-aryl-5-(phenylamino)-1*H*-pyrazole-4-carboxamides **2a–c** and ethyl 3-amino-5-phenyl-1*H*-pyrazole-4-carboxylate **2d** were obtained from synthetic methods reported in the literature^81–83^.

## General Procedure for the Synthesis of Highly Substituted 6-Amino-N,5,7-Triaryl-2-(Phenylamino)Pyrazolo[1,5-a]Pyrimidine-3-Carboxamide (3a-z)

A solution of α-azidochalcone **1** (1 mmol), 3-amino-*N*-aryl-5-(phenylamino)-1*H*-pyrazole-4-carboxamide **2a–c** (1.5 mmol), and sodium hydroxide (NaOH) (1.5 mmol) in *N*,*N*-dimethyl formaldehyde DMF (5 mL) was stirred at 80 °C for 10 min. After completion of the reaction according to TLC analysis, the obtained mixture was cooled down to ambient temperature. Then, water was added (10 mL) and extracted three times with ethylacetate (EtOAc) (15 mL for each time). The combined organic extracts were washed with brine, dried over Na_2_SO_4_, and then concentrated. The precipitated product was filtered and recrystallized in 5:1 *n*-Hexane/EtOAc to afford pure compound **3a–z**.

### 6-Amino-7-(4-chlorophenyl)-N,5-diphenyl-2-(phenylamino)pyrazolo[1,5-a]pyrimidine-3-carboxamide (3a)

Dark yellow solid; yield: 86%, mp 248–249 °C. IR (KBr) (*ν*_max_/cm^−1^): 3453–3236 (2NH and NH_2_), 1649 (C=O). ^1^H NMR (500.1 MHz, DMSO-*d*_6_): *δ* 9.85 (s, 1H, amide NH), 9.26 (s, 1H, NH), 8.07 (d, *J* = 7.6 Hz, 2H, 2CH), 7.82 (d, *J* = 7.6 Hz, 2H, 2CH), 7.72–7.66 (m, 5H, 5CH), 7.62 (d, *J* = 7.7 Hz, 2H, 2CH), 7.51 (d, *J* = 7.4 Hz, 2H, 2CH), 7.36 (t, *J* = 7.3 Hz, 2H, 2CH), 7.20 (t, *J* = 7.2 Hz, 2H, 2CH), 7.08 (t, *J* = 7.4 Hz, 1H, CH), 6.88 (t, *J* = 7.3 Hz, 1H, CH), 4.51 (s, 2H, NH_2_). ^13^C NMR (125.1 MHz, DMSO-*d*_6_): *δ* 161.7 (C=O), 155.2, 148.8, 140.1, 139.7, 138.2, 135.1, 134.1 and 130.6 (8C), 130.0 (2CH), 129.9 (CH), 129.5 (2CH), 128.5 (2CH), 128.41 (2CH), 128.37 (2CH), 128.30 (2CH), 127.9 and 127.5 (2C), 122.6 and 120.2 (2CH), 118.3 (2CH), 116.4 (2CH), 85.2 (C). EI-MS, m/z (%): 533 (M^+ 37^Cl, 27), 531 (M^+ 35^Cl, 75), 438 (100), 413 (13), 307 (8), 265 (15), 244 (7), 203 (8), 151 (9), 138 (13), 117 (12), 104 (21), 93 (96), 77 (53), 66 (57), 51 (10). Anal. Calcd for C_31_H_23_ClN_6_O (531.02): C, 70.12; H, 4.37; N, 15.83. Found: C, 70.19; H, 4.43; N, 15.78%.

### 6-Amino-7-(4-bromophenyl)-N,5-diphenyl-2-(phenylamino)pyrazolo[1,5-a]pyrimidine-3-carboxamide (3b)

Dark yellow solid; yield: 79%, mp 235–237 °C. IR (KBr) (*ν*_max_/cm^−1^): 3436–3258 (2NH and NH_2_), 1656 (C=O). ^1^H NMR (500.1 MHz, DMSO-*d*_6_): *δ* 9.83 (s, 1H, amide NH), 9.25 (s, 1H, NH), 7.99 (d, *J* = 7.3 Hz, 2H, 2CH), 7.88–7.77 (m, 4H, 4CH), 7.73–7.64 (m, 3H, 3CH), 7.62 (d, *J* = 7.1 Hz, 2H, 2CH), 7.50 (d, *J* = 7.3 Hz, 2H, 2CH), 7.37 (t, *J* = 7.3 Hz, 2H, 2CH), 7.20 (t, *J* = 7.0 Hz, 2H, 2CH), 7.08 (t, *J* = 7.4 Hz, 2H, 2CH), 6.88 (t, *J* = 7.2 Hz, 2H, 2CH), 4.48 (s, 2H, NH_2_). ^13^C NMR (125.1 MHz, DMSO-*d*_6_): *δ* 161.7 (C=O), 155.2, 148.8, 140.1, 139.7, 138.2 and 135.4 (6C), 131.3 (2CH), 130.6 (C), 130.2 (2CH), 129.9 (CH), 129.5 (2CH), 128.5 (2CH), 128.4 (2CH), 128.3 (2CH), 127.8, 127.5 and 123.0 (3C), 122.6 and 120.2 (2CH), 118.3 (2CH), 116.4 (2CH), 85.2 (C). Anal. Calcd for C_31_H_23_BrN_6_O (575.47): C, 64.70; H, 4.03; N, 14.60. Found: C, 64.79; H, 3.92; N, 14.72%.

### 6-Amino-7-(4-chlorophenyl)-N-phenyl-2-(phenylamino)-5-p-tolylpyrazolo[1,5-a]pyrimidine-3-carboxamide (3c)

Dark yellow solid; yield: 82%, mp 232–233 °C. IR (KBr) (*ν*_max_/cm^−1^): 3465–3278 (2NH and NH_2_), 1652 (C=O). ^1^H NMR (500.1 MHz, DMSO-*d*_6_): *δ* 9.86 (s, 1H, amide NH), 9.26 (s, 1H, NH), 8.06 (d, *J* = 7.8 Hz, 2H, 2CH), 7.73 (d, *J* = 7.3 Hz, 2H, 2CH), 7.70 (d, *J* = 7.9 Hz, 2H, 2CH), 7.62 (d, *J* = 7.3 Hz, 2H, 2CH), 7.53 (d, *J* = 7.8 Hz, 2H, 2CH), 7.50 (d, *J* = 7.9 Hz, 2H, 2CH), 7.37 (t, *J* = 7.2 Hz, 2H, 2CH), 7.22 (t, *J* = 7.3 Hz, 2H, 2CH), 7.08 (t, *J* = 7.2 Hz, 1H, CH), 6.89 (t, *J* = 7.0 Hz, 1H, CH), 4.47 (s, 2H, NH_2_), 2.48 (s, 3H, CH_3_). ^13^C NMR (125.1 MHz, DMSO-*d*_6_): *δ* 161.7 (C=O), 155.2, 148.7, 140.2, 139.8, 139.7, 138.2, 135.1, 134.1 and 130.8 (9C), 130.0 (2CH), 129.4 (2CH), 128.9 (2CH), 128.5 (2CH), 128.4 (2CH), 128.3 (2CH), 127.9 and 124.5 (2C), 122.6 and 120.2 (2CH), 118.3 (2CH), 116.5 (2CH), 85.2 (C), 20.7 (CH_3_). EI-MS, *m/z* (%): 547 (M^+ 37^Cl, 17), 545 (M^+ 35^Cl, 49), 530 (12), 452 (96), 438 (27), 395 (23), 293 (56), 200 (93), 93 (100), 77 (85), 65 (66), 55 (39), 43 (51). Anal. Calcd for C_32_H_25_ClN_6_O (545.04): C, 70.52; H, 4.62; N, 15.42. Found: C, 70.48; H, 4.56; N, 15.37%.

### 6-Amino-7-(4-bromophenyl)-N-phenyl-2-(phenylamino)-5-p-tolylpyrazolo[1,5-a]pyrimidine-3-carboxamide (3d)

Dark yellow solid; yield: 78%, mp 271–272 °C. ^1^H NMR (500.1 MHz, DMSO-*d*_6_): *δ* 9.86 (s, 1H, amide NH), 9.26 (s, 1H, NH), 7.98 (d, *J* = 7.6 Hz, 2H, 2CH), 7.83 (d, *J* = 7.4 Hz, 2H, 2CH), 7.72 (d, *J* = 7.2 Hz, 2H, 2CH), 7.61 (d, *J* = 7.4 Hz, 2H, 2CH), 7.51 (d, *J* = 7.3 Hz, 2H, 2CH), 7.49 (d, *J* = 7.2 Hz, 2H, 2CH), 7.36 (t, *J* = 7.2 Hz, 2H, 2CH), 7.21 (t, *J* = 7.4 Hz, 2H, 2CH), 7.08 (t, *J* = 7.2 Hz, 1H, CH), 6.89 (t, *J* = 7.1 Hz, 1H, CH), 4.48 (s, 2H, NH_2_), 2.48 (s, 3H, CH_3_). ^13^C NMR (125.1 MHz, DMSO-*d*_6_): *δ* 161.7 (C=O), 155.2, 148.7, 140.2, 139.8, 139.7, 138.2 and 135.5 (7C), 131.3 (2CH), 130.2 (2CH), 130.1 (C), 129.4 (2CH), 128.9 (2CH), 128.5 (2CH), 128.3 (2CH), 128.1, 127.8 and 122.9 (3C), 122.5 and 120.2 (2CH), 118.3 (2CH), 116.4 (2CH), 85.2 (C), 20.7 (CH_3_). Anal. Calcd for C_32_H_25_BrN_6_O (589.49): C, 65.20; H, 4.27; N, 14.26. Found: C, 65.28; H, 4.18; N, 14.38%.

### 6-Amino-5-(4-methoxyphenyl)-N,7-diphenyl-2-(phenylamino)pyrazolo[1,5-a]pyrimidine-3-carboxamide (3e)

Dark yellow solid; yield: 68%, mp 257–259 °C. ^1^H NMR (500.1 MHz, DMSO-*d*_6_): *δ* 9.93 (s, 1H, amide NH), 9.23 (s, 1H, NH), 8.02 (d, *J* = 7.4 Hz, 2H, 2CH), 7.81 (d, *J* = 7.0 Hz, 2H, 2CH), 7.70–7.57 (m, 5H, 5CH), 7.56 (d, *J* = 7.0 Hz, 2H, 2CH), 7.35 (t, *J* = 7.1 Hz, 2H, 2CH), 7.28–7.16 (m, 4H, 4CH), 7.07 (t, *J* = 7.1 Hz, 2H, 2CH), 6.89 (t, *J* = 7.2 Hz, 2H, 2CH), 4.40 (s, 2H, NH_2_), 3.91 (s, 3H, OCH_3_). ^13^C NMR (125.1 MHz, DMSO-*d*_6_): *δ* 161.7 (C=O), 155.1, 154.4, 149.7, 140.3, 139.7, 138.2 and 136.3 (7C), 131.2 (2CH), 130.6 (C), 129.9 (CH), 128.5 (2CH), 128.4 (2 × 2CH), 128.0 (2CH), 127.7 (C), 122.5 and 120.1 (2CH), 119.3 (C), 118.2 (2CH), 116.4 (2CH), 113.7 (2CH), 85.1 (C), 54.9 (OCH_3_). Anal. Calcd for C_32_H_26_N_6_O_2_ (526.60): C, 72.99; H, 4.98; N, 15.96. Found: C, 73.08; H, 4.82; N, 16.02%.

### 6-Amino-7-(4-chlorophenyl)-5-(4-methoxyphenyl)-N-phenyl-2-(phenylamino)pyrazolo[1,5-a]pyrimidine-3-carboxamide (3f)

Dark yellow solid; yield: 73%, mp 237–238 °C. IR (KBr) (*ν*_max_/cm^–1^): 3446–3218 (2NH and NH_2_), 1658 (C=O). ^1^H NMR (500.1 MHz, DMSO-*d*_6_): *δ* 9.84 (s, 1H, amide NH), 9.25 (s, 1H, NH), 8.04 (d, *J* = 7.7 Hz, 2H, 2CH), 7.80 (d, *J* = 7.8 Hz, 2H, 2CH), 7.68 (d, *J* = 7.4 Hz, 2H, 2CH), 7.62 (d, *J* = 7.8 Hz, 2H, 2CH), 7.54 (d, *J* = 7.5 Hz, 2H, 2CH), 7.42–7.15 (m, 6H, 6CH), 7.08 (t, *J* = 7.1 Hz, 1H, CH), 6.89 (t, *J* = 7.2 Hz, 1H, CH), 4.44 (s, 2H, NH_2_), 3.91 (s, 3H, OCH_3_). ^13^C NMR (125.1 MHz, DMSO-*d*_6_): *δ* 161.7 (C=O), 155.2, 154.9, 148.4, 140.2, 139.7, 138.2, 135.1 and 134.1 (8C) 131.2 (2CH) 130.8 (C), 130.0 (2CH), 128.53 (2CH), 128.49 (2CH), 128.35 (2CH), 127.8 (C), 122.5 and 120.1 (2CH), 119.2 (C), 118.3 (2CH), 116.4 (2CH), 113.7 (2CH), 85.1 (C), 54.9 (OCH_3_). Anal. Calcd for C_32_H_25_ClN_6_O_2_ (561.01): C, 68.51; H, 4.49; N, 14.98. Found: C, 68.58; H, 4.62; N, 15.04%.

### 6-Amino-5-(4-chlorophenyl)-N,7-diphenyl-2-(phenylamino)pyrazolo[1,5-a]pyrimidine-3-carboxamide (3g)

Dark yellow solid; yield: 90%, mp 285–286 °C. ^1^H NMR (500.1 MHz, DMSO-*d*_6_): *δ* 9.89 (s, 1H, amide NH), 9.24 (s, 1H, NH), 8.00 (d, *J* = 7.4 Hz, 2H, 2CH), 7.87 (d, *J* = 7.3 Hz, 2H, 2CH), 7.73 (d, *J* = 7.3 Hz, 2H, 2CH), 7.68–7.56 (m, 5H, 5CH), 7.50 (d, *J* = 7.2 Hz, 2H, 2CH), 7.35 (t, *J* = 7.6 Hz, 2H, 2CH), 7.22 (t, *J* = 7.4 Hz, 2H, 2CH), 7.07 (t, *J* = 7.4 Hz, 1H, CH), 6.89 (t, *J* = 7.3 Hz, 1H, CH), 4.53 (s, 2H, NH_2_). ^13^C NMR (125.1 MHz, DMSO-*d*_6_): *δ* 161.7 (C=O), 155.1, 150.1, 140.0, 139.7, 138.2, 136.1 and 134.4 (7C), 131.6 (2CH), 129.5 (CH), 129.0 (C), 128.5 (2CH), 128.43 (2CH), 128.41 (2CH), 128.3 (2CH), 128.0 (2CH), 127.6 and 126.5 (2C), 122.5 and 120.2 (2CH), 118.2 (2CH), 116.4 (2CH), 85.3 (C). Anal. Calcd for C_31_H_23_ClN_6_O (561.02): C, 70.11; H, 4.36; N, 15.82. Found: C, 70.05; H, 4.58; N, 15.94%.

### 6-Amino-5,7-bis(4-chlorophenyl)-N-phenyl-2-(phenylamino)pyrazolo[1,5-a]pyrimidine-3-carboxamide (3h)

Dark yellow solid; yield: 86%, mp 281–283 °C. ^1^H NMR (500.1 MHz, DMSO-*d*_6_): *δ* 9.79 (s, 1H, amide NH), 9.23 (s, 1H, NH), 8.03 (d, *J* = 7.6 Hz, 2H, 2CH), 7.85 (d, *J* = 7.4 Hz, 2H, 2CH), 7.75–7.64 (m, 4H, 4CH), 7.60 (d, *J* = 7.3 Hz, 2H, 2CH), 7.49 (d, *J* = 7.4 Hz, 2H, 2CH), 7.38 (t, *J* = 7.3 Hz, 2H, 2CH), 7.21 (d, *J* = 7.2 Hz, 2H, 2CH), 7.07 (t, *J* = 7.1 Hz, 1H, 1CH), 6.88 (t, *J* = 7.0 Hz, H, CH), 4.57 (s, 2H, NH2). ^13^C NMR (125.1 MHz, DMSO-*d*_6_): *δ* 161.6 (C=O), 155.1, 148.9, 140.0, 139.6, 138.1, 135.0, 134.4 and 134.2 (8C), 131.6 (2CH), 131.1 (C), 130.3 (2CH), 128.5 (2CH), 128.44 (2CH), 128.40 (2CH), 128.3 (2CH), 127.7 and 126.4 (2C), 122.5 and 120.2 (2CH), 118.3 (2CH), 116.4 (2CH), 85.3 (C). Anal. Calcd for C_31_H_22_Cl_2_N_6_O (565.46): C, 65.85; H, 3.92; N, 14.86. Found: C, 65.93; H, 4.02; N, 14.98%.

### 6-Amino-5-(4-chlorophenyl)-N-phenyl-2-(phenylamino)-7-(thiophen-2-yl)pyrazolo[1,5-a]pyrimidine-3-carboxamide (3i)

Dark yellow solid; yield: 69%, mp 270–271 °C. IR (KBr) (*ν*_max_/cm^–1^): 3453–3216 (2NH and NH_2_), 1643 (C=O). ^1^H NMR (500.1 MHz, DMSO-*d*_6_): *δ* 9.80 (s, 1H, amide NH), 9.25 (s, 1H, NH), 8.16–8.12 (m, 1H, CH), 7.96 (d, *J* = 4.0 Hz, 1H, CH), 7.82 (d, *J* = 7.8 Hz, 2H, 2CH), 7.80 (d, *J* = 7.6 Hz, 2H, 2CH), 7.75 (d, *J* = 7.6 Hz, 2H, 2CH), 7.47 (d, *J* = 7.5 Hz, 2H, 2CH), 7.41 (t, *J* = 7.1 Hz, 2H, 2CH), 7.32 (t, *J* = 3.3 Hz, 1H, CH), 7.21 (t, *J* = 7.0 Hz, 2H, 2CH), 7.11 (t, *J* = 7.0 Hz, 1H, CH), 6.88 (t, *J* = 7.0 Hz, 1H, CH), 4.74 (s, 2H, NH_2_). ^13^C NMR (125.1 MHz, DMSO-*d*_6_): *δ* 161.7 (C=O), 155.4, 143.8, 140.4, 139.8, 139.6, 138.3 and 134.5 (7 C), 131.7 (2CH), 131.0, 130.5 and 129.5 (3CH), 128.6 (2CH), 128.5 (2CH+C), 128.3 (2CH), 126.8 and 126.3 (2C), 122.6 and 120.2 (2CH), 118.1 (2CH), 116.5 (2CH), 84.8 (C). Anal. Calcd for C_29_H_21_ClN_6_OS (537.04): C, 64.86; H, 3.94; N, 15.65. Found: C, 64.79; H, 4.00; N, 15.73%.

### 6-Amino-7-(4-chlorophenyl)-N-(4-methoxyphenyl)-5-phenyl-2-(phenylamino)pyrazolo[1,5-a]pyrimidine-3-carboxamide (3j)

Dark yellow solid; yield: 81%, mp 217–218 °C. ^1^H NMR (500.1 MHz, DMSO-*d*_6_): *δ* 9.69 (s, 1H, amide NH), 9.28 (s, 1H, NH), 8.05 (d, *J* = 7.5 Hz, 2H, 2CH), 7.82 (d, *J* = 7.3 Hz, 2H, 2CH), 7.72–7.62 (m, 5H, 5CH), 7.53 (d, *J* = 7.4 Hz, 2H, 2CH), 7.49 (d, *J* = 7.1 Hz, 2H, 2CH), 7.19 (d, *J* = 7.1 Hz, 2H, 2CH), 6.93 (d, *J* = 7.0 Hz, 2H, 2CH), 6.86 (t, *J* = 7.3 Hz, 1H, CH), 4.45 (s, 2H, NH_2_), 3.75 (s, 3H, OCH_3_). ^13^C NMR (125.1 MHz, DMSO-*d*_6_): *δ* 161.4 (C=O), 155.1, 154.7, 148.7, 140.1, 139.7, 135.1, 134.1 and 130.5 (8C), 130.0 (2CH), 129.9 (CH), 129.5 (2CH), 128.4 (2×2CH), 128.3 (2CH), 127.7 and 127.5 (2C), 120.1 (CH), 119.8 (2CH), 116.4 (2CH), 113.7 (2CH), 113.2 and 85.2 (2C), 54.7 (OCH_3_). Anal. Calcd for C_32_H_25_ClN_6_O_2_ (561.04): C, 68.50; H, 4.49; N, 14.98. Found: C, 68.43; H, 4.53; N, 15.08%.

### 6-Amino-7-(4-bromophenyl)-N-(4-methoxyphenyl)-5-phenyl-2-(phenylamino)pyrazolo[1,5-a]pyrimidine-3-carboxamide (3k)

Dark yellow solid; yield: 78%, mp 256–258 °C. ^1^H NMR (500.1 MHz, DMSO-*d*_6_): *δ* 9.70 (s, 1H, amide NH), 9.28 (s, 1H, NH), 8.05 (d, *J* = 7.6 Hz, 2H, 2CH), 7.81 (d, *J* = 7.4 Hz, 2H, 2CH), 7.78–7.60 (m, 5H, 5CH), 7.55 (d, *J* = 7.4 Hz, 2H, 2CH), 7.50 (d, *J* = 7.2 Hz, 2H, 2CH), 7.20 (d, *J* = 7.1 Hz, 2H, 2CH), 6.94 (d, *J* = 7.4 Hz, 2H, 2CH), 6.87 (t, *J* = 7.2 Hz, 1H, CH), 4.48 (s, 2H, NH_2_), 3.75 (s, 3H, OCH_3_). ^13^C NMR (125.1 MHz, DMSO-*d*_6_): *δ* 161.4 (C=O), 155.1, 154.7, 148.8, 140.1, 139.7 and 135.5 (6C), 131.3 (2CH), 130.6 (C), 130.2 (2CH), 129.9 (CH), 129.5 (2CH), 128.4 (2CH), 128.3 (2CH), 127.7, 127.5 and 122.9 (3C), 120.1 (CH), 119.9 (2CH), 116.4 (2CH), 113.7 (2CH), 113.2 and 85.2 (2C), 54.7 (OCH_3_). Anal. Calcd for C_32_H_25_BrN_6_O_2_ (605.49): C, 63.48; H, 4.16; N, 13.88. Found: C, 63.36; H, 4.22; N, 14.02%.

### 6-Amino-7-(4-chlorophenyl)-N-(4-methoxyphenyl)-2-(phenylamino)-5-p-tolylpyrazolo[1,5-a]pyrimidine-3-carboxamide (3l)

Dark yellow solid; yield: 89%, mp 263–265 °C. 3472–3253 (2NH and NH_2_), 1672 (C=O). ^1^H NMR (500.1 MHz, DMSO-*d*_6_): *δ* 9.69 (s, 1H, amide NH), 9.26 (s, 1H, NH), 8.03 (d, *J* = 7.7 Hz, 2H, 2CH), 7.72 (d, *J* = 7.2 Hz, 2H, 2CH), 7.67 (d, *J* = 7.5 Hz, 2H, 2CH), 7.56–7.47 (m, 6H, 6CH), 7.20 (d, *J* = 7.4 Hz, 2H, 2CH), 6.93 (d, *J* = 7.5 Hz, 2H, 2CH), 6.88 (t, *J* = 7.1 Hz, 1H, CH), 4.42 (s, 2H, NH_2_), 3.75 (s, 3H, OCH_3_), 2.47 (s, 3H, CH_3_). ^13^C NMR (125.1 MHz, DMSO-*d*_6_): *δ* 161.4 (C=O), 155.1, 154.7, 148.6, 140.2, 139.7, 139.6, 135.1, 134.1 and 130.7 (9C), 130.0 (2CH), 129.3 (2CH), 128.9 (2CH), 128.4 (2×2CH), 127.7 and 124.5 (2C), 120.1 (CH), 119.9 (2CH), 116.4 (2CH), 113.7 (2CH), 113.4 and 85.1 (2C), 54.7 (OCH_3_), 20.7 (CH_3_). Anal. Calcd for C_33_H_27_ClN_6_O_2_ (575.07): C, 68.92; H, 9.89; N, 14.61. Found: C, 68.96; H, 9.93; N, 14.72%.

### 6-Amino-7-(4-bromophenyl)-N-(4-methoxyphenyl)-2-(phenylamino)-5-p-tolylpyrazolo[1,5-a]pyrimidine-3-carboxamide (3m)

Dark yellow solid; yield: 75%, mp 282–283 °C. ^1^H NMR (500.1 MHz, DMSO-*d*_6_): *δ* 9.70 (s, 1H, amide NH), 9.29 (s, 1H, NH), 7.97 (d, *J* = 7.6 Hz, 2H, 2CH), 7.82 (d, *J* = 7.4 Hz, 2H, 2CH), 7.73 (d, *J* = 7.6 Hz, 2H, 2CH), 7.57–7.44 (m, 6H, 6CH), 7.21 (d, *J* = 7.4 Hz, 2H, 2CH), 6.94 (d, *J* = 7.7 Hz, 2H, 2CH), 6.88 (t, *J* = 7.1 Hz, 1H, CH), 4.42 (s, 2H, NH_2_), 3.75 (s, 3H, OCH3), 2.48 (s, 3H, CH3). ^13^C NMR (125.1 MHz, DMSO-*d*_6_): *δ* 161.4 (C=O), 155.1, 154.7, 148.7, 140.2, 139.7,139.6 and 135.5 (7C), 131.3 (2CH), 130.2 (2CH), 130.1 (C), 129.3 (2CH), 128.9 (2CH), 128.3 (2CH), 128.1, 127.6 and 122.9 (3C), 120.1 (CH), 119.9 (2CH), 116.4 (2CH), 113.7 (2CH), 113.2 and 85.1 (2C), 54.7 (OCH_3_), 20.7 (CH_3_). Anal. Calcd for C_33_H_27_BrN_6_O_2_ (619.52): C, 63.98; H, 4.39; N, 13.57. Found: C, 64.05; H, 4.42; N, 13.68%.

### 6-Amino-N,5-bis(4-methoxyphenyl)-7-phenyl-2-(phenylamino)pyrazolo[1,5-a]pyrimidine-3-carboxamide (3n)

Dark yellow solid; yield: 65%, mp 248–250 °C. ^1^H NMR (500.1 MHz, DMSO-*d*_6_): *δ* 9.74 (s, 1H, amide NH), 9.26 (s, 1H, NH), 7.98 (d, *J* = 7.6 Hz, 2H, 2CH), 7.80 (d, *J* = 7.3 Hz, 2H, 2CH), 7.72–7.56 (m, 7H, 7CH), 7.37–7.16 (m, 4H, 4CH), 6.93 (d, *J* = 7.6 Hz, 2H, 2CH), 6.89 (t, *J* = 7.2 Hz, 1H, CH), 4.38 (s, 2H, NH_2_), 3.89 and 3.75 (2s, 6H, 2OCH3). ^13^C NMR (125.1 MHz, DMSO-*d*_6_): *δ* 161.7 (C=O), 155.1, 155.0, 154.7, 149.7, 140.3, 139.7 and 136.3 (7C), 131.2 (2CH), 130.8 (C), 129.4 (CH), 128.3 (2CH), 128.0 (2CH), 127.7 (C), 120.0 (CH), 119.8 (2CH), 119.3 (C), 118.2 (2CH), 116.3 (2CH), 113.68 (2CH), 113.66 (2CH), 113.3 and 85.1 (2C), 54.9 and 54.7 (2OCH_3_). Anal. Calcd for C_33_H_27_N_6_O_3_ (555.62): C, 71.34; H, 4.90; N, 15.13. Found: C, 71.42; H, 5.03; N, 15.09%.

### 6-Amino-7-(4-chlorophenyl)-N,5-bis(4-methoxyphenyl)-2-(phenylamino)pyrazolo[1,5-a]pyrimidine-3-carboxamide (3o)

Dark yellow solid; yield: 79%, mp 268–269 °C. IR (KBr) (*ν*_max_/cm^–1^): 3456–3219 (2NH and NH_2_), 1663 (C=O). ^1^H NMR (500.1 MHz, DMSO-*d*_6_): *δ* 9.70 (s, 1H, amide NH), 9.27 (s, 1H, NH), 8.04 (d, *J* = 7.3 Hz, 2H, 2CH), 7.79 (d, *J* = 7.6 Hz, 2H, 2CH), 7.67 (d, *J* = 7.5 Hz, 2H, 2CH), 7.57–7.44 (m, 2H, 2CH), 7.50–7.37 (m, 5H, 5CH), 6.93 (d, *J* = 7.4 Hz, 2H, 2CH), 6.88 (t, *J* = 7.3 Hz, 1H, CH), 4.48 (s, 2H, NH_2_), 3.89 and 3.72 (2OCH_3_). ^13^C NMR (125.1 MHz, DMSO-*d*_6_): *δ* 161.4 (C=O), 155.14, 155.09, 154.6, 148.5, 140.2, 139.7, 135.2 and 134.0 (8C), 131.2 (2CH), 131.1 (C), 130.0 (2CH), 128.3 (2×2CH), 127.6 (C), 120.1 (CH), 119.8 (2CH), 119.2 (C), 116.4 (2CH), 113.71 (2CH), 113.68 (2CH), 113.1 and 85.6 (2C), 54.8 and 54.7 (2OCH_3_). Anal. Calcd for C_33_H_27_ClN_6_O_3_ (591.12): C, 67.05; H, 4.60; N, 14.22. Found: C, 66.96; H, 4.72; N, 14.36%.

### 6-Amino-5-(4-chlorophenyl)-N-(4-methoxyphenyl)-7-phenyl-2-(phenylamino)pyrazolo[1,5-a]pyrimidine-3-carboxamide (3p)

Dark yellow solid; yield: 83%, mp 245–246 °C. 3438–3249 (2NH and NH_2_), 1663 (C=O). ^1^H NMR (500.1 MHz, DMSO-*d*_6_): *δ* 9.74 (s, 1H, amide NH), 9.27 (s, 1H, NH), 8.00 (d, *J* = 7.8 Hz, 2H, 2CH), 7.87 (d, *J* = 7.5 Hz, 2H, 2CH), 7.74 (d, *J* = 7.2 Hz, 2H, 2CH), 7.68–7.58 (m, 3H, 3CH), 7.52 (d, *J* = 7.3 Hz, 2H, 2CH), 7.50 (d, *J* = 7.1 Hz, 2H, 2CH), 7.23 (t, *J* = 7.4 Hz, 2H, 2CH), 6.93 (d, *J* = 7.1 Hz, 2H, 2CH), 6.89 (t, *J* = 7.4 Hz, 1H, CH), 4.52 (s, 2H, NH_2_), 3.75 (s, 3H, OCH_3_). ^13^C NMR (125.1 MHz, DMSO-*d*_6_): *δ* 161.4 (C=O), 155.0, 154.7, 150.1, 140.0, 139.7, 136.1 and 134.4 (7C), 131.6 (2CH), 129.5 (CH), 128.9 (C), 128.5 (2CH), 128.41 (2CH), 128.37 (2CH), 128.0 (2CH), 127.9 and 126.5 (2C), 120.1 (CH), 119.9 (2CH), 116.4 (2CH), 113.7 (2CH), 113.2 and 85.2 (2C), 54.7 (OCH_3_). Anal. Calcd for C_32_H_25_ClN_6_O_2_ (561.04): C, 68.51; H, 4.49; N, 14.98. Found: C, 68.59; H, 4.56; N, 14.92%.

### 6-Amino-5,7-bis(4-chlorophenyl)-N-(4-methoxyphenyl)-2-(phenylamino)pyrazolo[1,5-a]pyrimidine-3-carboxamide (3q)

Dark yellow solid; yield: 78%, mp 245–246 °C. ^1^H NMR (500.1 MHz, DMSO-*d*_6_): *δ* 9.67 (s, 1H, amide NH), 9.27 (s, 1H, NH), 8.03 (d, *J* = 7.6 Hz, 2H, 2CH), 7.85 (d, *J* = 7.4 Hz, 2H, 2CH), 7.73 (d, *J* = 7.5 Hz, 2H, 2CH), 7.68 (d, *J* = 7.4 Hz, 2H, 2CH), 7.53 (d, *J* = 7.5 Hz, 2H, 2CH), 7.50 (d, *J* = 7.4 Hz, 2H, 2CH), 7.22 (t, *J* = 7.4 Hz, 2H, 2CH), 6.94 (d, *J* = 7.6 Hz, 2H, 2CH), 6.89 (t, *J* = 7.8 Hz, 1H, CH), 4.56 (s, 2H, NH_2_), 3.76 (s, 3H, OCH_3_). ^13^C NMR (125.1 MHz, DMSO-d6): δ 161.4 (C=O), 155.1, 154.7, 148.9, 140.0, 139.7, 135.0, 134.4 and 134.1 (8C), 131.6 (2CH), 131.2 (C), 130.0 (2CH), 128.5 (2CH), 128.4 (2×2CH), 128.0 and 126.5 (2C), 120.2 (CH), 119.9 (2CH), 116.4 (2CH), 113.7 (2CH), 113.2 and 85.2 (2C), 54.7 (OCH_3_). Anal. Calcd for C_32_H_24_Cl_2_N_6_O_2_ (595.53): C, 64.54; H, 4.07; N, 14.11. Found: C, 64.64; H, 3.98; N, 14.03%.

### 6-Amino-5-(4-chlorophenyl)-N-(4-methoxyphenyl)-2-(phenylamino)-7-(thiophen-2-yl)pyrazolo[1,5-a]pyrimidine-3-carboxamide (3r)

Dark yellow solid; yield: 69%, mp 240–242 °C. ^1^H NMR (500.1 MHz, DMSO-*d*_6_): *δ* 9.67 (s, 1H, amide NH), 9.26 (s, 1H, NH), 8.13 (dd, *J* = 0.6, 3.2 Hz, 1H, CH), 7.92 (d, *J* = 4.0 Hz, 1H, CH), 7.82 (d, *J* = 7.7 Hz, 2H, 2CH), 7.74 (d, *J* = 7.6 Hz, 2H, 2CH), 7.70 (d, *J* = 7.6 Hz, 2H, 2CH), 7.47 (d, *J* = 7.5 Hz, 2H, 2CH), 7.34–7.27 (m, 1H, CH), 7.21 (t, *J* = 7.3 Hz, 2H, 2CH), 6.97 (d, *J* = 7.5 Hz, 2H, 2CH), 6.88 (t, *J* = 7.4 Hz, 1H, 1CH), 4.67 (s, 2H, NH_2_), 3.77 (s, 3H, OCH_3_). ^13^C NMR (125.1 MHz, DMSO-*d*_6_): *δ* 161.3 (C=O), 155.3, 154.7, 143.7, 140.5, 139.8, 139.6 and 134.5 (7C), 131.7 (2CH), 131.4 (CH), 131.0 (C), 13 0.4 and 129.4 (2CH), 128.5 (2CH+C), 128.3 (2CH), 126.6 and 126.3 (2C), 120.2 (CH), 119.6 (2CH), 116.4 (2CH), 113.7 (2CH), 113.4 and 84.8 (2C), 54.7 (OCH_3_). Anal. Calcd for C_30_H_23_ClN_6_O_2_S (567.11): C, 63.54; H, 4.10; N, 14.82. Found: C, 63.58; H, 4.08; N, 14.88%.

### 6-Amino-N,7-bis(4-chlorophenyl)-5-phenyl-2-(phenylamino)pyrazolo[1,5-a]pyrimidine-3-carboxamide (3s)

Dark yellow solid; yield: 92%, mp 274–275 °C. IR (KBr) (*ν*_max_/cm^–1^): 3463–3239 (2NH and NH_2_), 1658 (C=O). IR (KBr) (*ν*_max_/cm^–1^): 3424–3228 (2NH and NH_2_), 1652 (C=O). ^1^H NMR (500.1 MHz, DMSO-*d*_6_): *δ* 9.84 (s, 1H, amide NH), 9.17 (s, 1H, NH), 8.05 (d, *J* = 7.7 Hz, 2H, 2CH), 7.80 (d, *J* = 7.3 Hz, 2H, 2CH), 7.74–7.56 (m, 7H, 7CH), 7.47 (d, *J* = 7.1Hz, 2H, 2CH), 7.37 (d, *J* = 7.9 Hz, 2H, 2CH), 7.18 (t, *J* = 7.1 Hz, 2H, 2CH), 7.68 (t, *J* = 7.4 Hz, 1H, CH), 4.47 (s, 2H, NH_2_). ^13^C NMR (125.1 MHz, DMSO-*d*_6_): *δ* 161.6 (C=O), 155.2, 148.8, 140.1, 139.6, 137.1, 135.0, 134.2 and 130.3 (8C), 130.0 (2CH), 129.9 (CH), 129.4 (2CH), 128.40 (2CH), 128.36 (2CH), 128.35 (2CH), 127.4, 127.3 and 126.1 (3C), 120.2 (CH), 119.85 (2CH), 119.83 (2CH), 116.5 (2CH), 85.1 (C). Anal. Calcd for C_31_H_22_Cl_2_N_6_O (595.53): C, 65.85; H, 3.92; N, 14.86. Found: C, 65.91; H, 3.86; N, 14.94%.

### 6-Amino-7-(4-bromophenyl)-N-(4-chlorophenyl)-5-phenyl-2-(phenylamino)pyrazolo[1,5-a]pyrimidine-3-carboxamide (3t)

Dark yellow solid; yield: 85%, mp 294–296 °C. IR (KBr) (*ν*_max_/cm^−1^): 3453–3219 (2NH and NH_2_), 1658 (C=O). ^1^H NMR (500.1 MHz, DMSO-*d*_6_): *δ* 9.86 (s, 1H, amide NH), 9.18 (s, 1H, NH), 7.99 (d, *J* = 7.2 Hz, 2H, 2CH), 7.83 (d, *J* = 7.5 Hz, 2H, 2CH), 7.81 (d, *J* = 7.4 Hz, 2H, 2CH), 7.73–7.58 (m, 5H, 5CH), 7.49 (d, *J* = 7.2 Hz, 2H, 2CH), 7.40 (d, *J* = 7.4 Hz, 2H, 2CH), 7.19 (t, *J* = 7.1 Hz, 2H, 2CH), 6.88 (t, *J* = 7.3 Hz, 1H, CH), 4.48 (s, 2H, NH_2_). ^13^C NMR (125.1 MHz, DMSO-*d*_6_): *δ* 161.7 (C=O), 155.3, 148.9, 140.2, 139.6, 137.1 and 135.4 (6C), 131.4 (2CH), 130.2 (2CH), 129.9 (CH), 129.4 (2CH), 128.4 (2×2CH), 128.3, 127.7, 127.4, 126.1 and 123.0 (5C), 120.3 (CH), 119.93 (2CH), 119.92 (2CH), 116.5 (2CH), 85.5 (C). Anal. Calcd for C_31_H_22_BrClN_6_O (609.95): C, 61.04; H, 3.64; N, 13.78. Found: C, 60.96; H, 3.72; N, 13.86%.

### 6-Amino-N,7-bis(4-chlorophenyl)-2-(phenylamino)-5-p-tolylpyrazolo[1,5-a]pyrimidine-3-carboxamide (3u)

Dark yellow solid; yield: 85%, mp 281–282 °C. IR (KBr) (*ν*_max_/cm^−1^): 3448–3225 (2NH and NH_2_), 1672 (C=O). ^1^H NMR (500.1 MHz, DMSO-*d*_6_): *δ* 9.90 (s, 1H, amide NH), 9.20 (s, 1H, NH), 8.07 (d, *J* = 7.3 Hz, 2H, 2CH), 7.73 (d, *J* = 7.4 Hz, 2H, 2CH), 7.70 (d, *J* = 7.7 Hz, 2H, 2CH), 7.66 (d, *J* = 7.8 Hz, 2H, 2CH), 7.53 (d, *J* = 7.8 Hz, 2H, 2CH), 7.51 (d, *J* = 7.3 Hz, 2H, 2CH), 7.42 (d, *J* = 7.7 Hz, 2H, 2CH), 7.22 (t, *J* = 7.1 Hz, 2H, 2CH), 6.90 (t, *J* = 7.2 Hz, 1H, CH), 4.51 (s, 2H, NH_2_), 2.49 (s, 3H, CH_3_). ^13^C NMR (125.1 MHz, DMSO-*d*_6_): *δ* 161.6 (C=O), 155.0, 150.1, 140.2, 139.7, 139.6, 137.1, 135.1, 134.1 and 130.8 (9C), 130.0 (2CH), 129.3 (2CH), 128.9 (2CH), 128.43 (2CH), 128.38 (2CH), 127.1, 126.1 and 124.6 (3C), 120.2 (CH), 119.94 (2CH), 119.90 (2CH), 116.5 (2CH), 85.2 (C), 20.8 (CH_3_). Anal. Calcd for C_32_H_24_Cl_2_N_6_O (579.53): C, 66.32; H, 4.18; N, 14.50. Found: C, 66.25; H, 4.24; N, 14.64%.

### 6-Amino-7-(4-bromophenyl)-N-(4-chlorophenyl)-2-(phenylamino)-5-p-tolylpyrazolo[1,5-a]pyrimidine-3-carboxamide (3v)

Dark yellow solid; yield: 82%, mp 271–273 °C. IR (KBr) (*ν*_max_/cm^−1^): 3461–3227 (2NH and NH_2_), 1671 (C=O). ^1^H NMR (500.1 MHz, DMSO-*d*_6_): *δ* 9.88 (s, 1H, amide NH), 9.20 (s, 1H, NH), 7.99 (d, *J* = 7.6 Hz, 2H, 2CH), 7.83 (d, *J* = 7.4 Hz, 2H, 2CH), 7.72 (d, *J* = 7.3 Hz, 2H, 2CH), 7.66 (d, *J* = 7.4 Hz, 2H, 2CH), 7.57–7.46 (m, 4H, 4CH), 7.40 (d, *J* = 7.3 Hz, 2H, 2CH), 7.22 (t, *J* = 7.0 Hz, 2H, 2CH), 6.90 (t, *J* = 7.2 Hz, 1H, CH), 4.49 (s, 2H, NH_2_), 2.48 (s, 3H, CH_3_). ^13^C NMR (125.1 MHz, DMSO-*d*_6_): *δ* 161.7 (C=O), 155.2, 148.6, 140.2, 139.7, 139.6, 137.1 and 135.1 (7C), 131.3 (2CH), 130.3 (2CH), 130.0 (C), 129.3 (2CH), 128.9 (2CH), 128.4 (2CH), 128.0, 127.5, 126.6 and 123.0 (4C), 120.3 (CH), 119.93 (2CH), 119.91 (2CH), 116.5 (2CH), 84.3 (C), 20.7 (CH_3_). Anal. Calcd for C_32_H_24_BrClN_6_O (609.95): C, 61.60; H, 3.88; N, 13.47. Found: C, 61.68; H, 3.94; N, 13.54%.

### 6-Amino-N-(4-chlorophenyl)-5-(4-methoxyphenyl)-7-phenyl-2-(phenylamino)pyrazolo[1,5-a]pyrimidine-3-carboxamide (3w)

Dark yellow solid; yield: 75%, mp 255–257 °C. IR (KBr) (*ν*_max_/cm^−1^): 3468–3226 (2NH and NH_2_), 1656 (C=O). ^1^H NMR (500.1 MHz, DMSO-*d*_6_): *δ* 9.96 (s, 1H, amide NH), 9.18 (s, 1H, NH), 8.03 (d, *J* = 7.5 Hz, 2H, 2CH), 7.82 (d, *J* = 7.2 Hz, 2H, 2CH), 7.74–7.16 (m, 13H, 13CH), 6.88 (t, *J* = 7.3 Hz, 1H, CH), 4.42 (s, 2H, NH_2_), 3.92 (s, 3H, OCH_3_). ^13^C NMR (125.1 MHz, DMSO-*d*_6_): *δ* 161.7 (C=O), 155.0, 154.8, 150.1, 140.3, 139.7, 137.1 and 136.2 (7C), 131.2 (2CH), 130.8 (C), 129.4 (CH), 128.4 (2CH), 128.3 (2CH), 127.6 and 126.8 (2C), 120.2 (CH), 119.88 (2CH), 119.87 (2CH), 119.2 (C), 118.81 (2CH), 116.6 (2CH), 113.7 (2CH), 85.3 (C), 54.9 (OCH_3_). Anal. Calcd for C_32_H_25_ClN_6_O_2_ (561.04): C, 68.51; H, 4.49; N, 14.98. Found: C, 68.46; H, 4.57; N, 15.07%.

### 6-Amino-N,5-bis(4-chlorophenyl)-7-phenyl-2-(phenylamino)pyrazolo[1,5-a]pyrimidine-3-carboxamide (3x)

Dark yellow solid; yield: 89%, mp 255–257 °C. ^1^H NMR (500.1 MHz, DMSO-*d*_6_): *δ* 9.89 (s, 1H, amide NH), 9.16 (s, 1H, NH), 8.00 (d, *J* = 7.5 Hz, 2H, 2CH), 7.85 (d, *J* = 7.3 Hz, 2H, 2CH), 7.72 (d, *J* = 7.4 Hz, 2H, 2CH), 7.68–7.55 (m, 5H, 5CH), 7.48 (d, *J* = 7.2 Hz, 2H, 2CH), 7.36 (d, *J* = 7.5 Hz, 2H, 2CH), 7.21 (t, *J* = 7.2 Hz, 2H, 2CH), 6.89 (t, *J* = 7.0 Hz, 1H, CH), 4.52 (s, 2H, NH_2_). ^13^C NMR (125.1 MHz, DMSO-*d*_6_): *δ* 161.6 (C=O), 155.0, 150.1, 140.0, 139.6, 137.1, 136.0 and 134.4 (7C), 131.6 (2CH), 129.5 (CH), 129.1 (C), 128.4 (2CH), 128.32 (2CH), 128.25 (2CH), 128.0 (2CH), 127.8, 126.4 and 126.0 (3C), 120.2 (CH), 119.73 (2CH), 119.71 (2CH), 116.4 (2CH), 85.5 (C). Anal. Calcd for C_31_H_22_Cl_2_N_6_O (595.53): C, 65.85; H, 3.92; N, 14.86. Found: C, 65.79; H, 4.02; N, 14.78%.

### 6-Amino-N,5,7-tris(4-chlorophenyl)-2-(phenylamino)pyrazolo[1,5-a]pyrimidine-3-carboxamide (3y)

Dark yellow solid; yield: 88%, mp 268–270 °C. ^1^H NMR (500.1 MHz, DMSO-*d*_6_): *δ* 9.87 (s, 1H, amide NH), 9.20 (s, 1H, NH), 8.06 (d, *J* = 7.6 Hz, 2H, 2CH), 7.86 (d, *J* = 7.5 Hz, 2H, 2CH), 7.78–7.58 (m, 4H, 4CH), 7.52–7.33 (m, 6H, 6CH), 7.26 (t, *J* = 7.2 Hz, 2H, 2CH), 6.88 (t, *J* = 7.1 Hz, 2H, 2CH), 4.65 (s, 2H, NH_2_). ^13^C NMR (125.1 MHz, DMSO-*d*_6_): *δ* 161.4 (C=O), 155.1, 148.9, 140.0, 139.7, 137.3, 135.0, 134.4, 134.1 and 131.6 (9C), 131.2 (2CH), 130.0 (2CH), 128.5 (2CH), 128.4 (2CH), 128.0 (2CH), 127.7, 126.5 and 126.2 (3C), 120.2 (CH), 119.92 (2CH), 119.86 (2CH), 116.4 (2CH), 85.2 (C). Anal. Calcd for C_31_H_21_Cl_3_N_6_O (599.91): C, 62.07; H, 3.53; N, 14.01. Found: C, 61.98; H, 3.62; N, 14.12%.

### 6-Amino-N,5-bis(4-chlorophenyl)-2-(phenylamino)-7-(thiophen-2-yl)pyrazolo[1,5-a]pyrimidine-3-carboxamide (3z)

Dark yellow solid; yield: 73%, mp 274–276 °C. ^1^H NMR (500.1 MHz, DMSO-*d*_6_): *δ* 9.78 (s, 1H, amide NH), 9.14 (s, 1H, NH), 8.14–8.05 (m, 1H, CH), 7.95–7.86 (m, 1H, CH), 7.79 (d, *J* = 7.6 Hz, 2H, 2CH), 7.76 (d, *J* = 7.6 Hz, 2H, 2CH), 7.72 (d, *J* = 7.5 Hz, 2H, 2CH), 7.43 (d, *J* = 7.6 Hz, 2H, 2CH), 7.39 (d, *J* = 7.5 Hz, 2H, 2CH), 7.33–7.27 (m, 1H, CH), 7.19 (d, *J* = 7.4 Hz, 2H, 2CH), 6.87 (t, *J* = 7.2 Hz, 1H, CH), 4.67 (s, 2H, NH_2_). ^13^C NMR (125.1 MHz, DMSO-*d*_6_): *δ* 161.5 (C=O), 155.3, 143.7, 140.4, 139.8, 139.5, 137.2 and 134.5 (7C), 131.7 (2CH), 130.9, 130.4 and 129.5 (3CH), 128.5 (2CH+C), 128.3 (2CH), 126.8, 126.2 and 126.0 (3C), 120.2 (CH), 119.60 (2CH), 119.59 (2CH), 116.4 (2CH), 84.7 (C). Anal. Calcd for C_29_H_20_Cl_2_N_6_OS (571.49): C, 60.95; H, 3.53; N, 14.71. Found: C, 61.05; H, 3.68; N, 14.86%.

## General Procedure for the Synthesis of Highly Substituted Ethyl 6-Amino-5,7-Diaryl-2-Phenylpyrazolo[1,5-a]Pyrimidine-3-Carboxylate (3aa-ai)

A mixture of α-azidochalcone **1** (1 mmol), ethyl 3-amino-5-phenyl-1*H*-pyrazole-4-carboxylate **2d** (2 mmol), and triethylamine (Et_3_N) (2 mmol) in tetrahydrofurane (THF) (4 mL) was stirred at ambient temperature for almost 20 min. After completion of the reaction (confirmed by TLC monitoring), the solvent was evaporated under the low pressure. Then, the residue was recrystallized from EtOAc to obtain desirable products **3aa-ai**.

### Ethyl 6-amino-7-(4-chlorophenyl)-2,5-diphenylpyrazolo[1,5-a]pyrimidine-3-carboxylate (3aa)

Pale yellow solid, yield: 87%, mp 180–182 °C. IR (KBr) (*ν*_max_/cm^–1^): 3420 and 3376 (NH_2_), 1723 (C=O). ^1^H NMR (500.1 MHz, CDCl_3_): *δ* 7.94 (d, *J* = 8.5 Hz, 2H), 7.78–7.71 (m, 4H, 4CH), 7.62 (t, *J* = 7.5 Hz, 2H, 2CH), 7.56 (t, *J* = 7.5 Hz, 1H, CH), 7.52 (d, *J* = 8.5 Hz, 2H, 2CH), 7.41–7.36 (m, 3H, 3CH), 4.34 (q, *J* = 7.1 Hz, 2H, OCH_2_), 3.74 (s, 2H, NH_2_), 1.30 (t, *J* = 7.1 Hz, 3H, CH_3_). ^13^C NMR (125.1 MHz, CDCl_3_): *δ* 163.4 (C=O), 157.1, 151.5, 144.9, 136.2, 135.3 and 133.0 (6C), 130.5 (CH+C), 130.4 (2CH), 130.0 (2CH), 129.9 (2CH), 129.4 (2CH), 129.1 (2CH), 128.7 (CH), 127.9 (C), 127.6 (2CH+C), 99.9 (C), 60.0 (OCH_2_), 14.2 (CH_3_). Anal. Calcd for C_27_H_21_ClN_4_O_2_ (468.94): C, 69.16; H, 4.51; N, 11.95. Found: C, 69.23; H, 4.58; N, 12.04%.

### Ethyl 6-amino-7-(4-bromophenyl)-2,5-diphenylpyrazolo[1,5-a]pyrimidine-3-carboxylate (3ab)

Pale yellow solid, yield: 76%, mp 201–202 °C. ^1^H NMR (500.1 MHz, CDCl_3_): *δ* 7.88 (d, *J* = 8.3 Hz, 2H, 2CH), 7.78–7.72 (m, 4H, 4CH), 7.70 (d, *J* = 8.3 Hz, 2H, 2CH), 7.63 (t, *J* = 7.6 Hz, 2H, 2CH), 7.57 (t, *J* = 7.4 Hz, 1H, CH), 7.46–7.37 (m, 3H, 3CH), 4.35 (q, *J* = 7.1 Hz, 2H, OCH_2_), 3.72 (s, 2H, NH_2_), 1.30 (t, *J* = 7.1 Hz, 3H, CH_3_). ^13^C NMR (125.1 MHz, CDCl_3_): *δ* 163.4 (C=O), 157.1, 151.6, 145.0, 135.7 and 133.0 (5C), 132.1 (2CH), 130.63 (2CH), 130.55 (CH), 130.00 (2CH), 129.95 (2CH), 129.8 (C), 129.4 (2CH), 128.7 (CH), 127.9 (C), 127.64 (2CH), 127.56, 124.6 and 100.0 (3C), 60.1 (OCH_2_), 14.2 (CH_3_). EI-MS, *m/z* (%): 516 (M^+ 81^Br, 98), 514 (M^+ 79^Br, 100), 469 (29), 467 (30), 442 (46), 440 (46), 367 (10), 185 (14), 155 (21), 105 (26), 89 (7), 77 (23). Anal. Calcd for C_27_H_21_BrN_4_O_2_ (513.39): C, 63.17; H, 4.12; N, 10.91. Found: C, 63.03; H, 4.04; N, 10.82%.

### Ethyl 6-amino-7-(4-chlorophenyl)-2-phenyl-5-p-tolylpyrazolo[1,5-a]pyrimidine-3-carboxylate (3ac)

Pale yellow solid, yield: 90%, mp 221–223 °C ^1^H NMR (500.1 MHz, CDCl_3_): *δ* 7.94 (d, *J* = 8.5 Hz, 2H, 2CH). 7.75–7.73 (m,2H, 2CH), 7.65 (d, *J* = 8.0 Hz, 2H, 2CH), 7.53 (d, *J* = 8.5 Hz, 2H, 2CH), 7.43 (d, *J* = 8.0 Hz, 2H, 2CH), 7.40–7.36 (m, 3H, 3CH), 4.34 (q, *J* = 7.1 Hz, 2H, OCH_2_), 3.73 (s, 2H, NH_2_), 2.47 (s, 3H, CH_3_), 1.30 (t, *J* = 7.1 Hz, 3H, CH_3_). ^13^C NMR (125.1 MHz, CDCl_3_): *δ* 163.4 (C=O), 157.1, 151.4, 145.0, 140.8, 136.2 and 133.1 (6C), 130.4 (2CH), 130.12 (2CH), 130.05 (C), 129.97 (2CH), 129.8 (2CH), 129.1 (2CH), 128.7 (CH), 127.61 (2CH), 127.56, 126.8, 124.8 and 106.5 (4C), 60.1 (OCH_2_), 21.61 (CH_3_), 14.2 (CH_3_). Anal. Calcd for C_28_H_23_ClN_4_O_2_ (482.97): C, 69.63; H, 4.80; N, 11.60. Found: C, 69.73; H, 4.93; N, 11.65%.

### Ethyl 6-amino-7-(4-bromophenyl)-2-phenyl-5-p-tolylpyrazolo[1,5-a]pyrimidine-3-carboxylate (3ad)

Pale yellow solid, yield: 82%, mp 230–232 °C. IR (KBr) (*ν*_max_/cm^–1^): 3458 and 3356 (NH_2_), 1735 (C=O). ^1^H NMR (500.1 MHz, CDCl_3_): *δ* 7.88 (d, *J* = 8.5 Hz, 2H, 2CH), 7.79–7.72 (m, 2H, 2CH), 7.70 (d, *J* =8.5 Hz, 2H, 2CH), 7.65 (d, *J* = 8.0 Hz, 2H, 2CH), 7.43 (d, *J* = 8.0 Hz, 2H, 2CH), 7.45–7.33 (m, 3H, 3CH), 4.34 (q, *J* =7.1 Hz, 2H, OCH_2_), 3.72 (s, 2H, NH_2_), 2.48 (s, 3H, CH_3_), 1.30 (t, *J* = 7.1 Hz, 3H, CH_3_).^13^C NMR (125.1 MHz, CDCl_3_): *δ* 163.4 (C=O), 157.1, 151.5, 145.0, 140.8, 135.8 and 133.1 (6C), 132.1 (2CH), 130.7 (2CH), 130.1 (2CH), 130.0 (2CH), 129.9 (C), 129.8 (2CH), 128.7 (CH), 127.6 (2CH), 127.5, 124.9, 124.5 and 99.9 (4C), 60.0 (OCH_2_), 21.6 (CH_3_), 14.2 (CH_3_). Anal. Calcd for C_28_H_23_BrN_4_O_2_ (527.42): C, 63.76; H, 4.40; N, 10.62. Found: C, 63.84; H, 4.48; N, 10.78%.

### Ethyl 6-amino-5-(4-methoxyphenyl)-2,7-diphenylpyrazolo[1,5-a]pyrimidine-3-carboxylate (3ae)

Pale yellow solid, yield: 71%, mp 189–190 °C. IR (KBr) (*ν*_max_/cm^−1^): 3443 and 3362 (NH_2_), 1736 (C=O). ^1^H NMR (500.1 MHz, CDCl_3_): *δ* 8.31 (d, *J* = 7.6 Hz, 2H, 2CH), 8.20 (d, *J* = 7.9 Hz, 2H, 2CH), 7.87 (d, *J* = 7.9 Hz, 2H, 2CH), 7.68–7.55 (m, 3H, 3CH), 7.50–7.40 (m, 3H, 3CH), 7.11 (d, *J* = 7.9 Hz, 2H, 2CH), 4.44 (q, *J* = 7.0 Hz, 2H, CH_2_), 3.84 (s, 2H, NH_2_), 3.93 (s, 3H, OCH_3_), 1.42 (t, *J* = 7.0 Hz, 3H, CH_3_). ^13^C NMR (125.1 MHz, CDCl_3_): *δ* 163.5 (C=O), 158.7, 158.4, 150.6, 146.9, 137.1 and 132.9 (6C), 131.4 (2CH), 130.9 (CH), 130.0 (2CH), 129.02 (CH), 128.98 (2CH), 127.8 (2CH), 127.6 (2CH+C), 122.9 and 119.8 (2C), 114.2 (2CH), 105.5 (C), 60.2 (OCH_2_), 55.5 (OCH_3_), 14.3 (CH_3_). Anal. Calcd for C_28_H_24_N_4_O_3_ (464.52): C, 72.40; H, 5.20; N, 12.06. Found: C, 72.48; H, 5.16; N, 11.98%.

### Ethyl 6-amino-7-(4-chlorophenyl)-5-(4-methoxyphenyl)-2-phenylpyrazolo[1,5-a]pyrimidine-3-carboxylate (3af)

Pale yellow solid, yield: 86%, mp 226–228 °C. ^1^H NMR (500.1 MHz, CDCl_3_): *δ* 7.94 (d, *J* = 8.4 Hz, 2H, 2CH), 7.80–7.72 (m, 4H, 4CH), 7.73 (d, *J* = 8.7 Hz, 2H, 2CH), 7.53 (d, *J* = 8.4 Hz, 2H, 2CH), 7.42–7.37 (m, 3H, 3CH), 7.13 (d, *J* = 8.7 Hz, 2H, 2CH), 4.34 (q, *J* = 7.1 Hz, 2H, OCH_2_), 3.92 (s, 3H, OCH_3_), 3.74 (s, 2H, NH_2_), 1.30 (t, *J* = 7.1 Hz, 3H, CH_3_). ^13^C NMR (125.1 MHz, CDCl_3_): *δ* 161.1 (C=O), 157.1, 151.4, 151.0, 145.0, 136.2, 135.3 and 133.1 (7C), 131.6 (2CH), 130.4 (2CH), 129.96 (2CH), 129.93 (C), 129.1 (2CH), 128.7 (CH), 127.62 (2CH), 127.56 and 119.7 (2C), 114.8 (2CH), 100.5 (C), 60.01 (OCH_2_), 55.4 (OCH_3_), 14.2 (CH_3_). Anal. Calcd for C_28_H_23_ClN_4_O_3_ (498.97): C, 67.40; H, 4.64; N, 11.23. Found: C, 67.48; H, 4.73; N, 11.32%.

### Ethyl 6-amino-5-(4-chlorophenyl)-2,7-diphenylpyrazolo[1,5-a]pyrimidine-3-carboxylate (3ag)

Pale yellow solid, yield: 73%, mp 238–239 °C. ^1^H NMR (500.1 MHz, DMSO-*d*_6_): *δ* 7.86 (d, *J* = 7.6 Hz, 2H, 2CH), 7.76 (d, *J* = 7.9 Hz, 2H, 2CH), 7.68 (d, *J* = 7.9 Hz, 2H, 2CH), 7.60–7.53 (m, 5H, 5CH), 7.45–7.35 (m, 3H, 3CH), 4.59 (s, 2H, NH_2_), 4.18 (q, *J* = 7.1 Hz, 2H, OCH_2_), 1.75 (t, *J* = 7.1 Hz, 3H, CH_3_). ^13^C NMR (125.1 MHz, DMSO-*d*_6_): *δ* 161.9 (C=O), 154.6, 152.5, 142.8, 136.3, 134.1 and 132.4 (6C), 131.8 (2CH), 129.2 (CH), 128.9 (2CH+C), 128.7 (2CH) 128.2 (2CH), 128.1 (2CH), 128.0 (CH), 127.1 (2CH), 126.7, 126.5 and 98.3 (3C), 58.8 (OCH_2_), 13.5 (CH_3_). Anal. Calcd for C_27_H_21_ClN_4_O_2_ (468.94): C, 69.16; H, 4.51; N, 11.95. Found: C, 69.08; H, 4.43; N, 12.08%.

### Ethyl 6-amino-5,7-bis(4-chlorophenyl)-2-phenylpyrazolo[1,5-a]pyrimidine-3-carboxylate (3ah)

Pale yellow solid, yield: 92%, mp 245–246 °C. IR (KBr) (*ν*_max_/cm^–1^): 3449 and 3357 (NH_2_), 1732 (C=O). ^1^H NMR (500.1 MHz, CDCl_3_): *δ* 7.93 (d, *J* = 8.5 Hz, 2H, 2CH), 7.79–7.70 (m, 4H, 4CH), 7.61 (d, *J* = 8.5 Hz, 2H, 2CH), 7.55 (d, *J* = 8.4 Hz, 2H, 2CH), 7.45–7.37 (m, 3H, 3CH), 4.34 (q, *J* = 7.1 Hz, 2H, OCH_2_), 3.74 (s, 2H, NH_2_),1.30 (t, *J* = 7.1 Hz, 3H, CH_3_). ^13^C NMR (125.1 MHz, CDCl_3_): *δ* 163.3 (C=O), 157.1, 151.6, 145.0, 136.7, 136.4, 135.1 and 132.9 (7C), 131.6 (2CH), 130.4 (2CH), 129.9 (2CH), 129.8 (2CH), 129.2 (2CH), 128.8 (CH), 128.3 and 128.2 (2C), 127.7 (2CH), 127.6 and 100.2 (2C), 60.1 (OCH_2_), 14.2 (CH_3_). EI-MS, *m/z* (%): 506 (M^+ 37^Cl_2_, 69), 504 (M^+ 37^Cl^35^Cl, 69), 502 (M^+ 35^Cl_2_, 96), 457 (38), 430 (67), 355 (19), 265 (9), 237 (14), 189 (10), 155 (41), 138 (24), 123 (14), 114 (14), 105 (54), 89 (29), 77 (100), 63 (7), 51 (19). Anal. Calcd for C_27_H_20_Cl_2_N_4_O_2_ (503.39): C, 64.42; H, 4.00; N, 11.13. Found: C, 64.39; H, 4.11; N, 11.08%.

### Ethyl 6-amino-5-(4-chlorophenyl)-2-phenyl-7-(thiophen-2-yl)pyrazolo[1,5-a]pyrimidine-3-carboxylate (3ai)

Pale yellow solid, yield: 68%, mp 192–193 °C. ^1^H NMR (500.1 MHz, CDCl_3_): *δ* 8.49 (dd, *J* = 0.9, 3.9 Hz, 1H, CH), 8.25 (d, *J* = 8.7 Hz, 2H, 2CH), 8.00 (dd, *J* = 2.2, 7.9 Hz, 2H, 2CH), 7.80 (dd, *J* = 1.0, 5.0 Hz, 1H, CH), 7.65–7.50 (m, 5H, 5CH), 7.36–7.30 (m, 1H, CH), 4.45 (q, *J* = 7.0 Hz, 2H, OCH_2_), 3.78 (s, 2H, NH_2_), 1.42 (t, *J* = 7.0 Hz, 3H, CH_3_). ^13^C NMR (125.1 MHz, CDCl_3_): *δ* 163.4 (C=O), 156.6, 150.3, 140.5, 137.2, 135.5, 133.38, 133.36 and 132.6 (8C), 132.2 (CH), 131.0 (C), 130.0 (2CH), 129.3 (CH), 129.2 (2CH), 128.8 (2CH), 128.7 (CH), 127.93 (2CH), 127.86 (CH), 102.0 (C), 60.4 (OCH_2_), 14.3 (CH_3_). Anal. Calcd for C_25_H_19_ClN_4_O_2_S (474.97): C, 63.22; H, 4.03; N, 11.80. Found: C, 63.31; H, 4.12; N, 11.86%.

#### *α*-Glucosidase inhibition assay

*α*-Glucosidase enzyme (EC3.2.1.20, Saccharomyces cerevisiae, 20 U/mg) and substrate (p-nitrophenyl glucopyranoside) were purchased from Sigma-Aldrich. Enzymewas prepared in potassium phosphate buffer (pH 6.8, 50 mM), and 6-amino-pyrazolo[1,5-a]pyrimidine derivatives **3** were dissolved in DMSO (10% final concentration). The various concentrations of compounds **3** (20 mL), enzyme solution (20 mL) and potassium phosphate buffer (135 mL), were added in the 96-well plate and incubated at 37 °C for 10 min. Then, the substrate (25 mL, 4 mM) was added to the mentioned mixture and allowed to incubate at 37 °C for 20 min. Finally, the change in absorbance was measured at 405 nm by using spectrophotometer (Gen5, Power wave xs2, BioTek, America). DMSO (10% final concentration) and acarbose were used respectively as control and standard drug. The percentage of enzyme inhibitionwas calculated and IC_50_ values were obtained from non-linear regression curve using the Logit method^84^.

#### Kinetic studies

The kinetic analysis were carried out to determine inhibition mode of the most potent compounds (**3d** and **3af**). The 20 mL of enzyme solution (1 U/mL) was incubated with different concentrations (0, 5, 10, and 15 µM) of selected compounds for 15 min at 30 °C. The reaction was then started by adding different concentrations of substrate (*p*-nitrophenyl glucopyranoside, 1-10 mM), and change in absorbance was measured for 20 min at 405 nm by using spectrophotometer (Gen5, Power wave xs2, BioTek, America).

#### Molecular docking studies

Autodock 4.2.6 program was used to determine the probable binding conformations of the compounds **3d** and **3af** over the *α*-glucosidase active site. AutoDockTools 1.5.2 (ADT) was utilized to prepare the input files. The 3D structure of the most active compounds were drawn **3d** and **3af** using MarvineSketch 5.8.3, 2012, ChemAxon (http://www.chemaxon.com) and converted to pdbqt coordinate using Auto dockTools^85^. In AUTOGRID for each atom type in the ligand, maps were calculated with 0.375 Å spacing between grid points, and a grid box of 40 × 40 × 40 Å was created at the center of 12.5825, −7.8955, 12.519 in each dimension to determine the ligand- enzyme interactions. Rigid ligand dockings were accomplished for the selected compounds. Of the three different search algorithms suggested by AutoDock 4.2.6, the Lamarckian genetic algorithm (LGA) consisting of 50 runs, 25 × 10^6^ energy evaluations, and 27,000 generations was carried out^86^. Other docking parameters were set to default. The best interaction of the selected compounds were considered for analyzing and the results were illustrated using Discovery Studio 4.5 Client.

## Supplememtary information

Supplememtary information.
