# Supplementary material for: An efficient and targeted synthetic approach towards new highly substituted 6-amino-pyrazolo[1,5-a]pyrimidines with α-glucosidase inhibitory activity
Source: Sci Rep. 2020 Feb 13;10:2595. doi: 10.1038/s41598-020-59079-z (PMC7018746; doi:10.1038/s41598-020-59079-z)

An efficient, targeted synthetic approach towards new highly substituted 6-amino-pyrazolo[1,5-*a*]pyrimidines with *α*-glucosidase inhibitory activity

Fariba Peytam [a,b], Mehdi Adib [a,*], Reihaneh Shourgeshty [a,b], Loghman Firoozpour [b], Mahmoud Rahmanian-Jazi [b], Mehdi Jahani [a], Setareh Moghimi [b], Kouros Divsalar [c], Mohammad Ali Faramarzi [d], Somaye Mojtabavi [d], Fatemeh Safari [e], Mohammad Mahdavi [f], Alireza Foroumadi [b,c,*]

[a] School of Chemistry, College of Science, University of Tehran, Tehran, Iran

[b] Department of Medicinal Chemistry, Faculty of Pharmacy and The Institute of Pharmaceutical Sciences (TIPS), Tehran University of Medical Sciences, Tehran, Iran

[c] Neuroscience Research Center, Institute of Neuropharmacology, Kerman University of Medical Sciences, Kerman, Iran

[d] Department of Pharmaceutical Biotechnology, Faculty of Pharmacy and Biotechnology Research Center, Tehran University of Medical Sciences, Tehran, Iran

e Department of Biology, Faculty of Science, University of Guilan, Rasht, Iran

[f] Endocrinology and Metabolism Research Center, Endocrinology and Metabolism Clinical Sciences Institute, Tehran University of Medical Sciences, Tehran, Iran

^{1}H NMR spectrum of 6-amino-7-(4-chlorophenyl)-N,5-diphenyl-2-(phenylamino)pyrazolo[1,5-a]pyrimidine-3-carboxamide (**3a**)

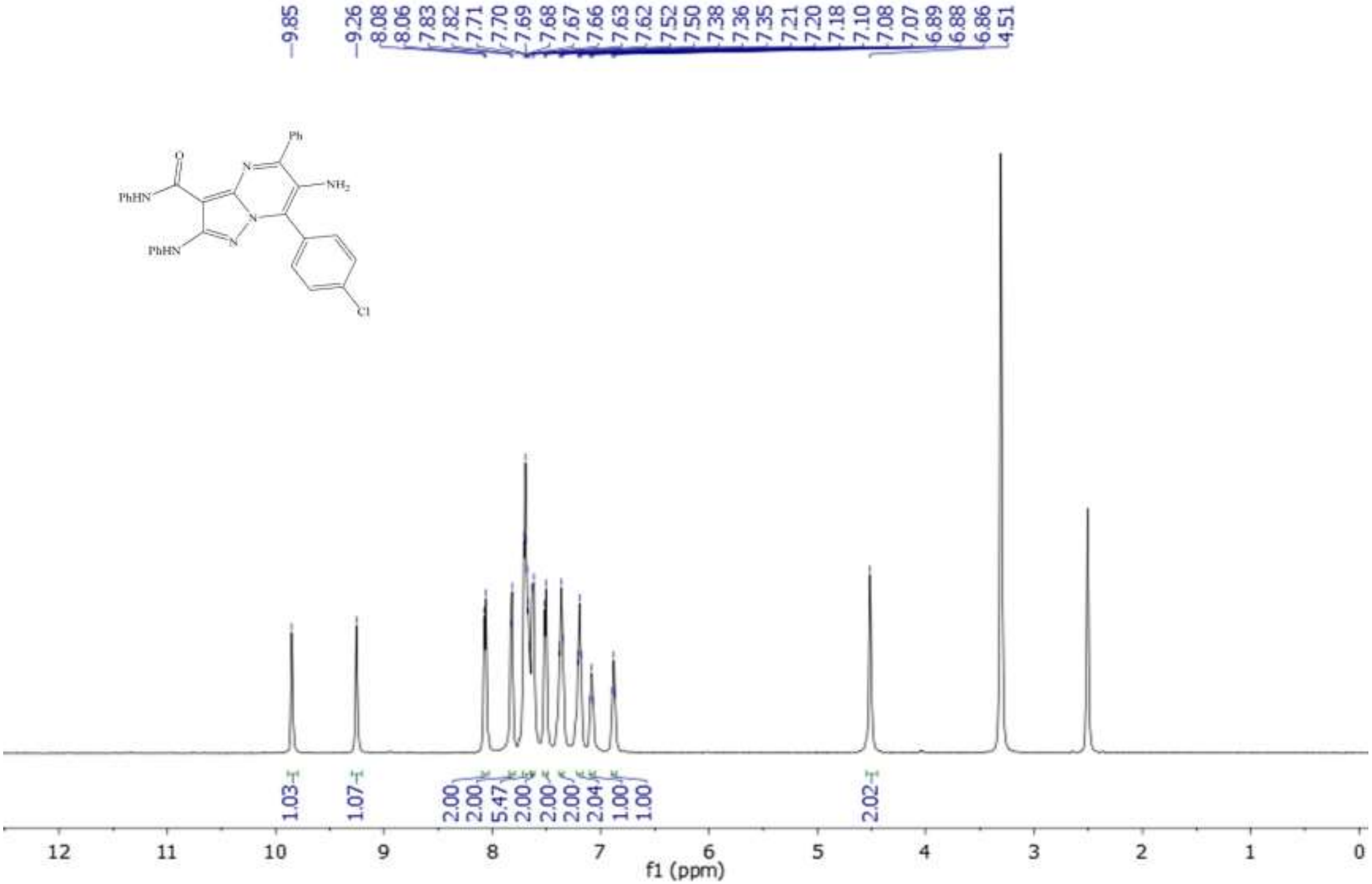

^{13}C NMR spectrum of 6-amino-7-(4-chlorophenyl)-N,5-diphenyl-2-(phenylamino)pyrazolo[1,5-a]pyrimidine-3-carboxamide (**3a**)

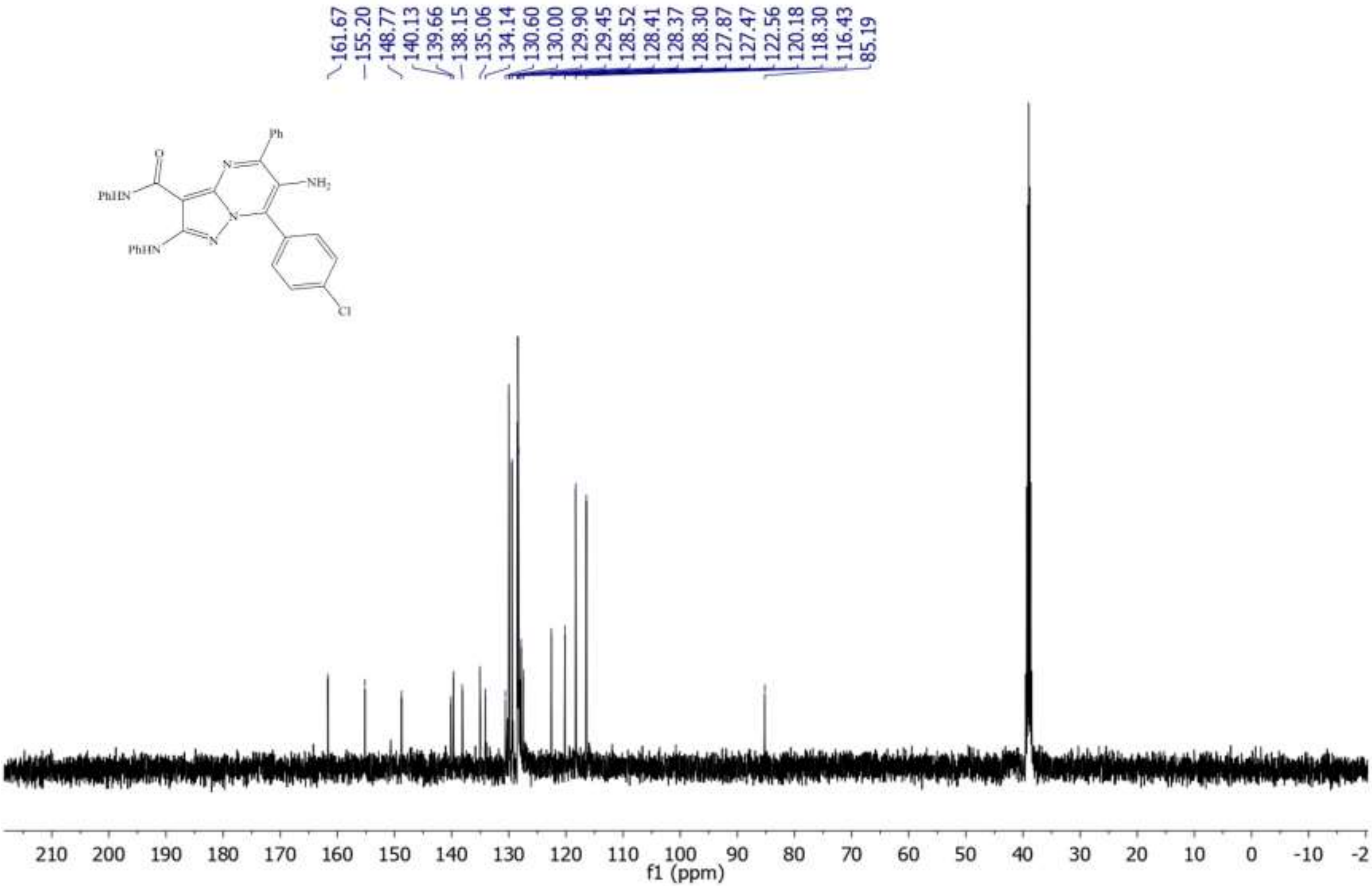

^{1}H NMR spectrum of 6-amino-7-(4-bromophenyl)-N,5-diphenyl-2-(phenylamino)pyrazolo[1,5-a]pyrimidine-3-carboxamide (**3b**)

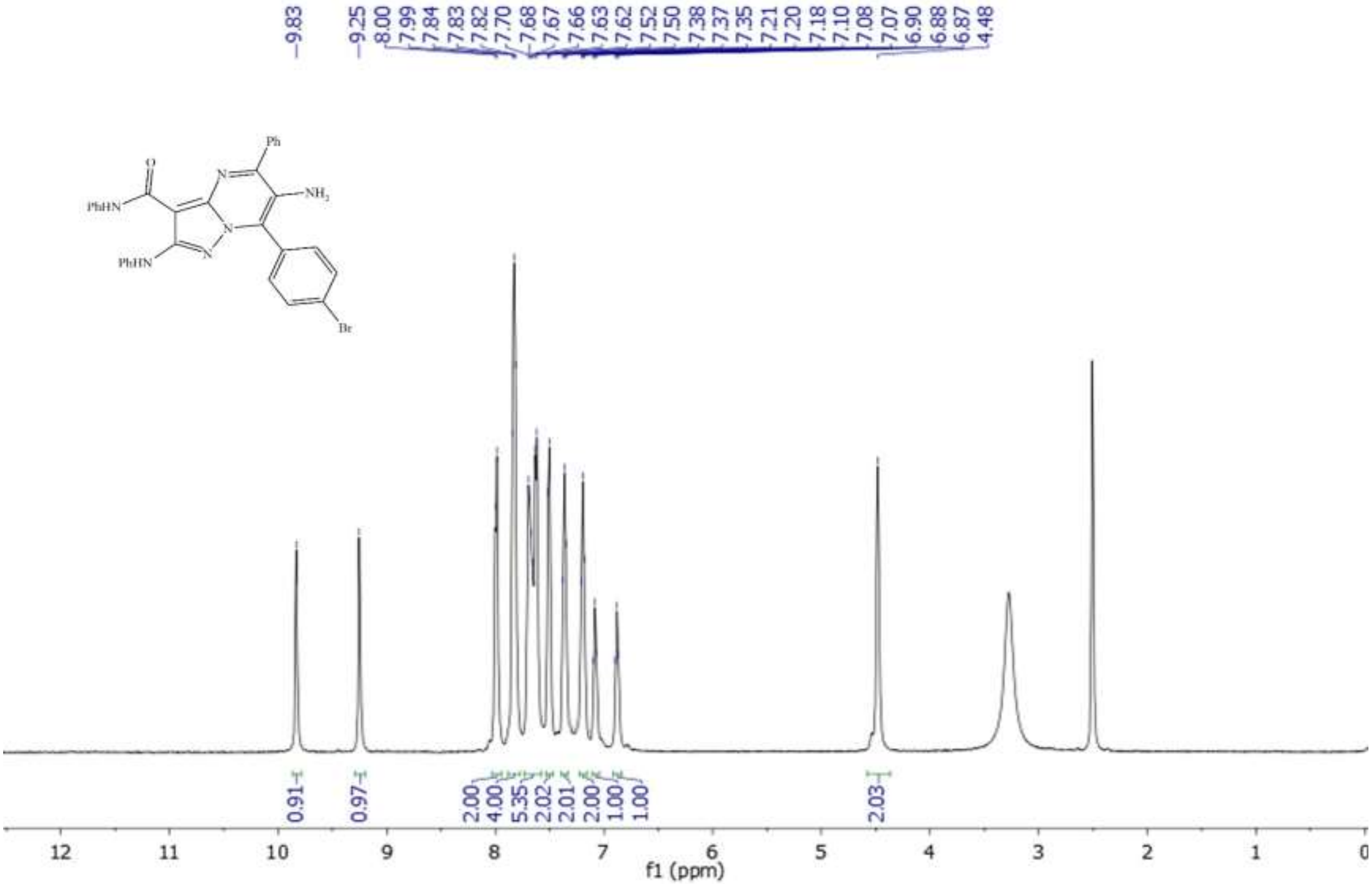

^{13}C NMR spectrum of 6-amino-7-(4-bromophenyl)-N,5-diphenyl-2-(phenylamino)pyrazolo[1,5-a]pyrimidine-3-carboxamide (**3b**)

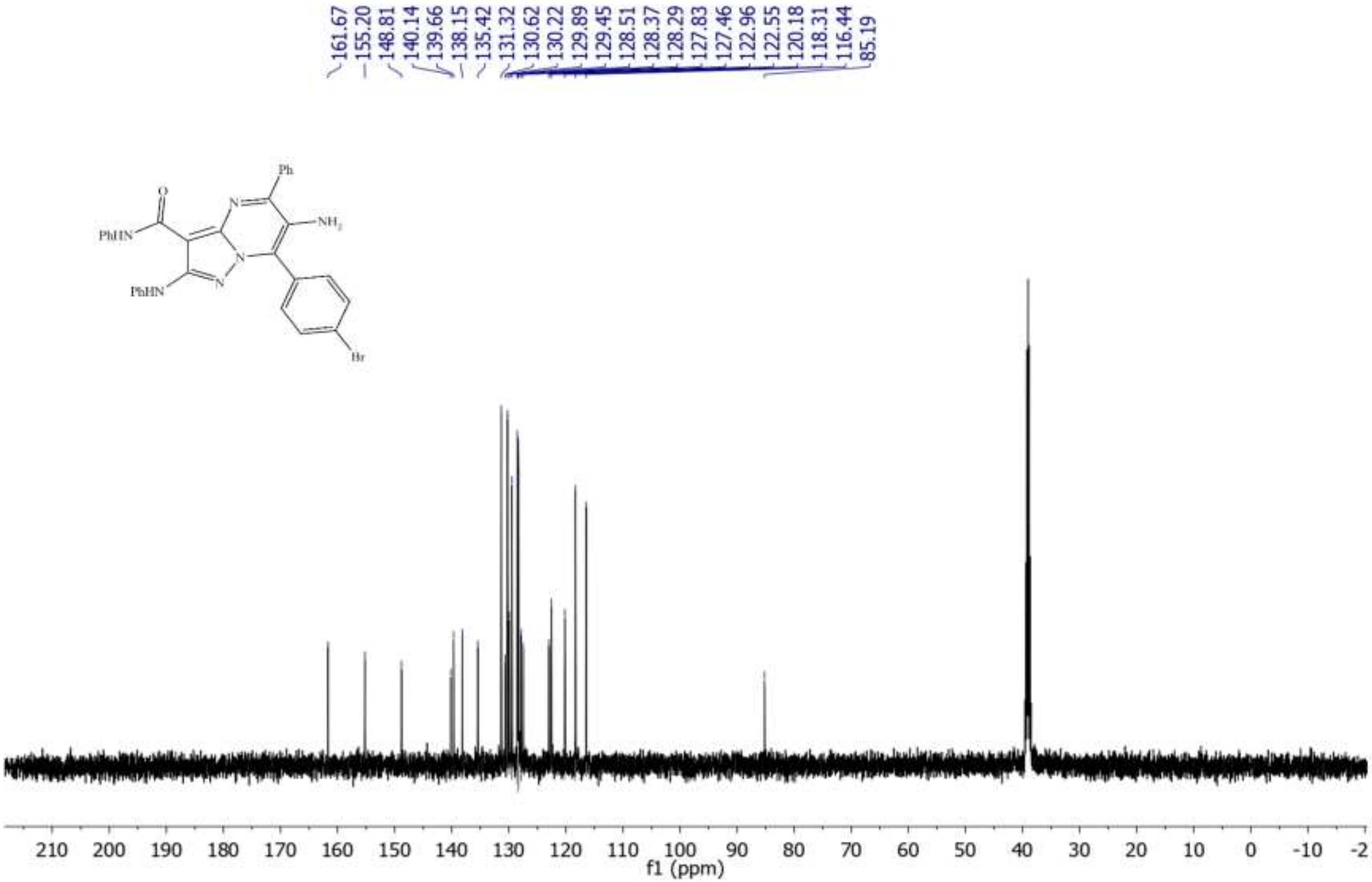

^{1}H NMR spectrum of 6-amino-7-(4-chlorophenyl)-N-phenyl-2-(phenylamino)-5-p-tolylpyrazolo[1,5-a]pyrimidine-3-carboxamide (**3c**)

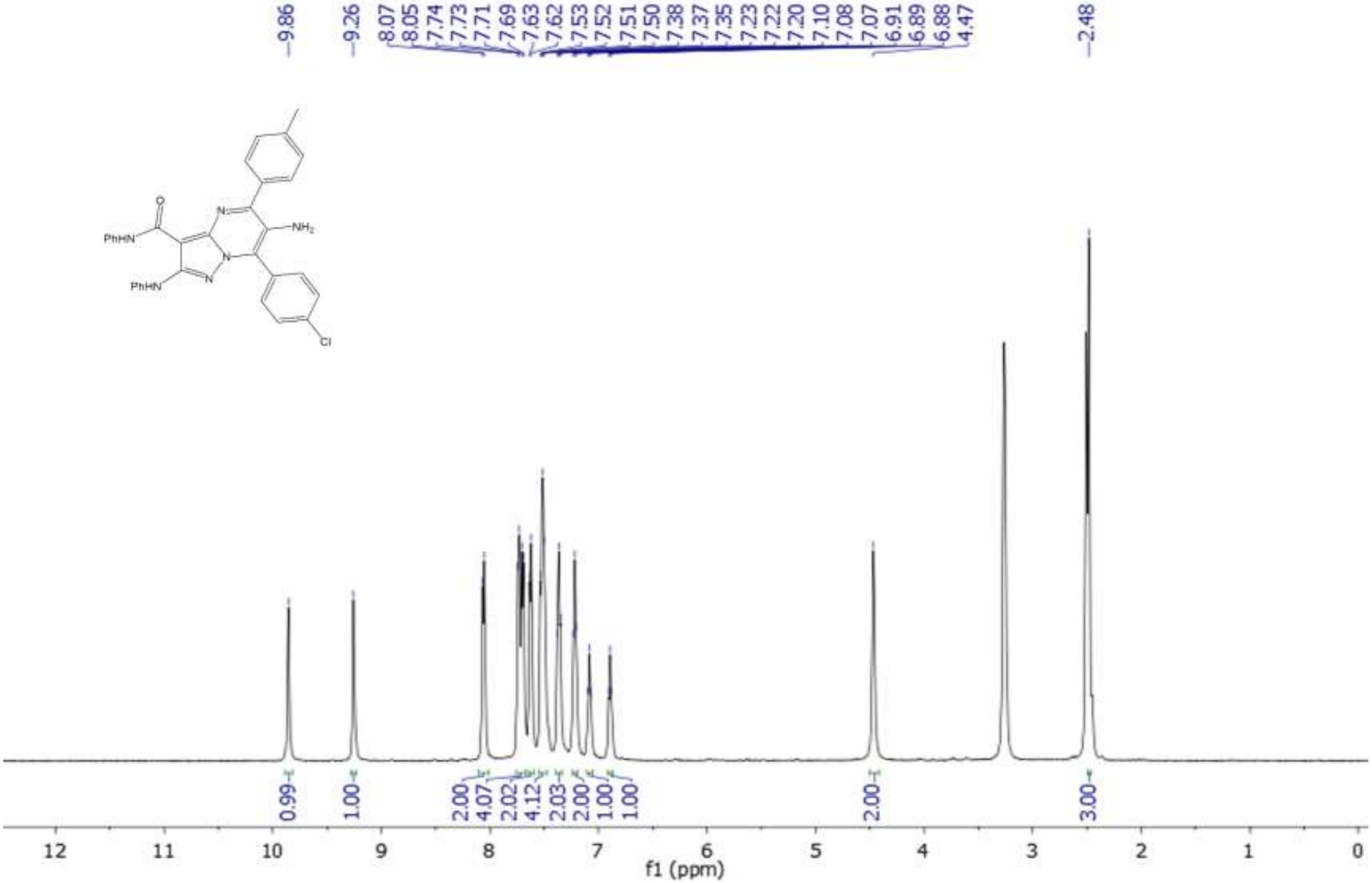

^{13}C NMR spectrum of 6-amino-7-(4-chlorophenyl)-N-phenyl-2-(phenylamino)-5-p-tolylpyrazolo[1,5-a]pyrimidine-3-carboxamide (**3c**)

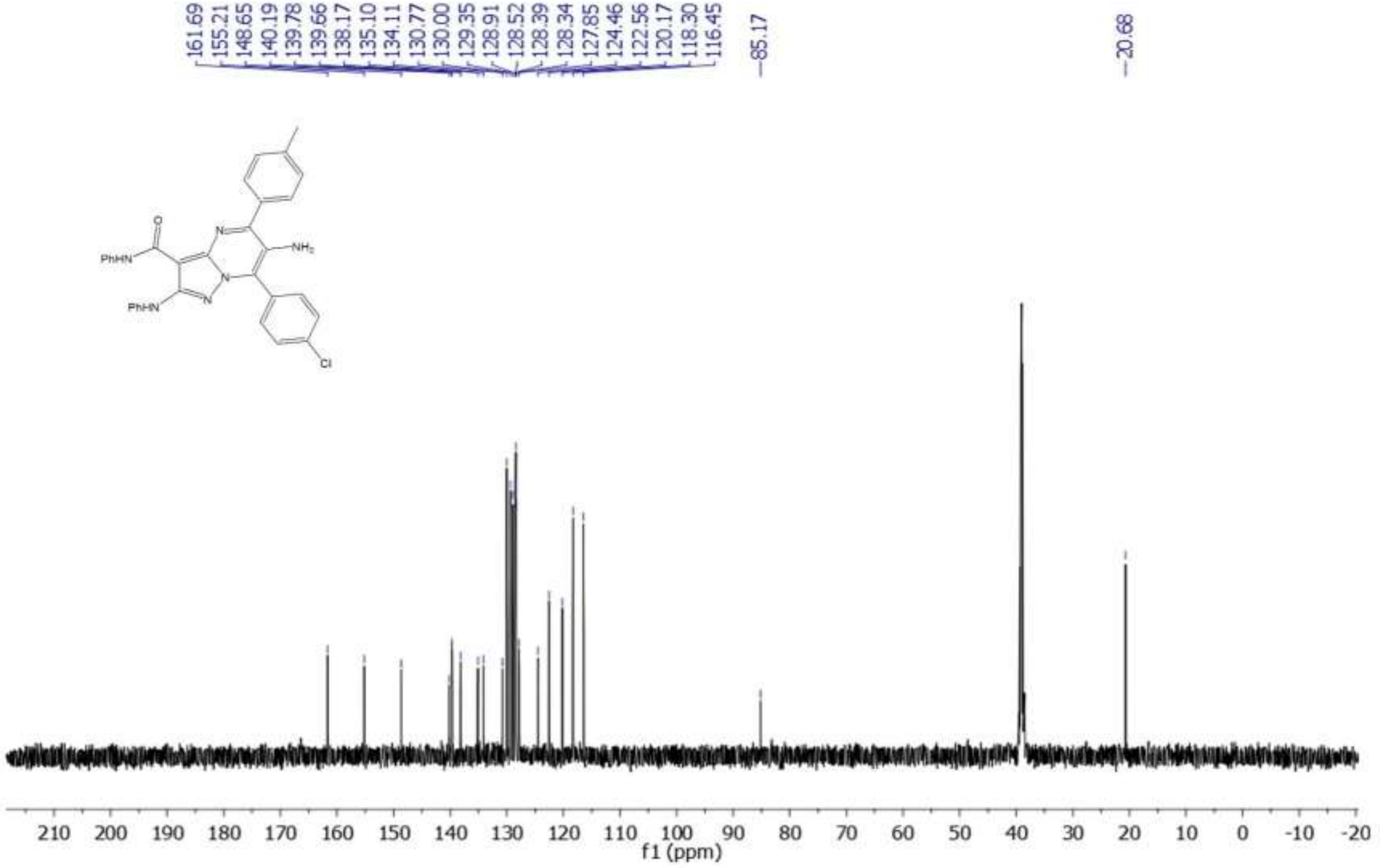

^{1}H NMR spectrum of 6-amino-7-(4-bromophenyl)-N-phenyl-2-(phenylamino)-5-p-tolylpyrazolo[1,5-a]pyrimidine-3-carboxamide (**3d**)

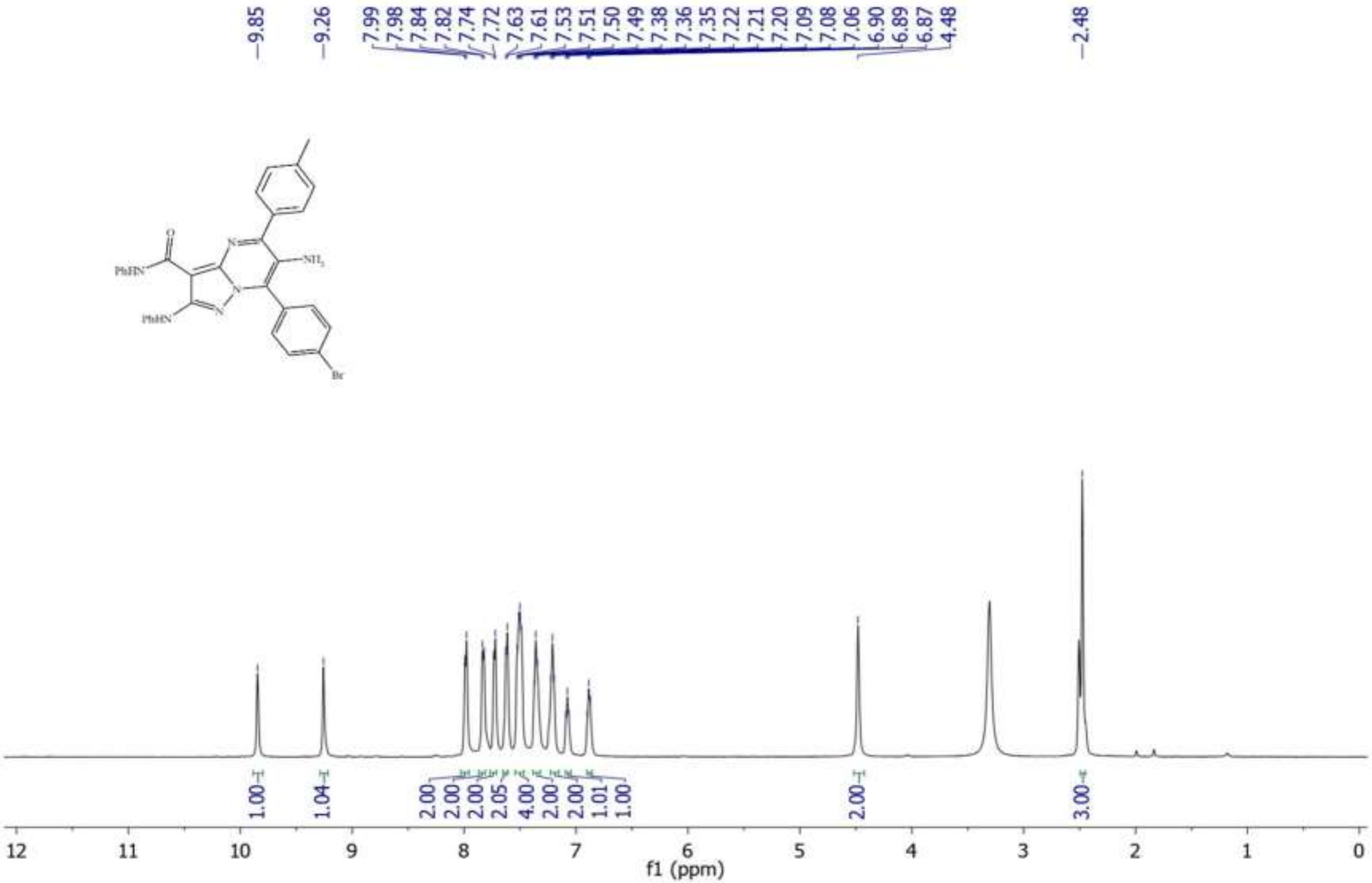

^{13}C NMR spectrum of 6-amino-7-(4-bromophenyl)-N-phenyl-2-(phenylamino)-5-p-tolylpyrazolo[1,5-a]pyrimidine-3-carboxamide(**3d**)

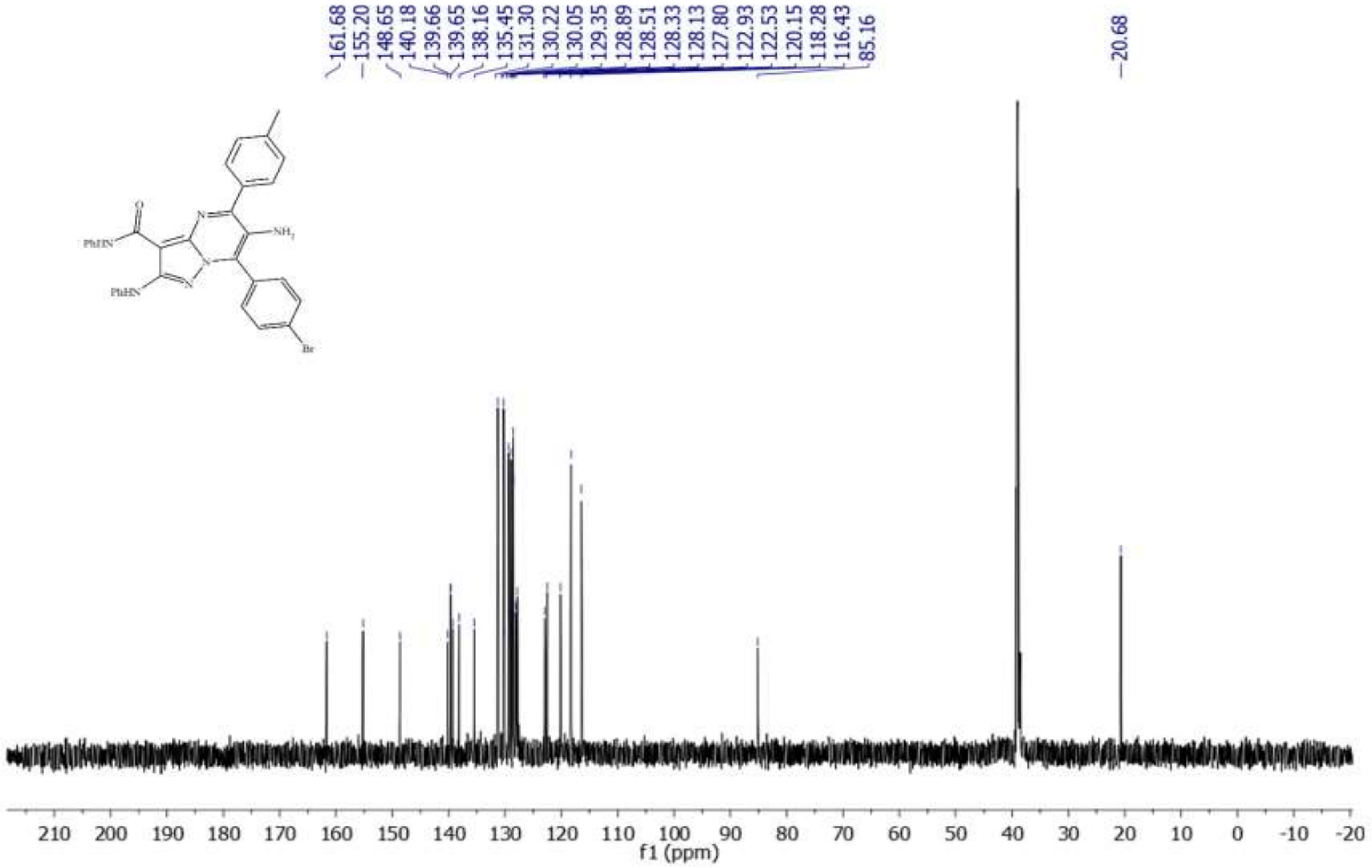

^{1}H NMR spectrum of 6-amino-5-(4-methoxyphenyl)-N,7-diphenyl-2-(phenylamino)pyrazolo[1,5-a]pyrimidine-3-carboxamide (**3e**)

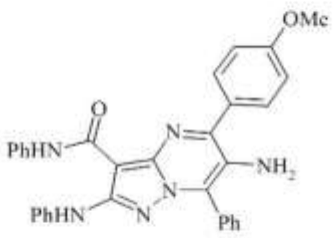

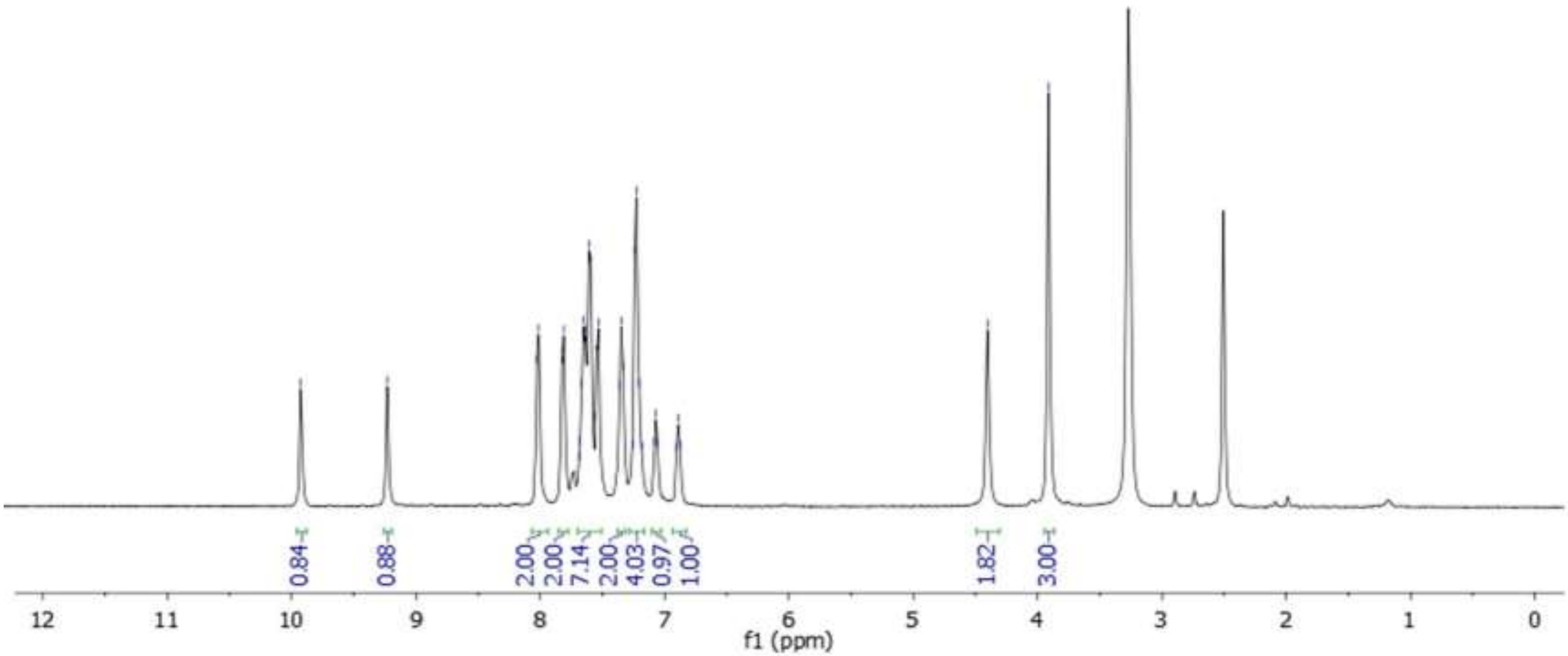

^{13}C NMR spectrum of 6-amino-5-(4-methoxyphenyl)-N,7-diphenyl-2-(phenylamino)pyrazolo[1,5-a]pyrimidine-3-carboxamide (**3e**)

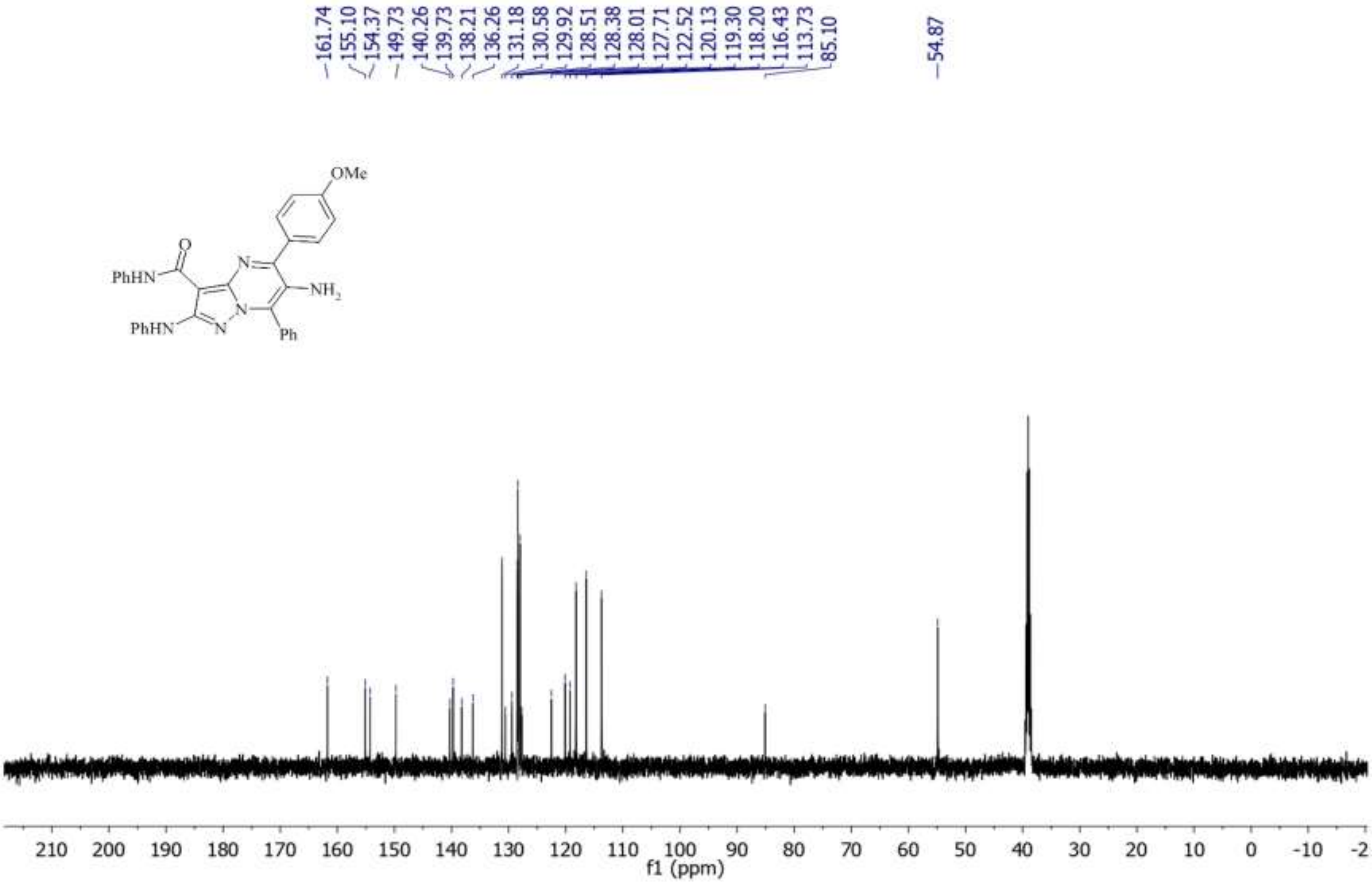

^{1}H NMR spectrum of 6-amino-7-(4-chlorophenyl)-5-(4-methoxyphenyl)-N-phenyl-2-(phenylamino)pyrazolo[1,5-a]pyrimidine-3-carboxamide (**3f**)

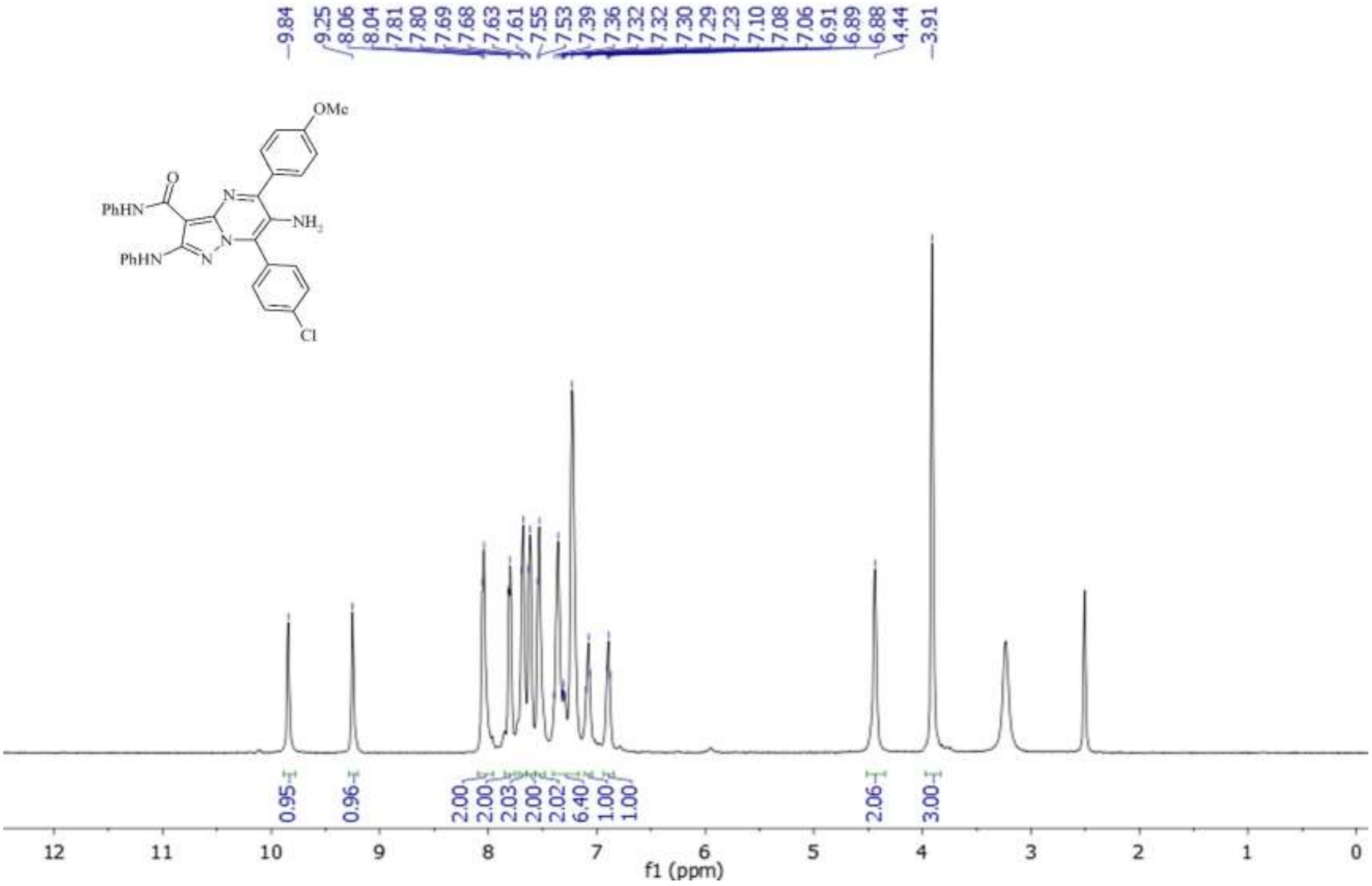

^{13}C NMR spectrum of 6-amino-7-(4-chlorophenyl)-5-(4-methoxyphenyl)-N-phenyl-2-(phenylamino)pyrazolo[1,5-a]pyrimidine-3-carboxamide (**3f**)

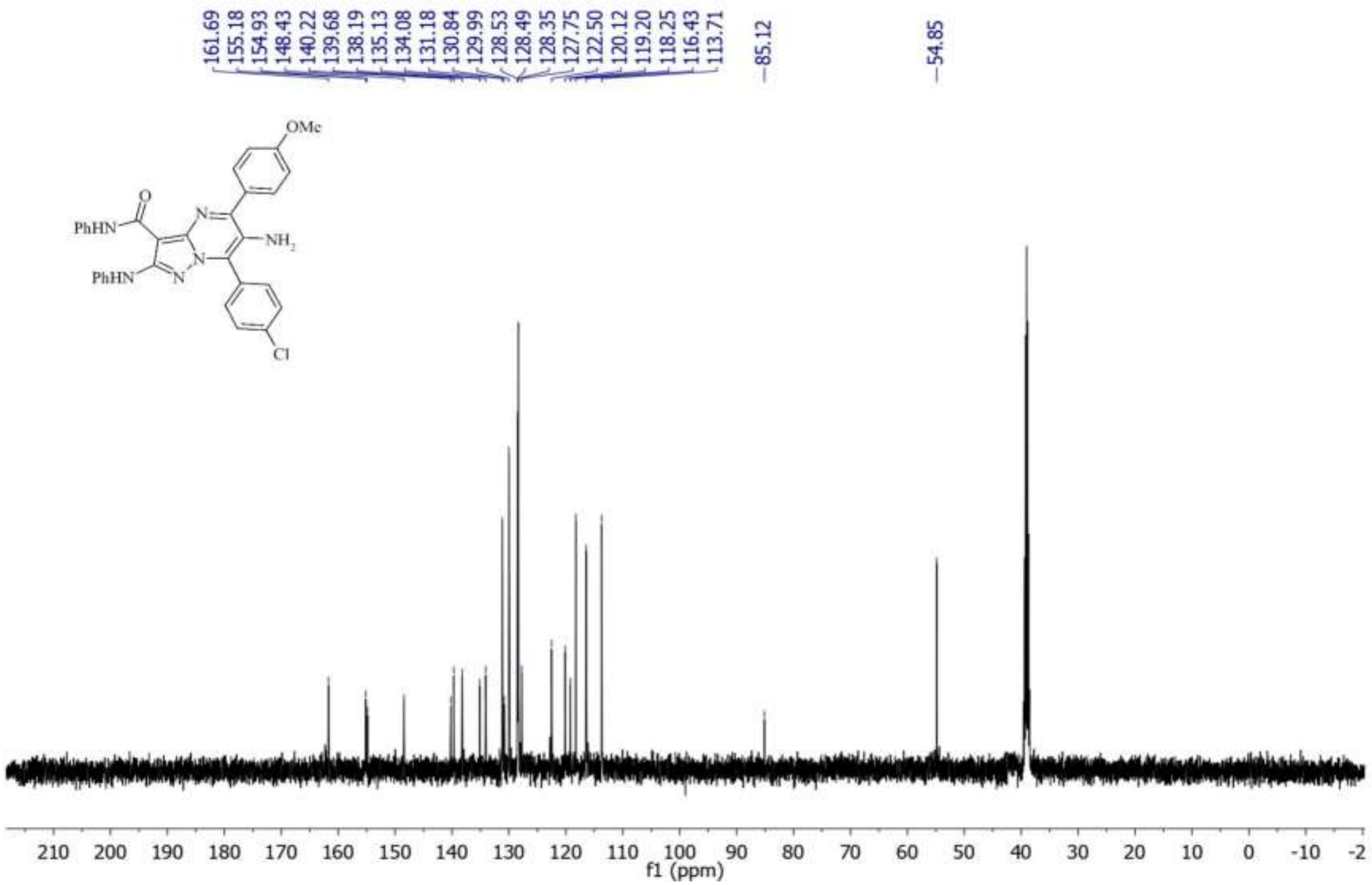

^{1}H NMR spectrum of 6-amino-5-(4-chlorophenyl)-N,7-diphenyl-2-(phenylamino)pyrazolo[1,5-a]pyrimidine-3-carboxamide (**3g**)

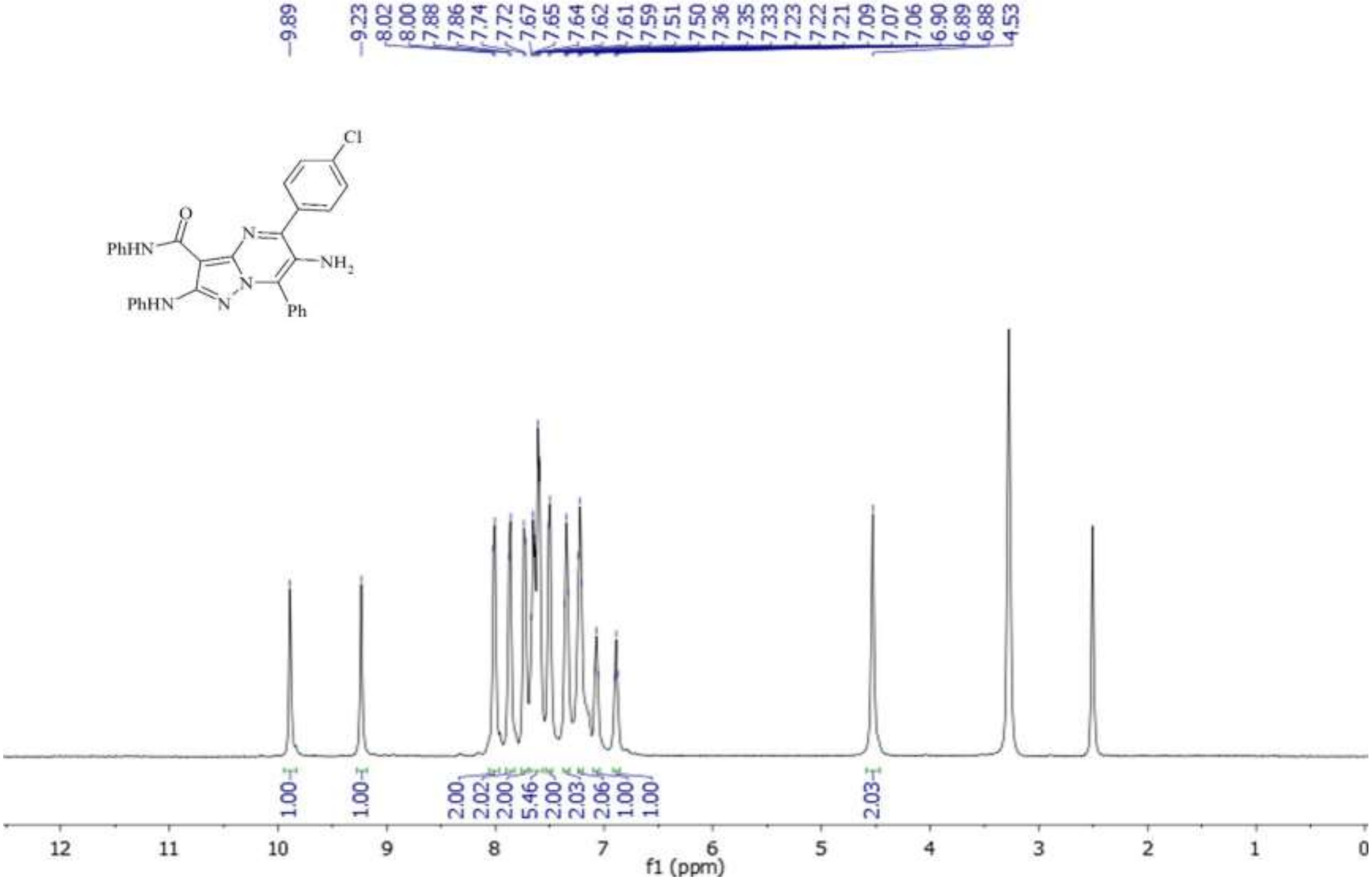

^{13}C NMR spectrum of 6-amino-5-(4-chlorophenyl)-N,7-diphenyl-2-(phenylamino)pyrazolo[1,5-a]pyrimidine-3-carboxamide (**3g**)

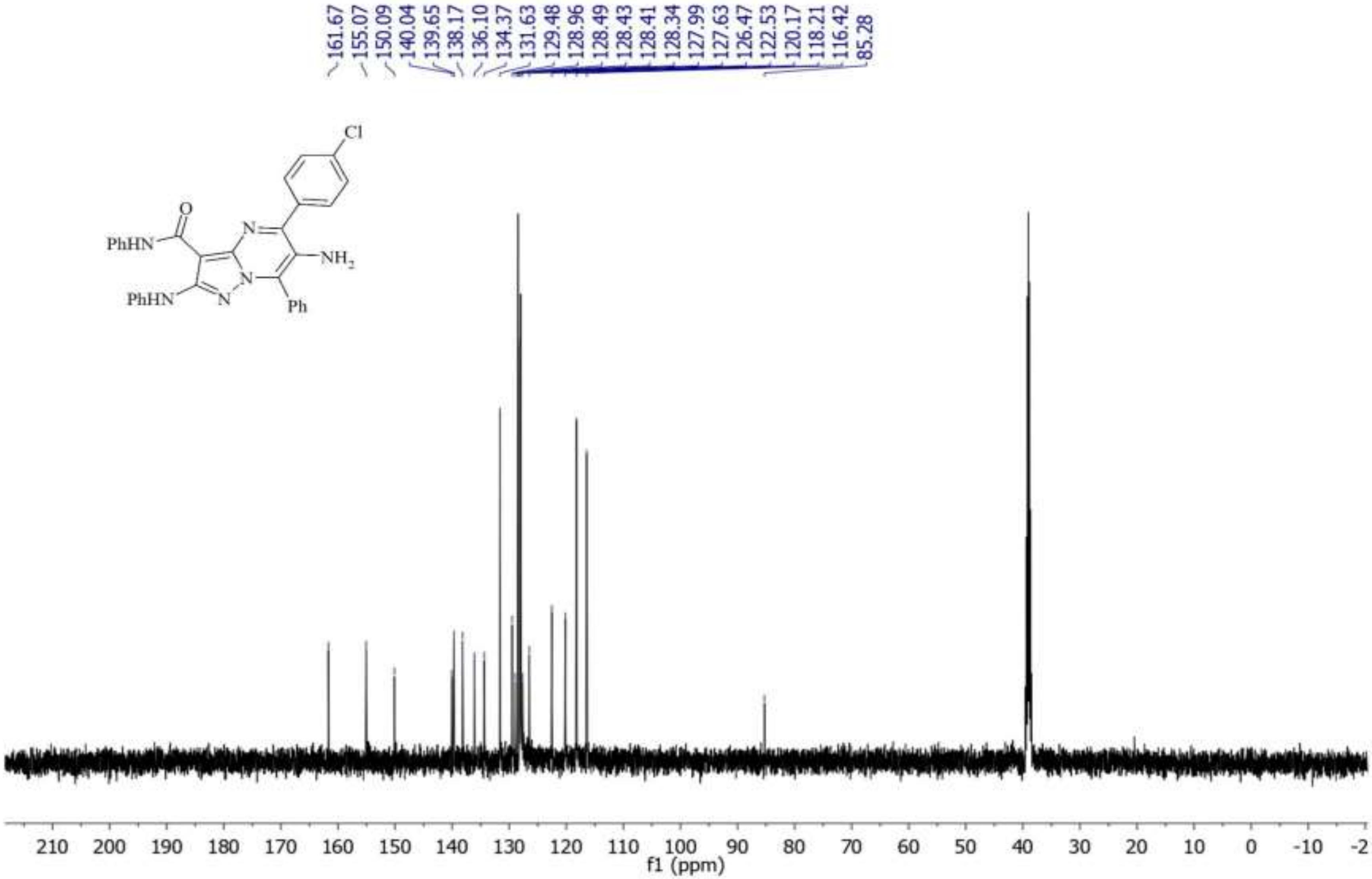

^{1}H NMR spectrum of 6-amino-5,7-bis(4-chlorophenyl)-N-phenyl-2-(phenylamino)pyrazolo[1,5-a]pyrimidine-3-carboxamide (**3h**)

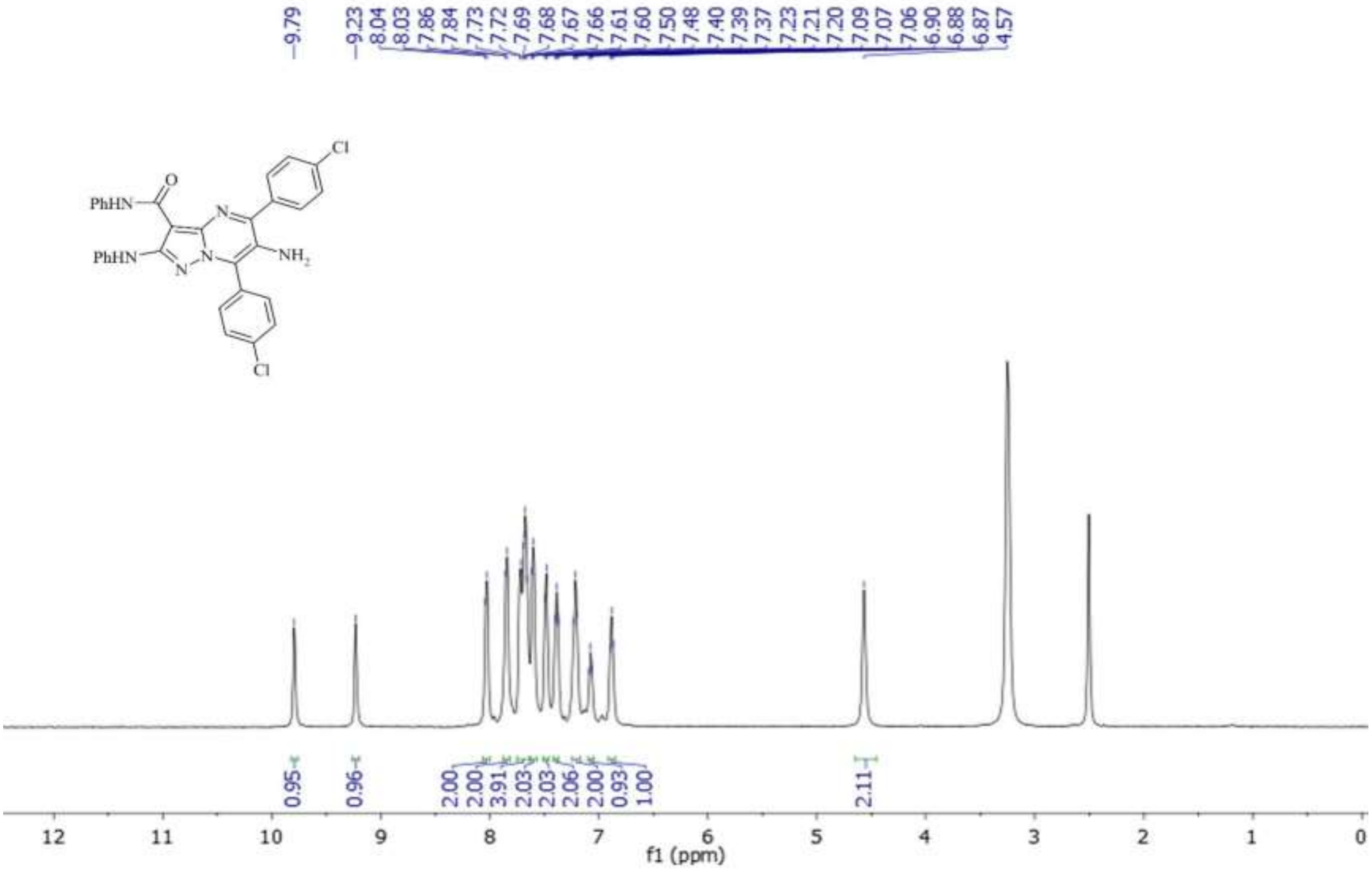

^{13}C NMR spectrum of 6-amino-5,7-bis(4-chlorophenyl)-N-phenyl-2-(phenylamino)pyrazolo[1,5-a]pyrimidine-3-carboxamide (**3h**)

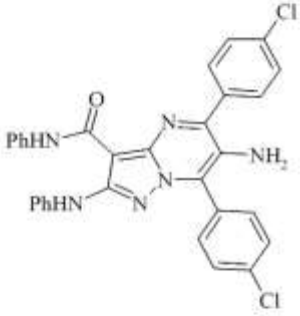

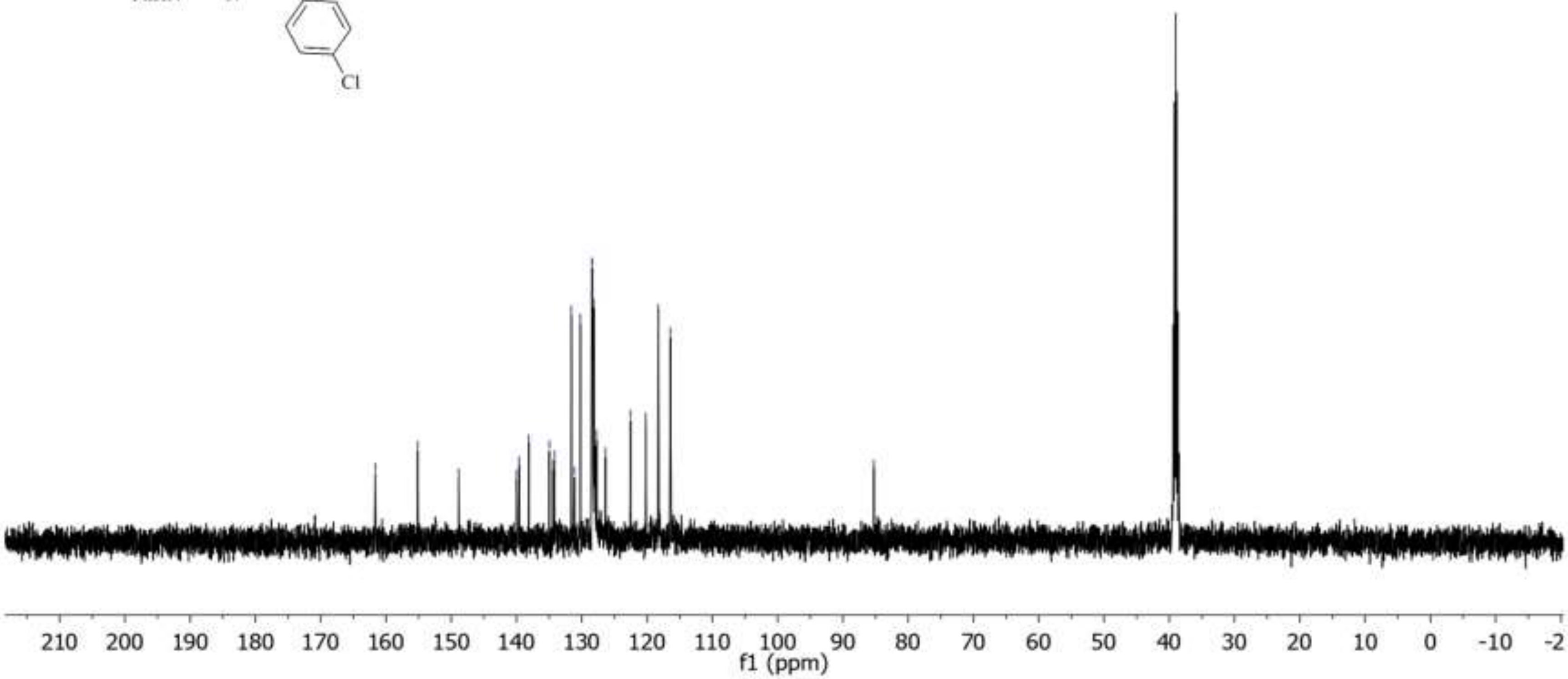

^{1}H NMR spectrum of 6-amino-5-(4-chlorophenyl)-N-phenyl-2-(phenylamino)-7-(thiophen-2-yl)pyrazolo[1,5-a]pyrimidine-3-carboxamide (**3i**)

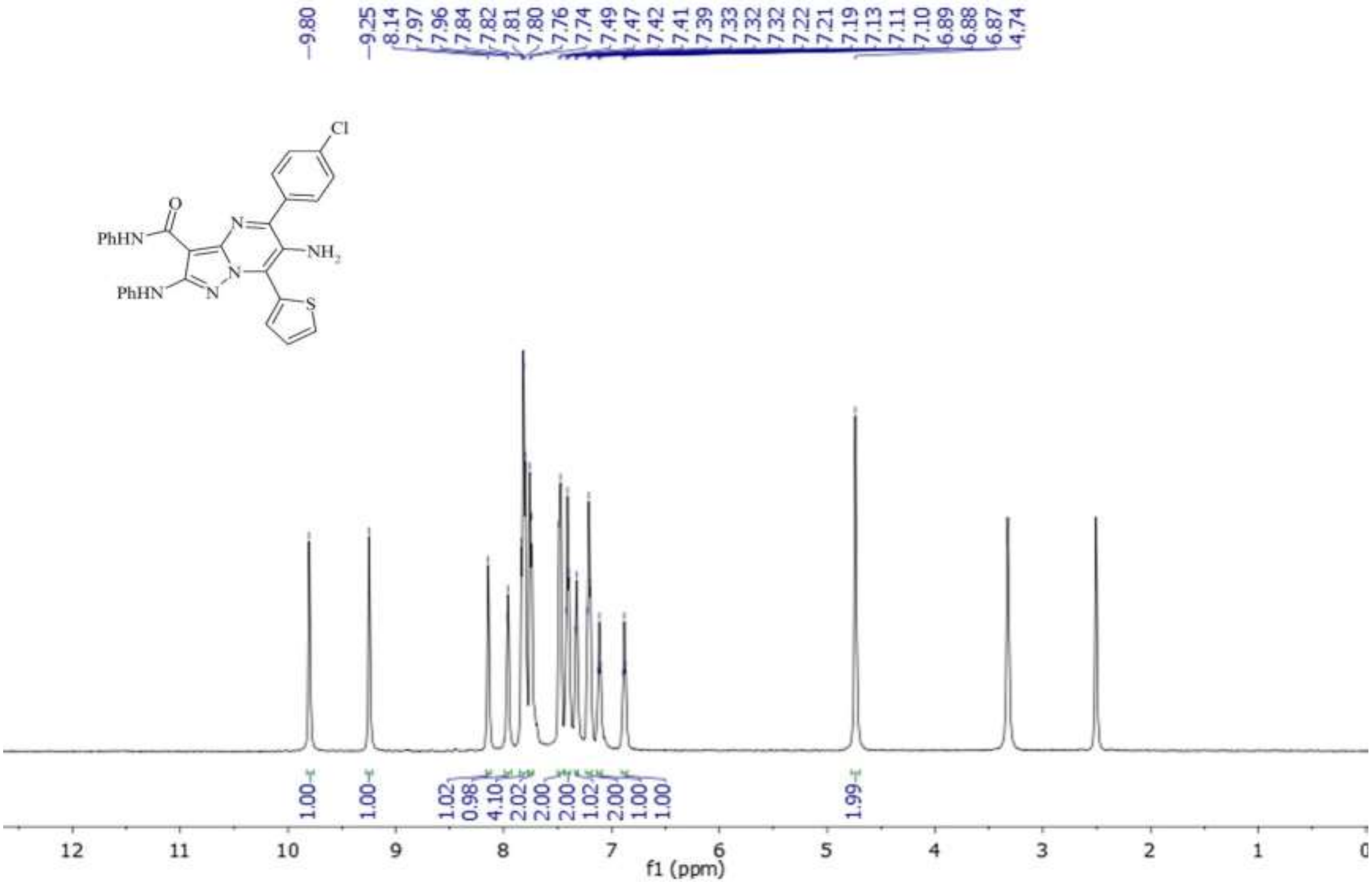

^{13}C NMR spectrum of 6-amino-5-(4-chlorophenyl)-N-phenyl-2-(phenylamino)-7-(thiophen-2-yl)pyrazolo[1,5-a]pyrimidine-3-carboxamide (**3i**)

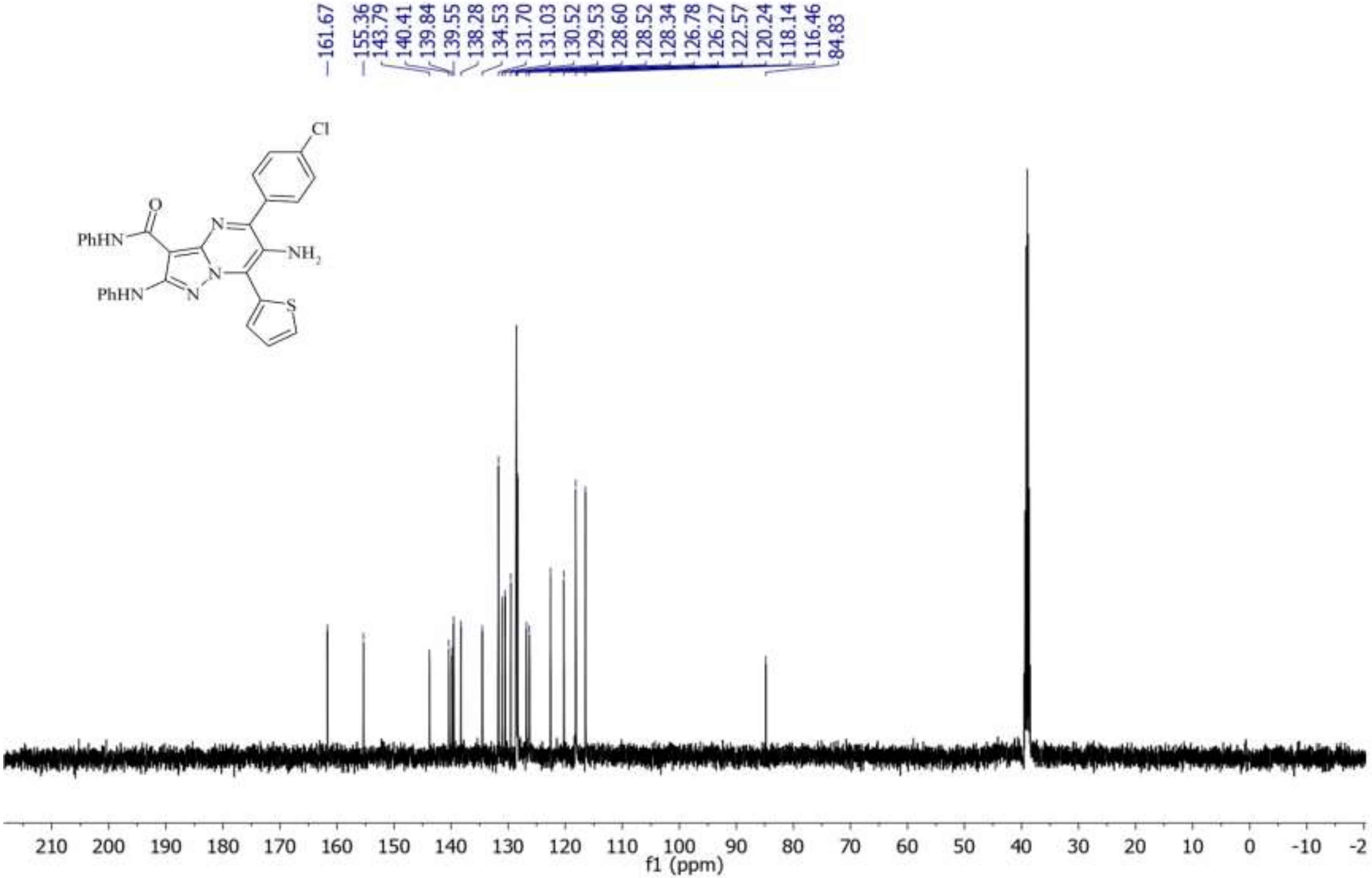

^{1}H NMR spectrum of 6-amino-7-(4-chlorophenyl)-N-(4-methoxyphenyl)-5-phenyl-2-(phenylamino)pyrazolo[1,5-a]pyrimidine-3-carboxamide (**3j**)

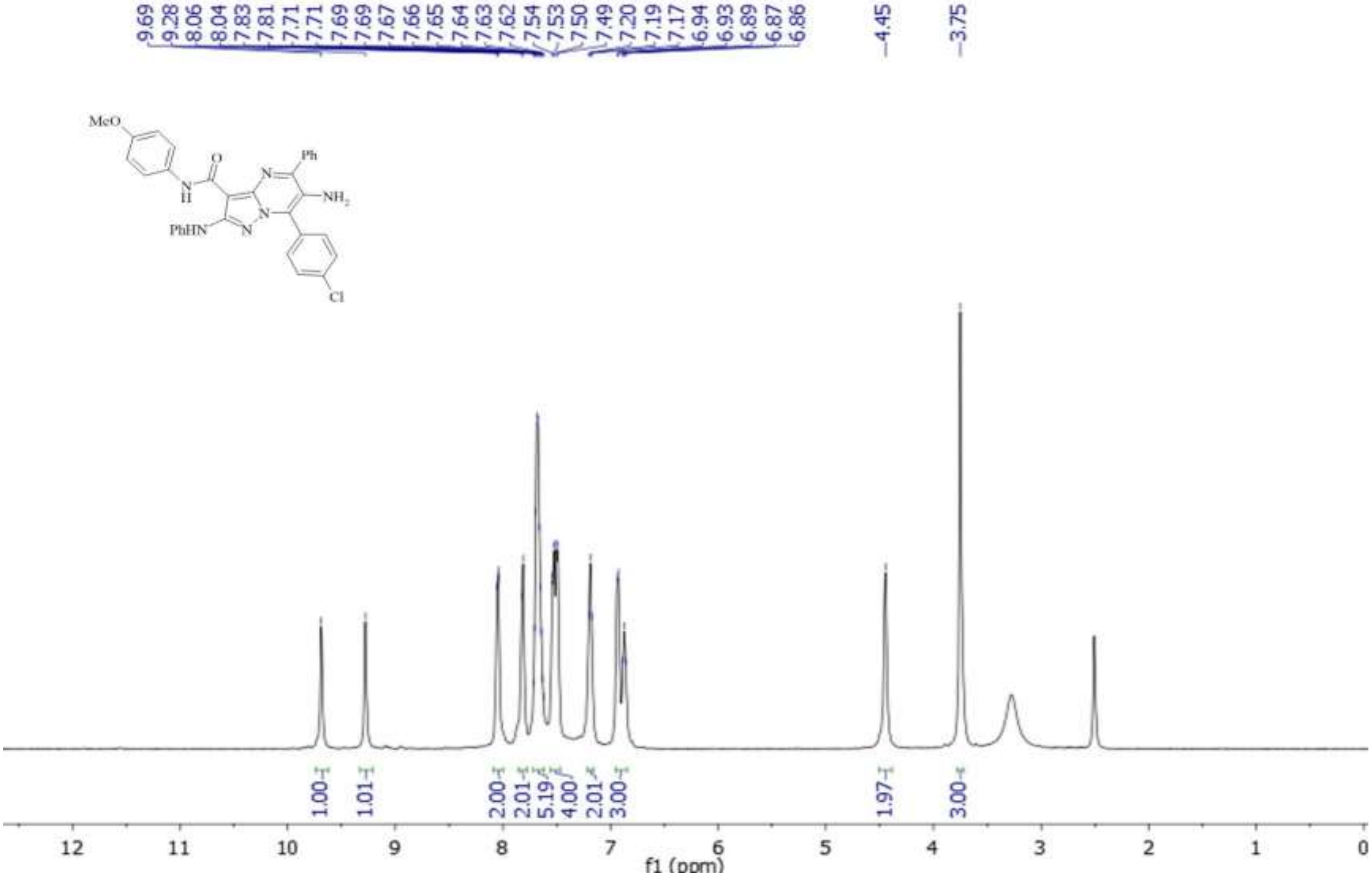

^{13}C NMR spectrum of 6-amino-7-(4-chlorophenyl)-N-(4-methoxyphenyl)-5-phenyl-2-(phenylamino)pyrazolo[1,5-a]pyrimidine-3-carboxamide (**3j**)

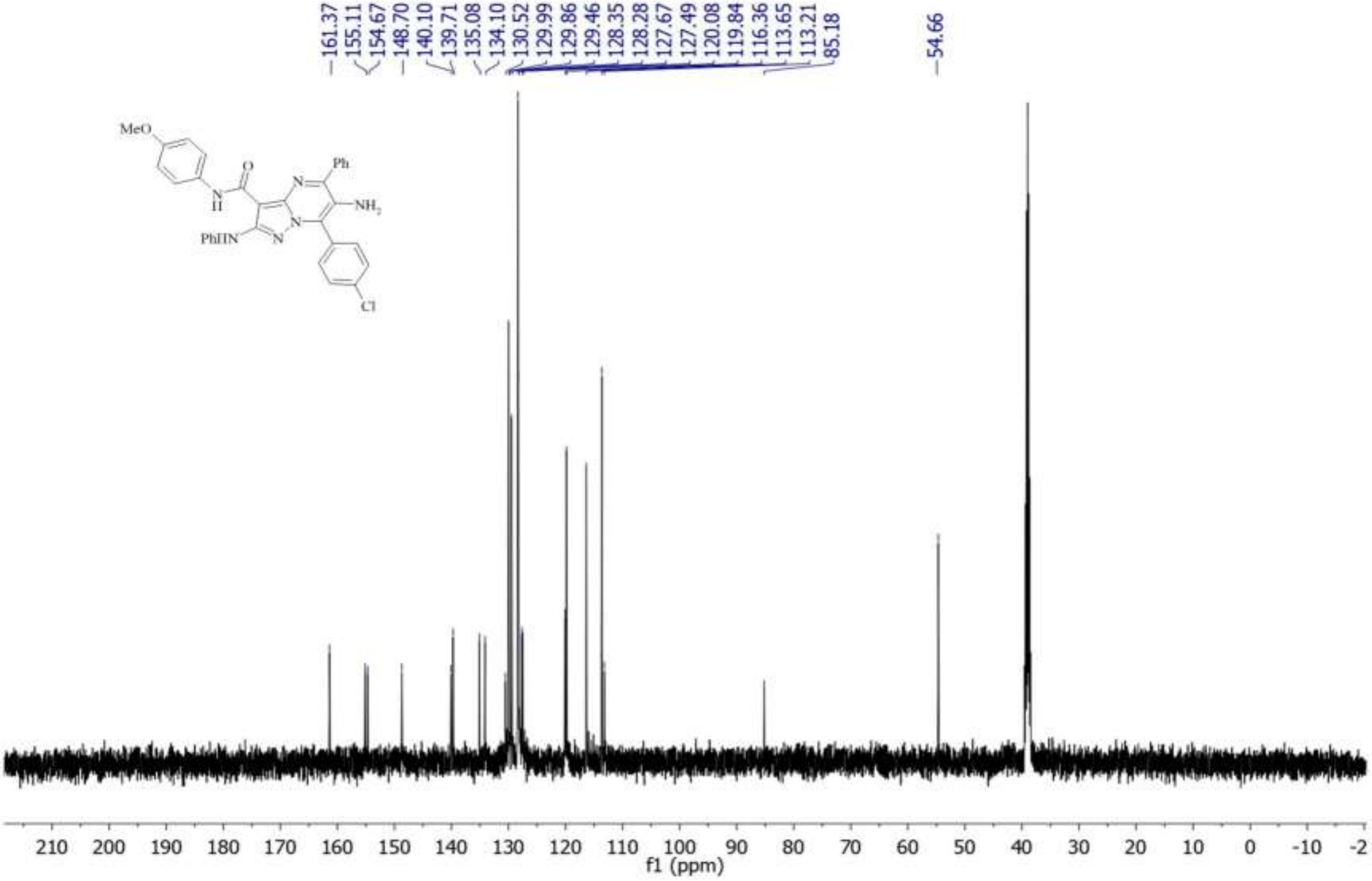

^{1}H NMR spectrum of 6-amino-7-(4-bromophenyl)-N-(4-methoxyphenyl)-5-phenyl-2-(phenylamino)pyrazolo[1,5-a]pyrimidine-3-carboxamide (**3k**)

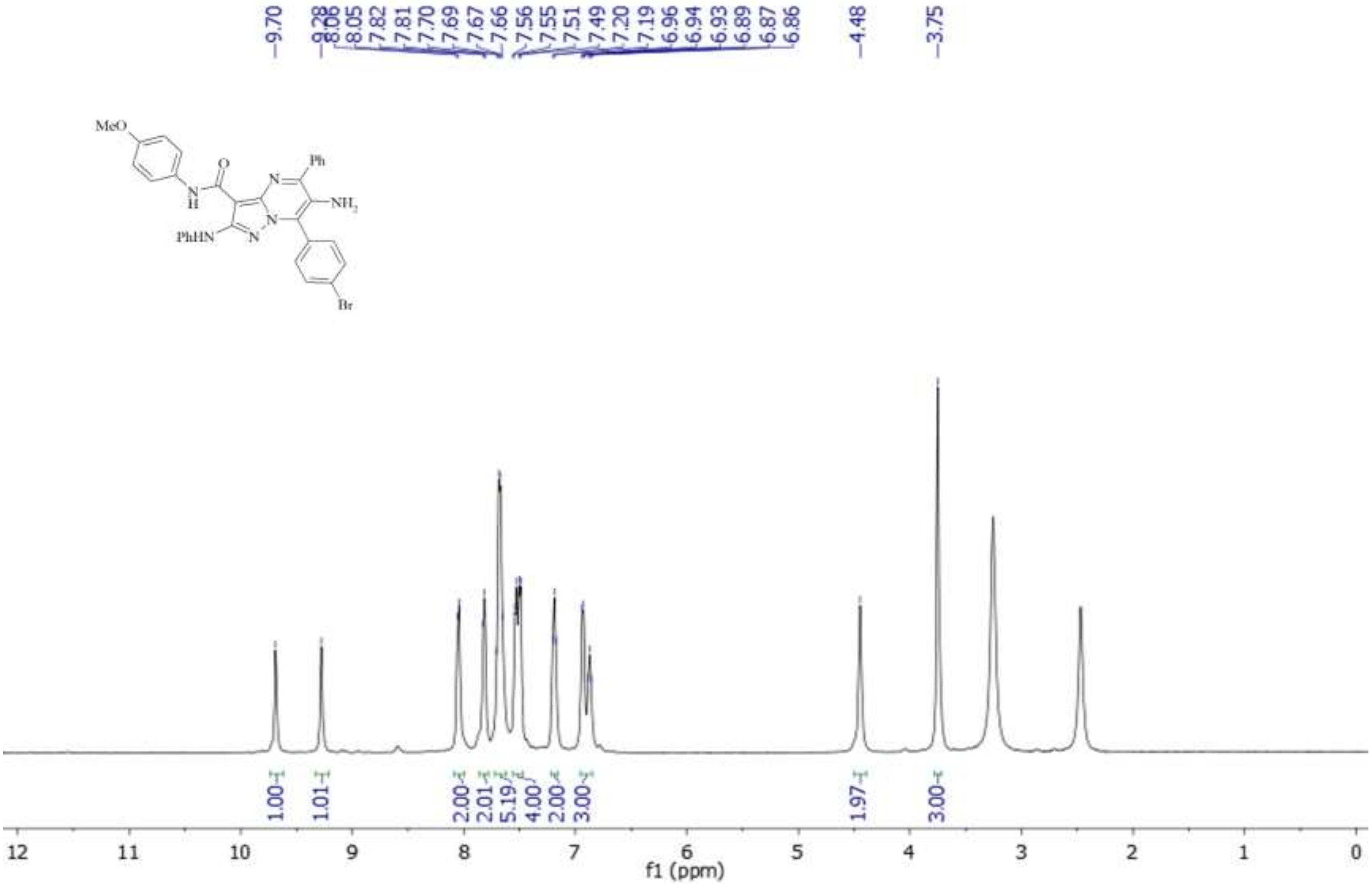

^{13}C NMR spectrum of 6-amino-7-(4-bromophenyl)-N-(4-methoxyphenyl)-5-phenyl-2-(phenylamino)pyrazolo[1,5-a]pyrimidine-3-carboxamide (**3k**)

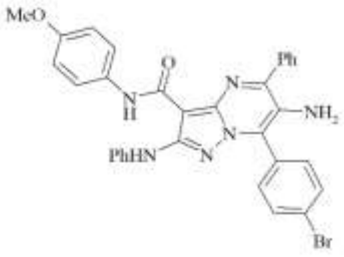

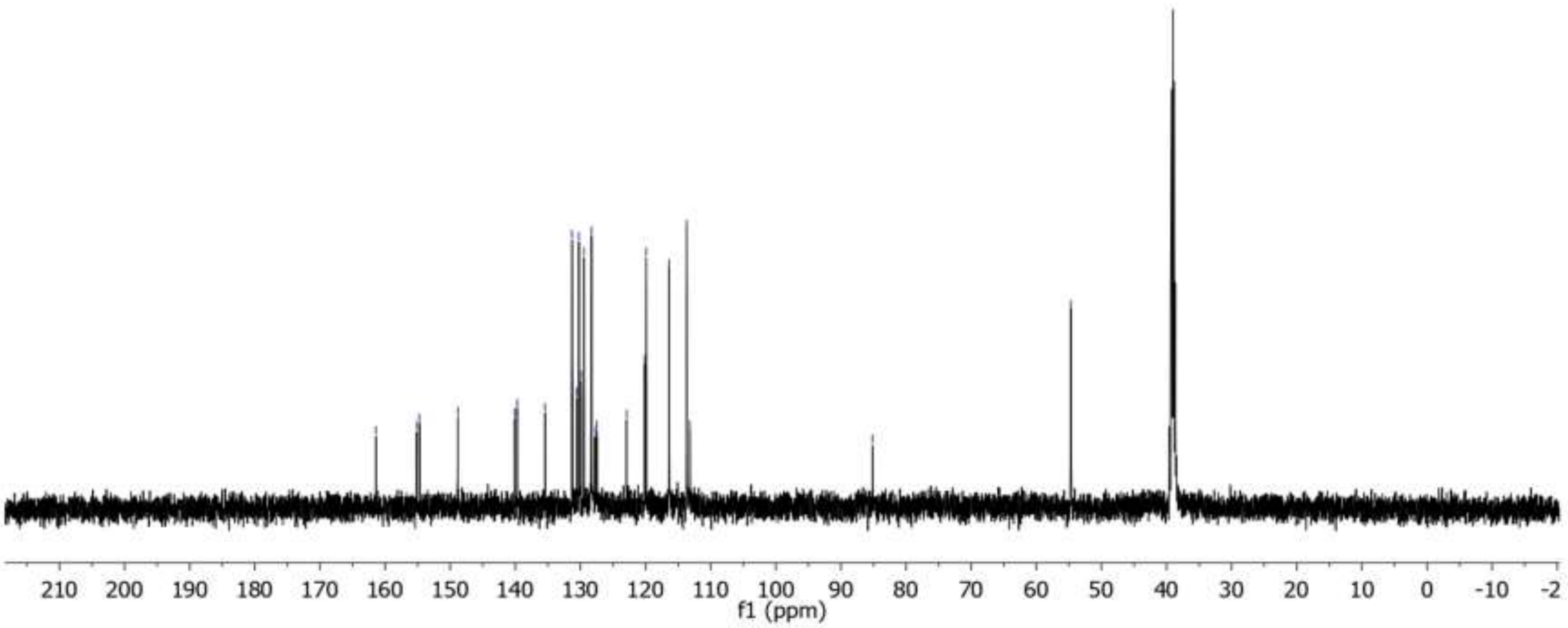

^{1}H NMR spectrum of 6-amino-7-(4-chlorophenyl)-N-(4-methoxyphenyl)-2-(phenylamino)-5-p-tolylpyrazolo[1,5-a]pyrimidine-3-carboxamide (**3l**)

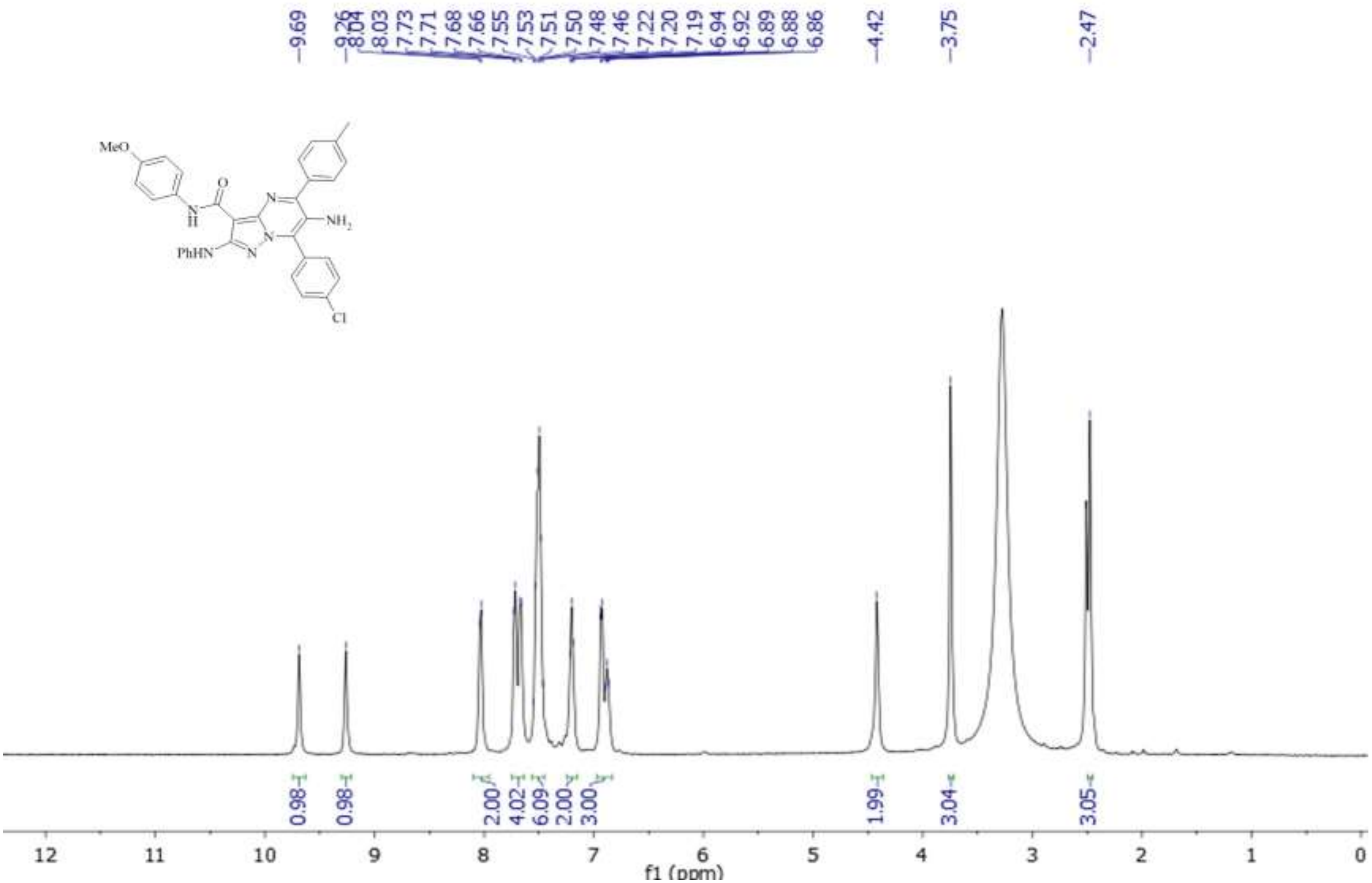

^{13}C NMR spectrum of 6-amino-7-(4-chlorophenyl)-N-(4-methoxyphenyl)-2-(phenylamino)-5-p-tolylpyrazolo[1,5-a]pyrimidine-3-carboxamide (**3l**)

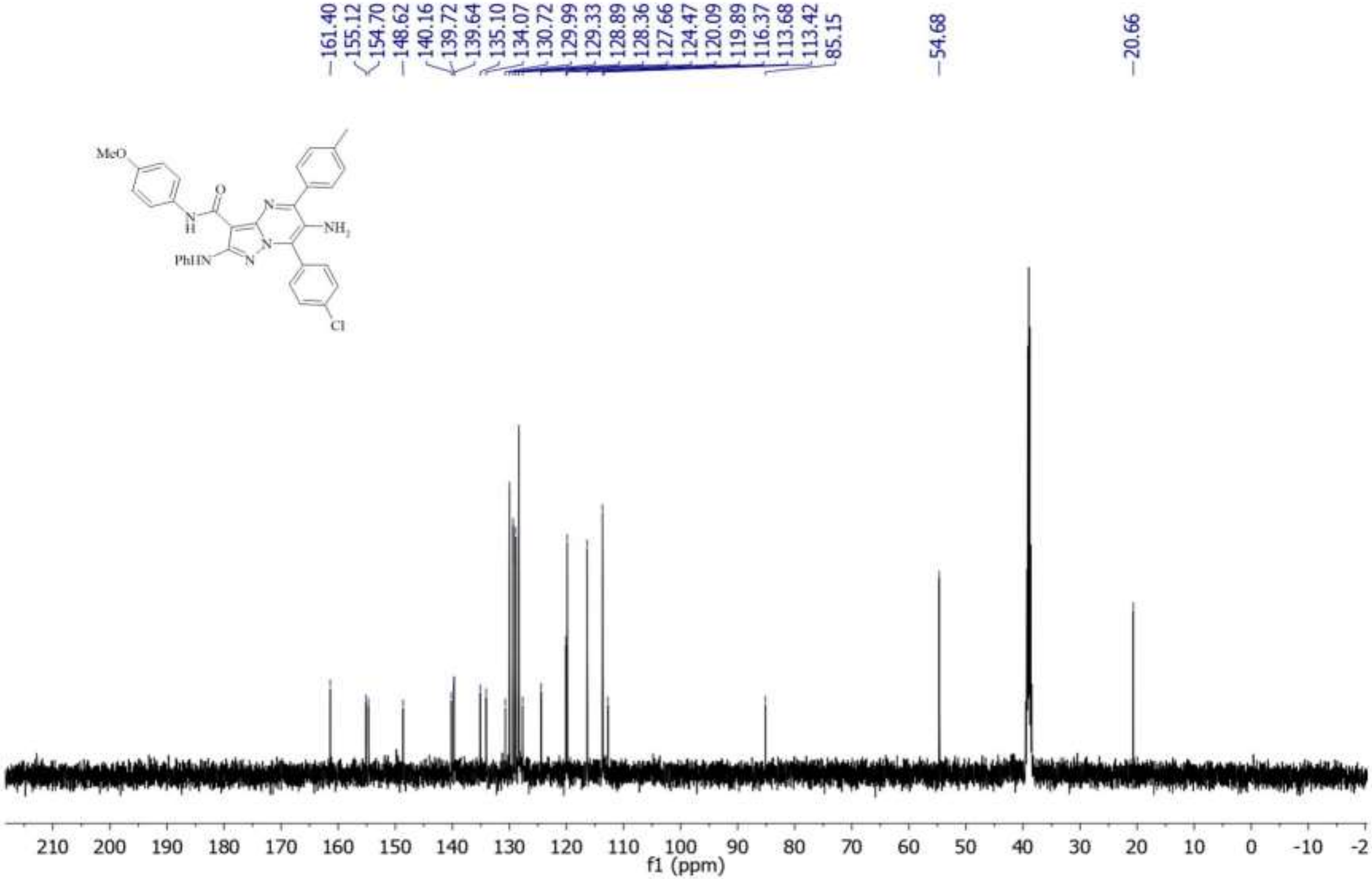

^{1}H NMR spectrum of 6-amino-7-(4-bromophenyl)-N-(4-methoxyphenyl)-2-(phenylamino)-5-p-tolylpyrazolo[1,5-a]pyrimidine-3-carboxamide (**3m**)

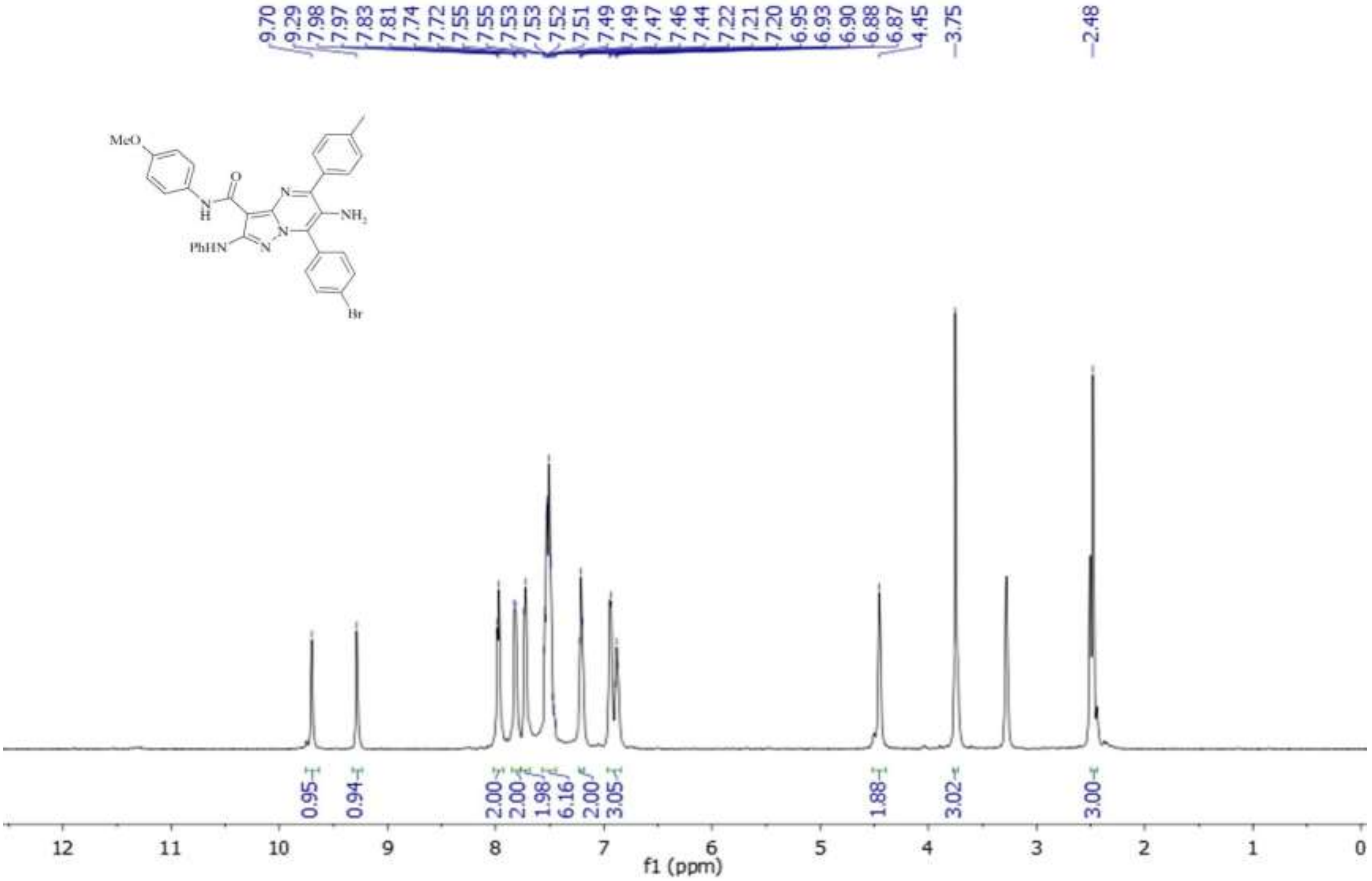

^{13}C NMR spectrum of 6-amino-7-(4-bromophenyl)-N-(4-methoxyphenyl)-2-(phenylamino)-5-p-tolylpyrazolo[1,5-a]pyrimidine-3-carboxamide (**3m**)

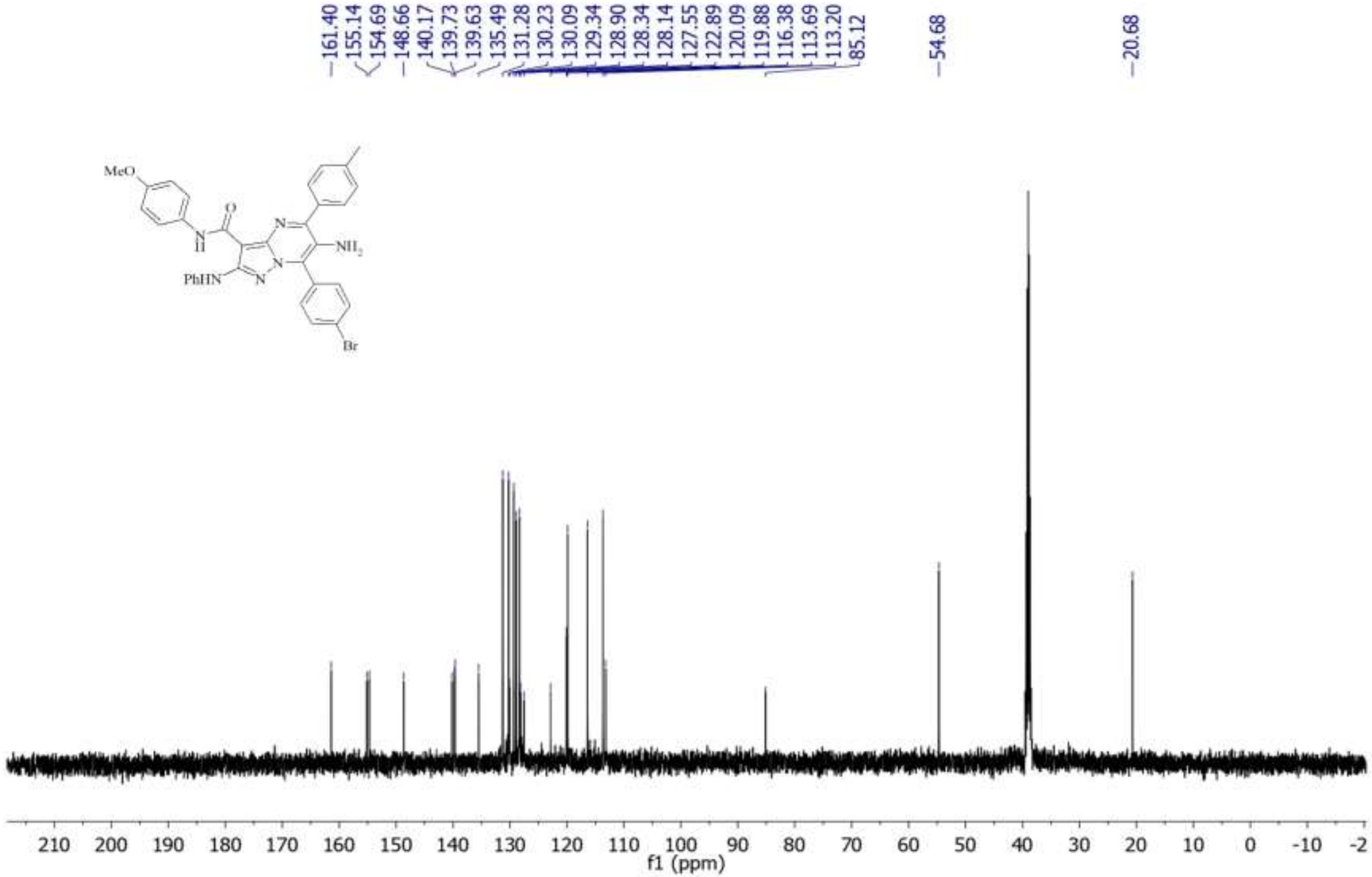

^{1}H NMR spectrum of 6-amino-N,5-bis(4-methoxyphenyl)-7-phenyl-2-(phenylamino)pyrazolo[1,5-a]pyrimidine-3-carboxamide (**3n**)

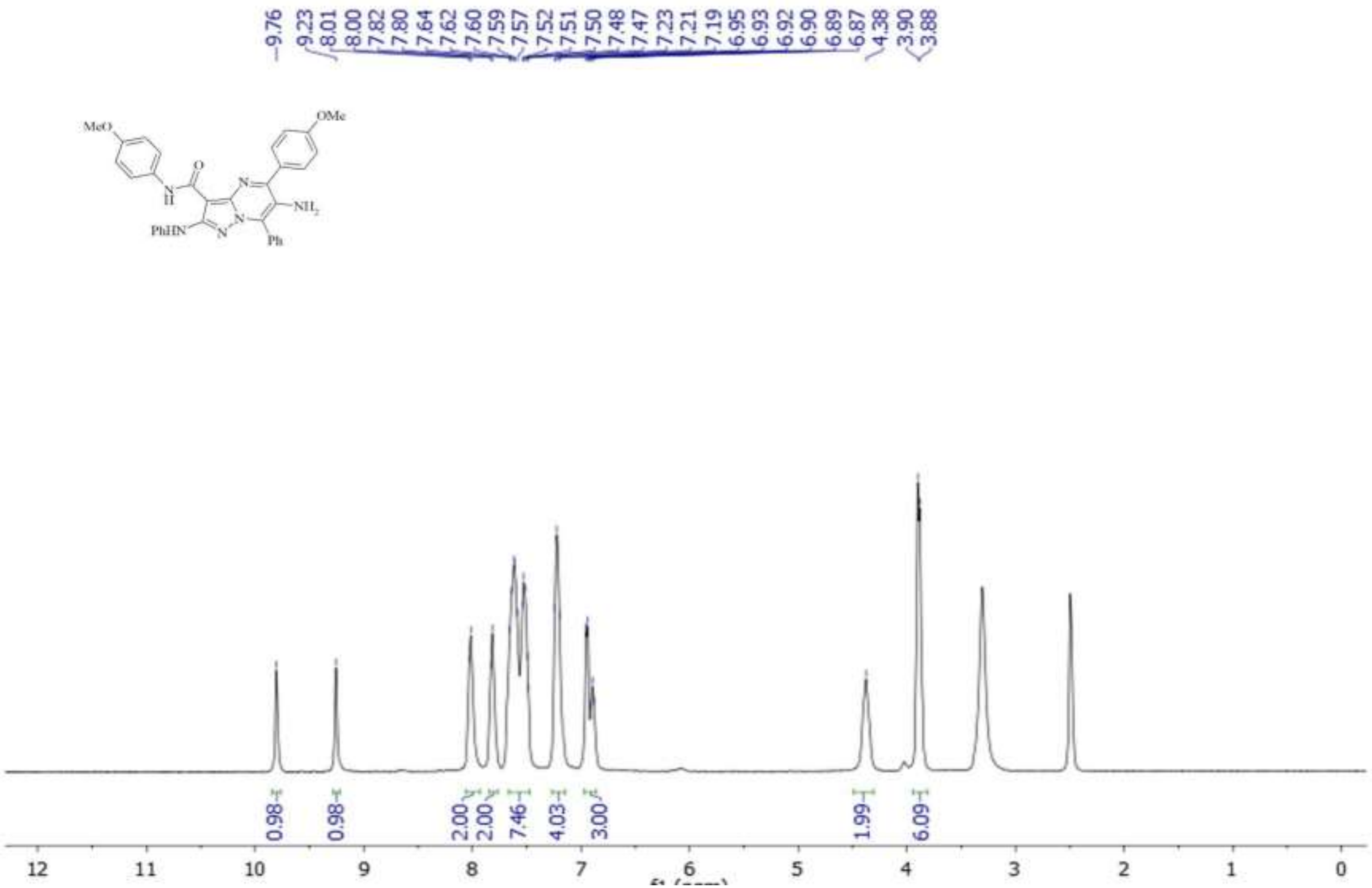

^{13}C NMR spectrum of 6-amino-N,5-bis(4-methoxyphenyl)-7-phenyl-2-(phenylamino)pyrazolo[1,5-a]pyrimidine-3-carboxamide (**3n**)

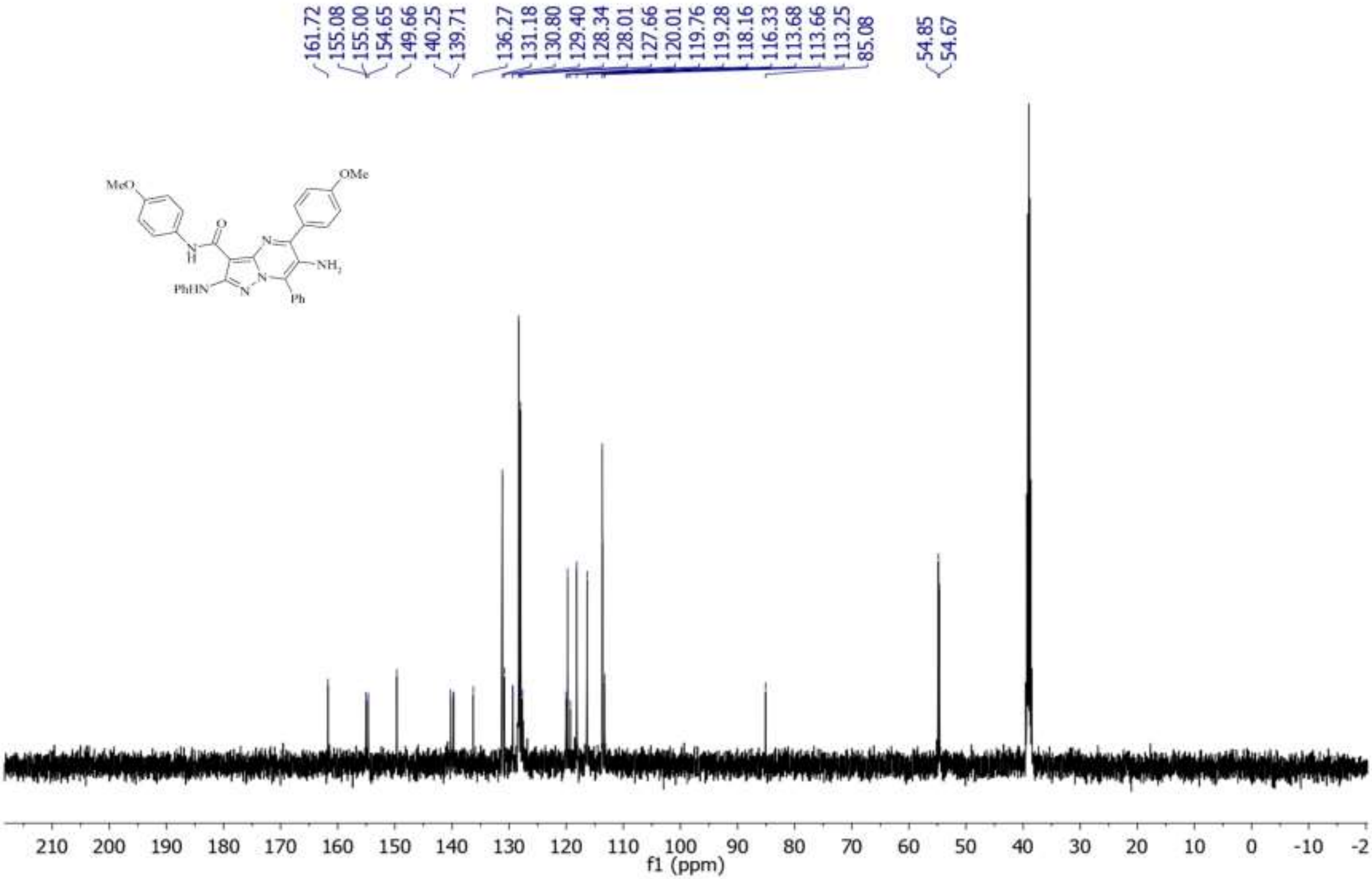

^{1}H NMR spectrum of 6-amino-7-(4-chlorophenyl)-N,5-bis(4-methoxyphenyl)-2-(phenylamino)pyrazolo[1,5-a]pyrimidine-3-carboxamide (**3o**)

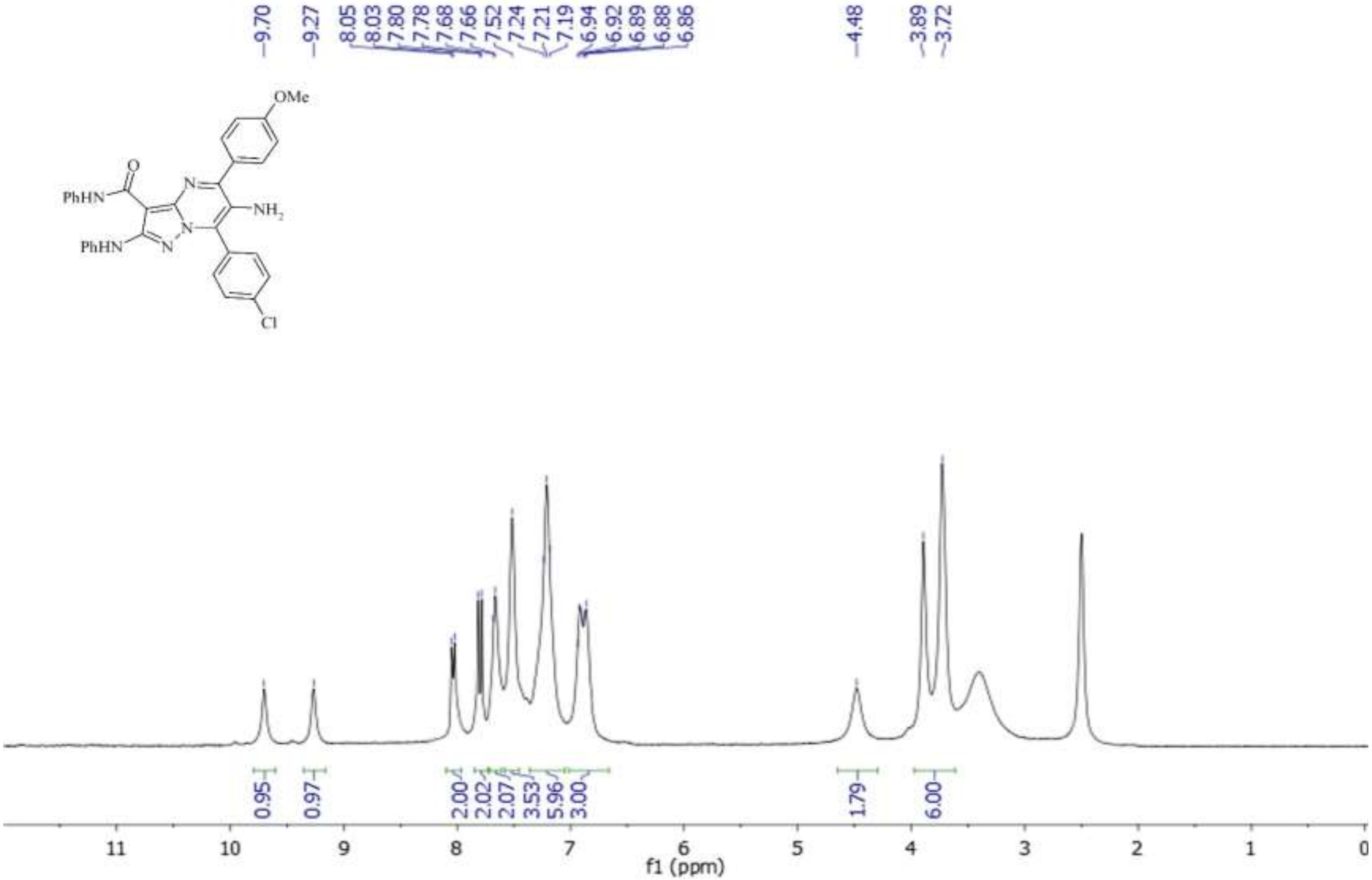

^{13}C NMR spectrum of 6-amino-7-(4-chlorophenyl)-N,5-bis(4-methoxyphenyl)-2-(phenylamino)pyrazolo[1,5-a]pyrimidine-3-carboxamide (**3o**)

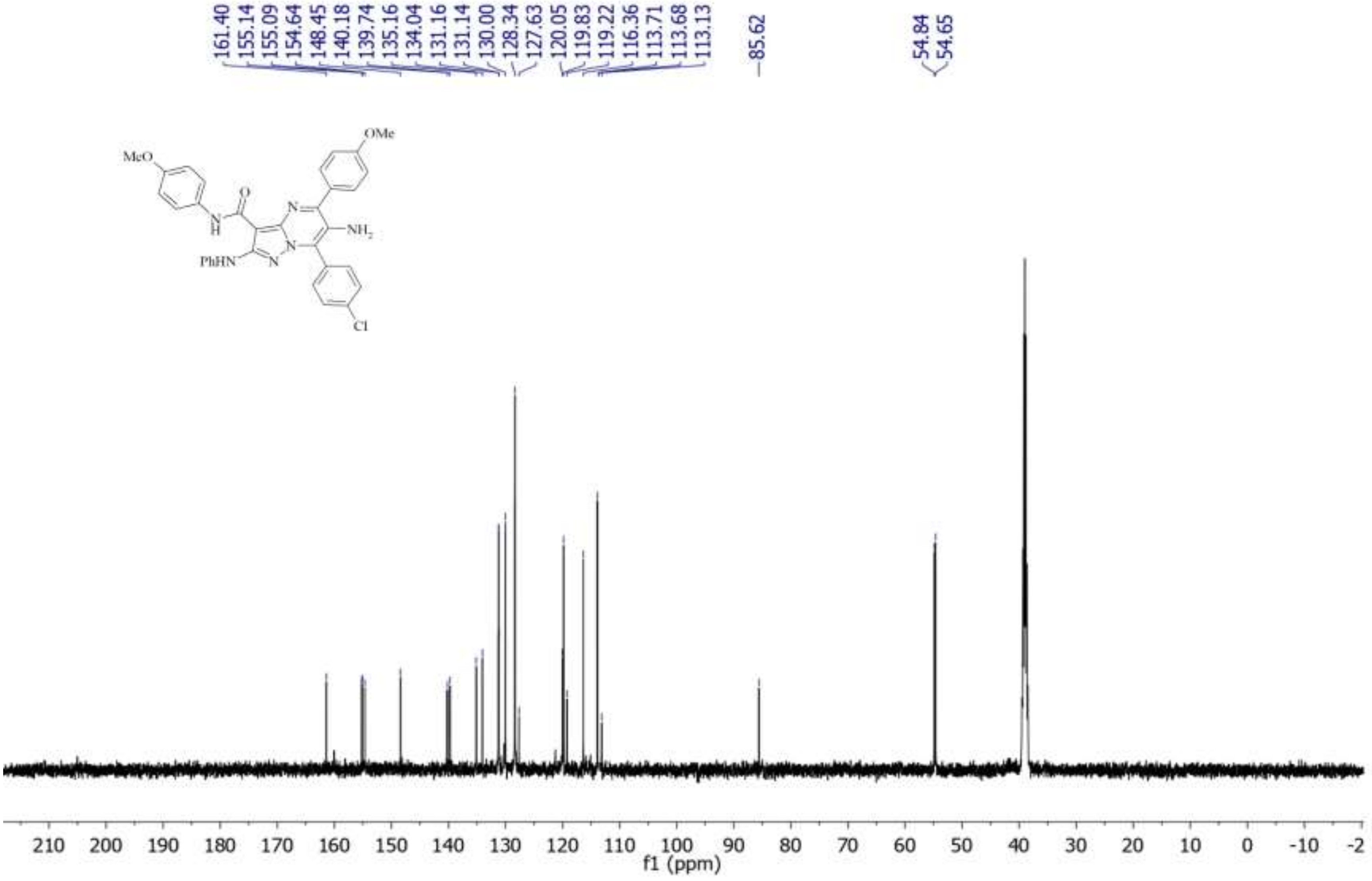

^{1}H NMR spectrum of 6-amino-5-(4-chlorophenyl)-N-(4-methoxyphenyl)-7-phenyl-2-(phenylamino)pyrazolo[1,5-a]pyrimidine-3-carboxamide (**3p**)

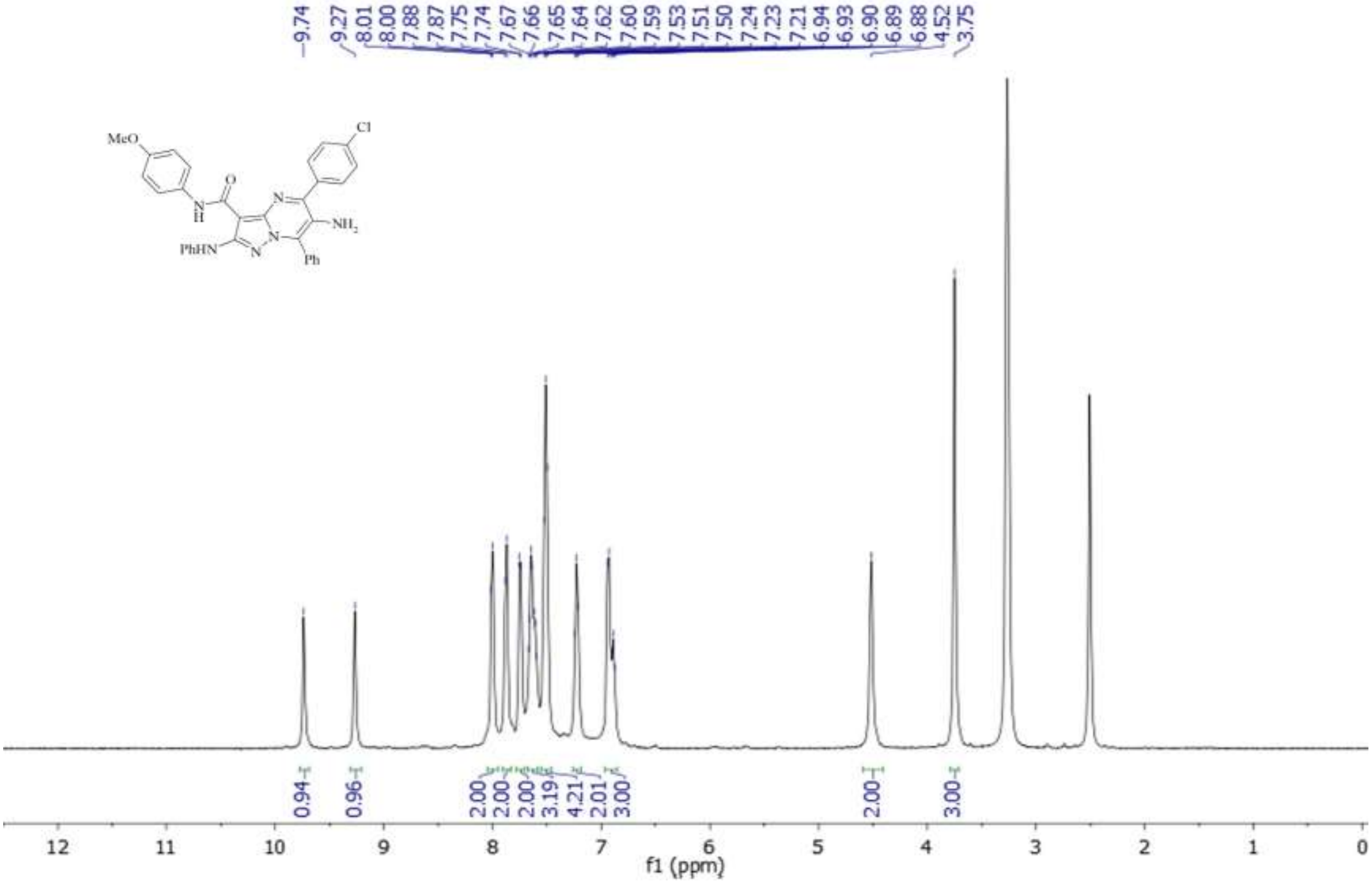

^{13}C NMR spectrum of 6-amino-5-(4-chlorophenyl)-N-(4-methoxyphenyl)-7-phenyl-2-(phenylamino)pyrazolo[1,5-a]pyrimidine-3-carboxamide (**3p**)

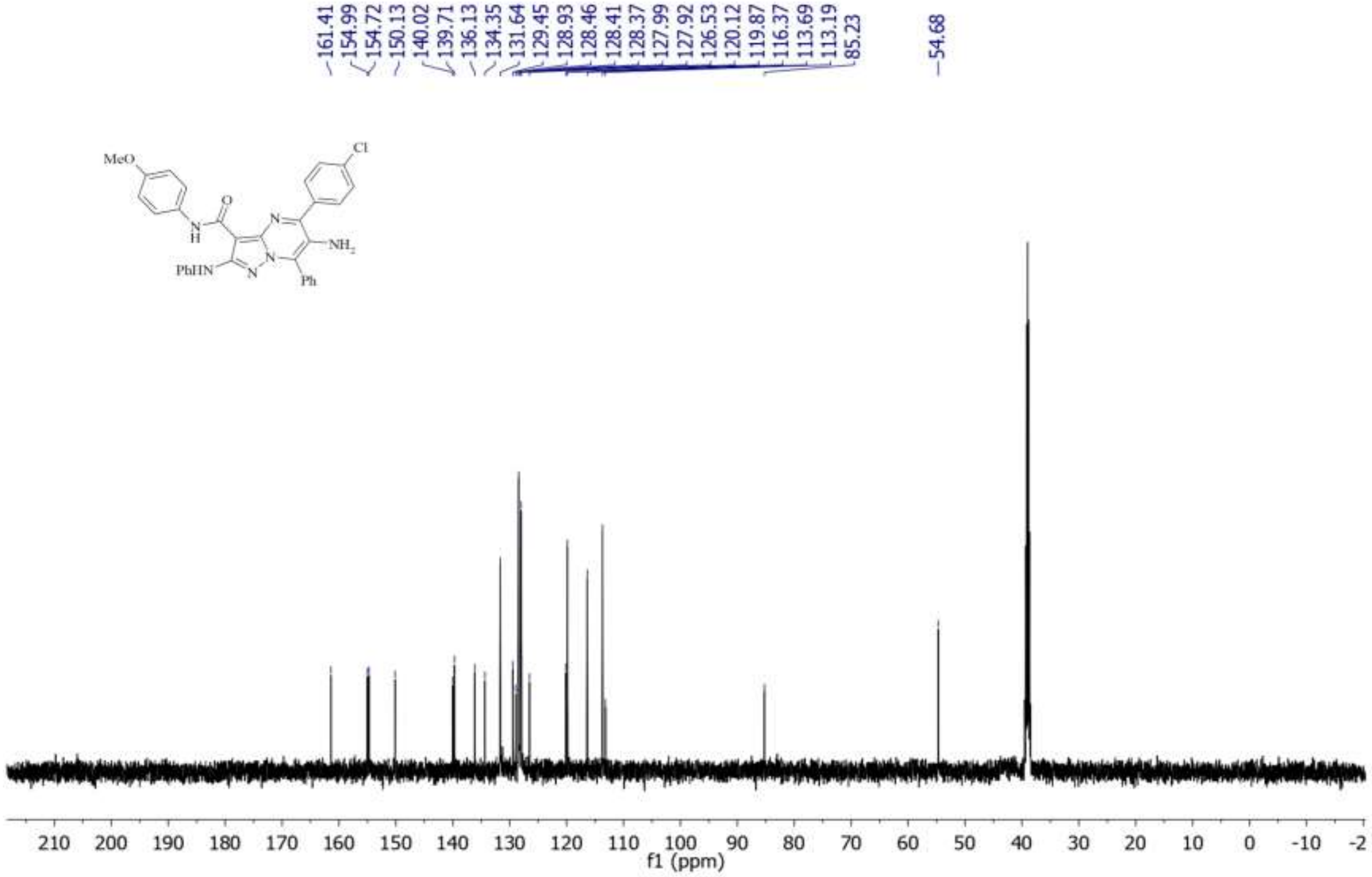

^{1}H NMR spectrum of 6-amino-5,7-bis(4-chlorophenyl)-N-(4-methoxyphenyl)-2-(phenylamino)pyrazolo[1,5-a]pyrimidine-3-carboxamide (**3q**)

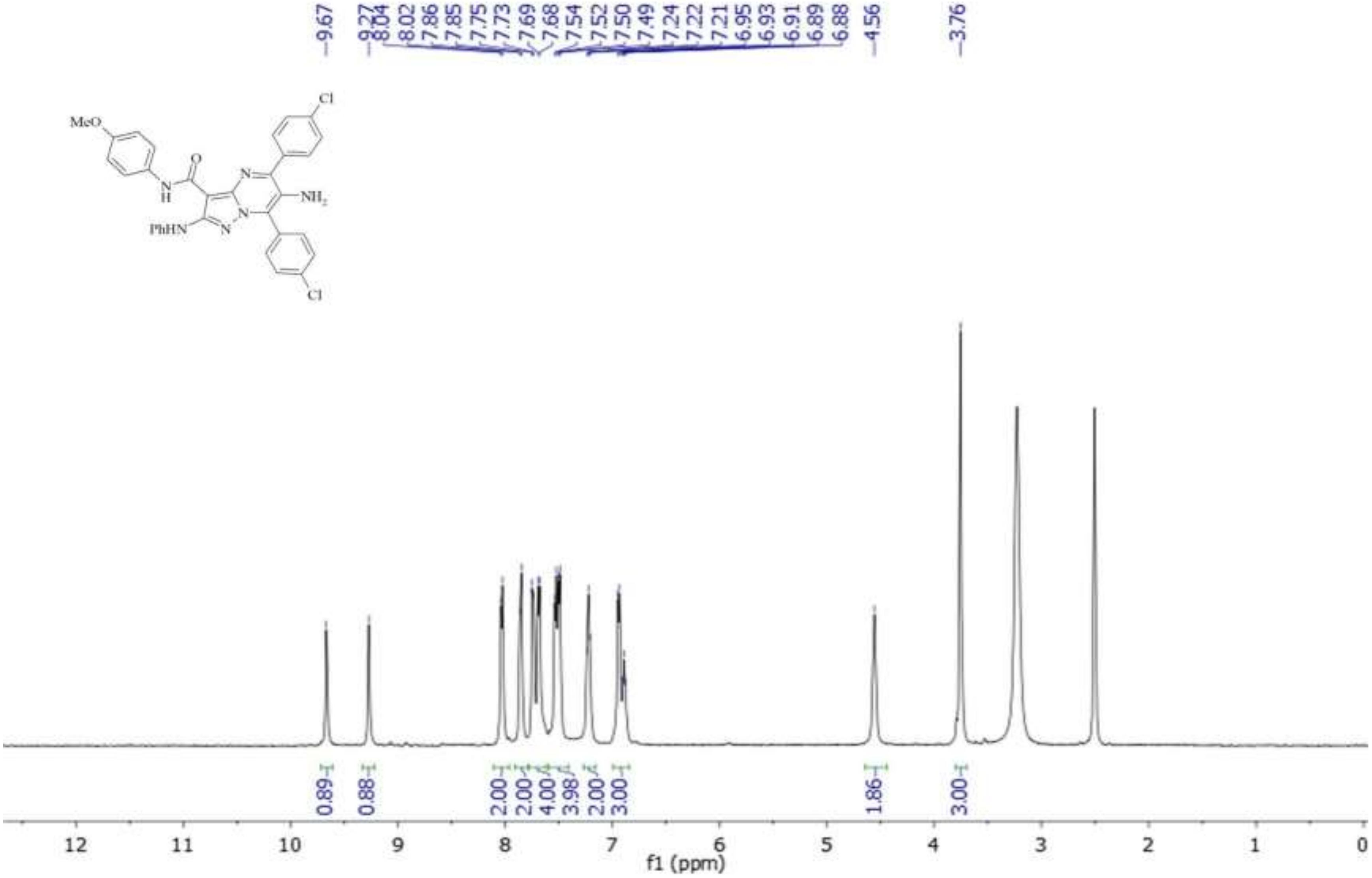

^{13}C NMR spectrum of 6-amino-5,7-bis(4-chlorophenyl)-N-(4-methoxyphenyl)-2-(phenylamino)pyrazolo[1,5-a]pyrimidine-3-carboxamide (**3q**)

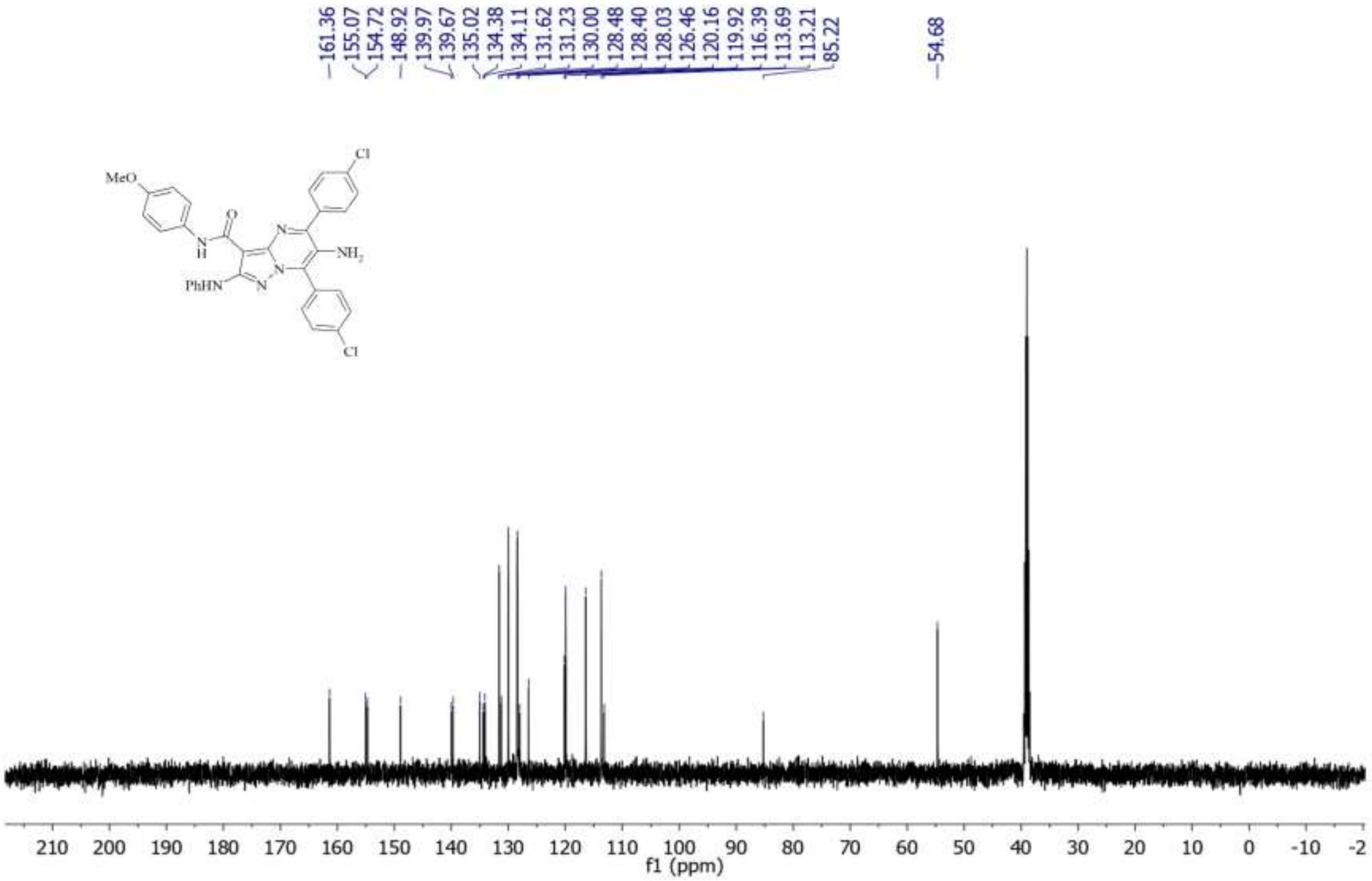

^{1}H NMR spectrum of 6-amino-5-(4-chlorophenyl)-N-(4-methoxyphenyl)-2-(phenylamino)-7-(thiophen-2-yl)pyrazolo[1,5-a]pyrimidine-3-carboxamide (**3r**)

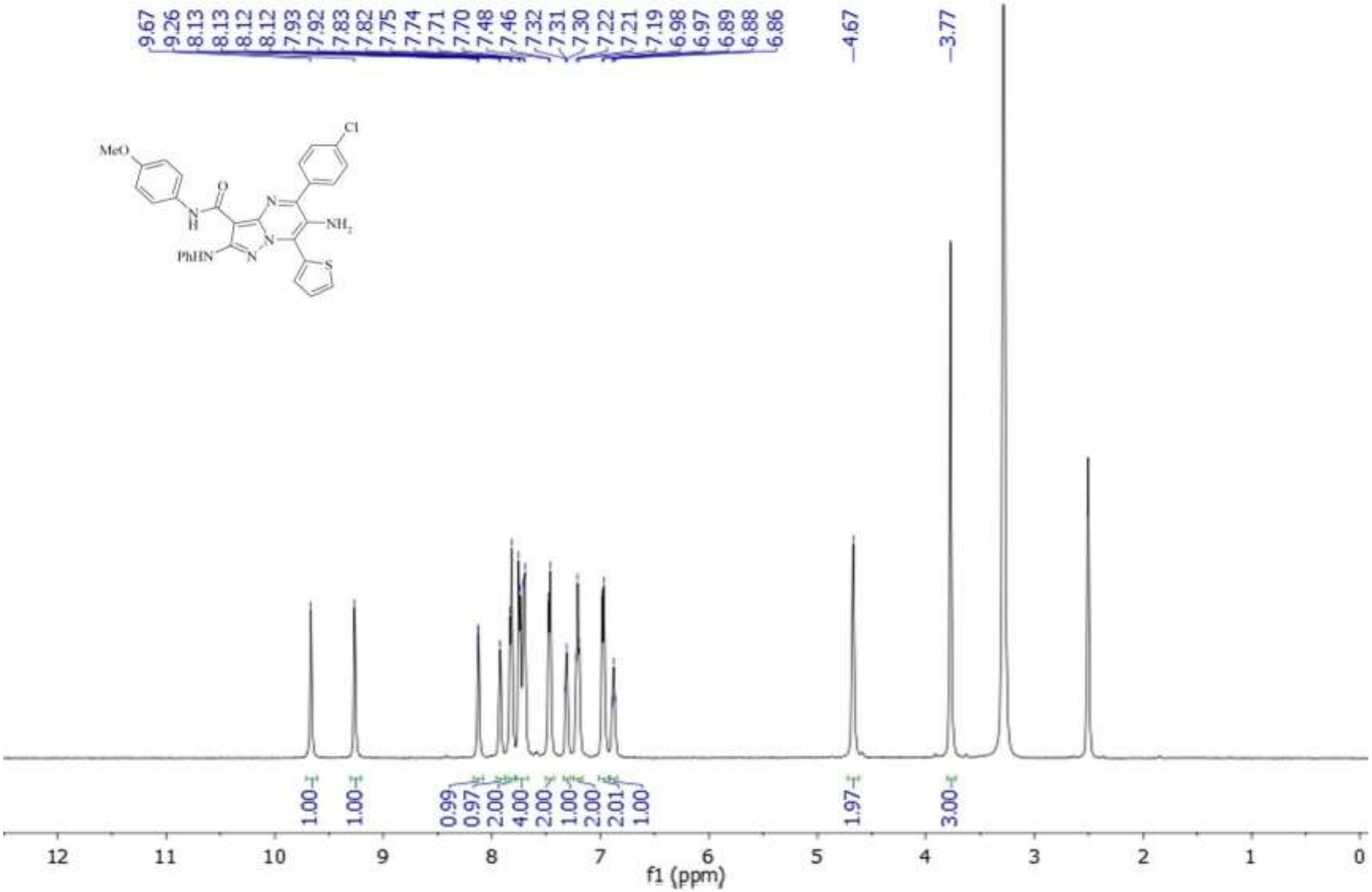

^{13}C NMR spectrum of 6-amino-5-(4-chlorophenyl)-N-(4-methoxyphenyl)-2-(phenylamino)-7-(thiophen-2-yl)pyrazolo[1,5-a]pyrimidine-3-carboxamide (**3r**)

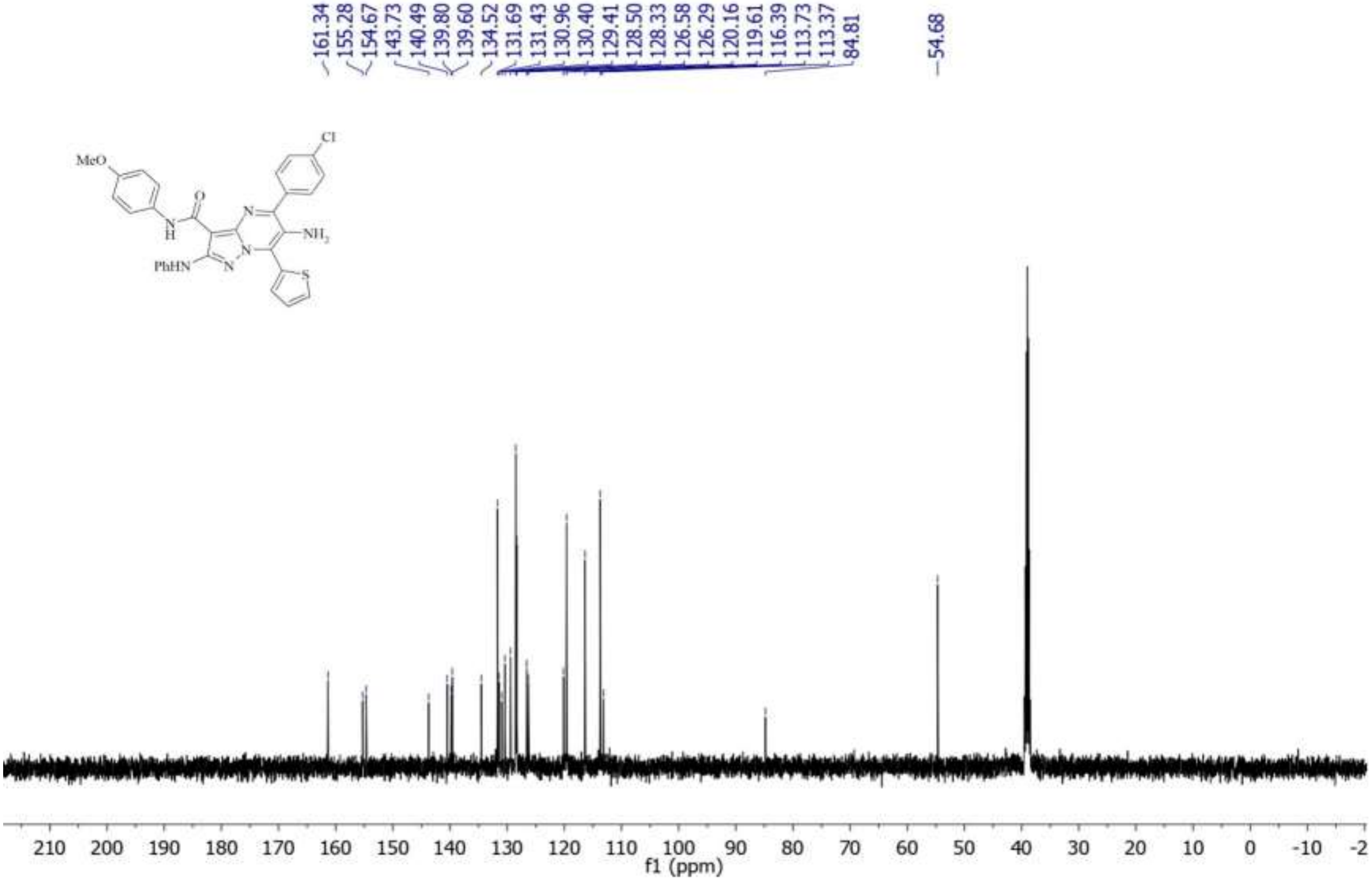

^{1}H NMR spectrum of 6-amino-N,7-bis(4-chlorophenyl)-5-phenyl-2-(phenylamino)pyrazolo[1,5-a]pyrimidine-3-carboxamide (**3s**)

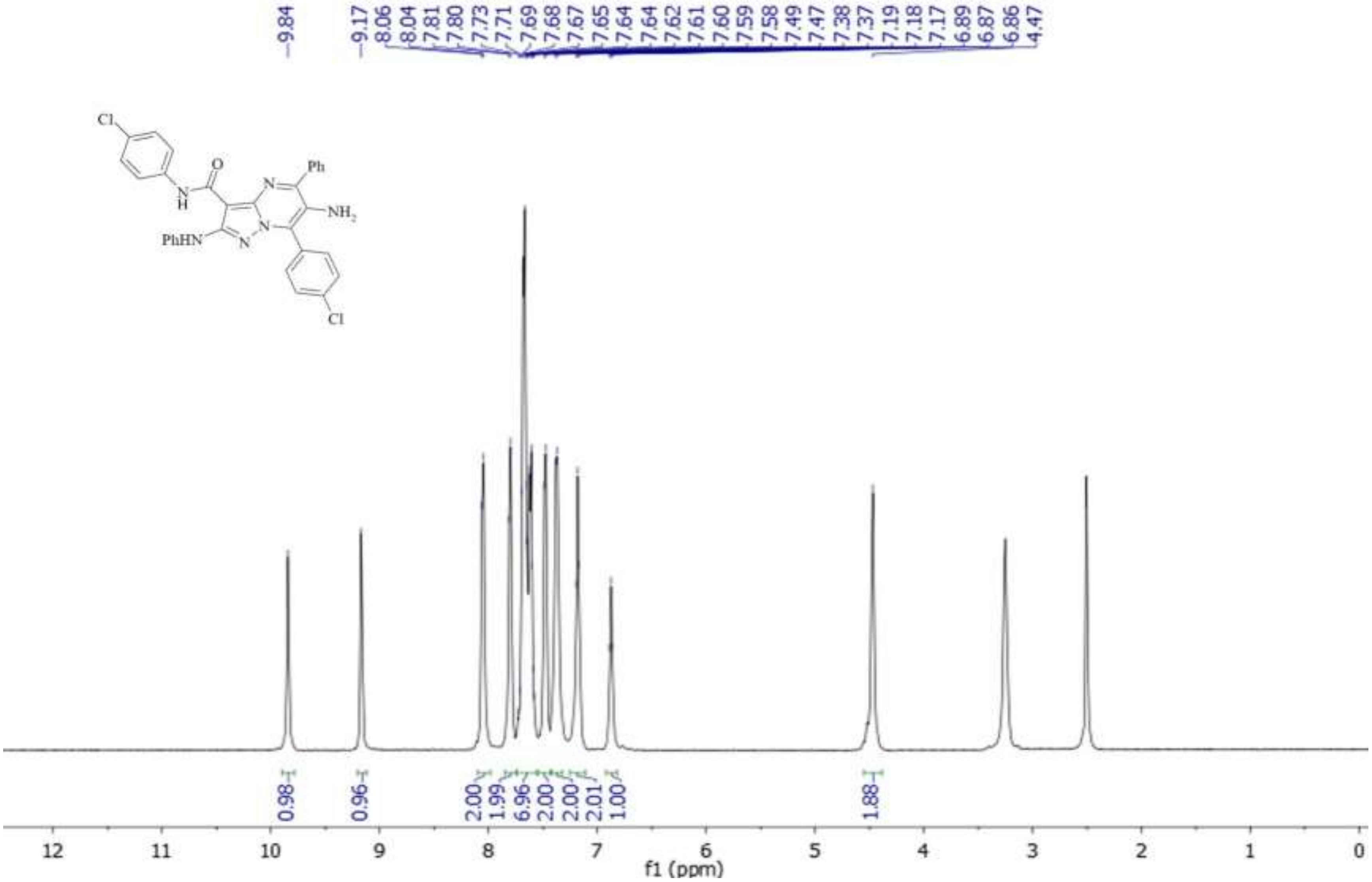

^{13}C NMR spectrum of 6-amino-N,7-bis(4-chlorophenyl)-5-phenyl-2-(phenylamino)pyrazolo[1,5-a]pyrimidine-3-carboxamide (**3s**)

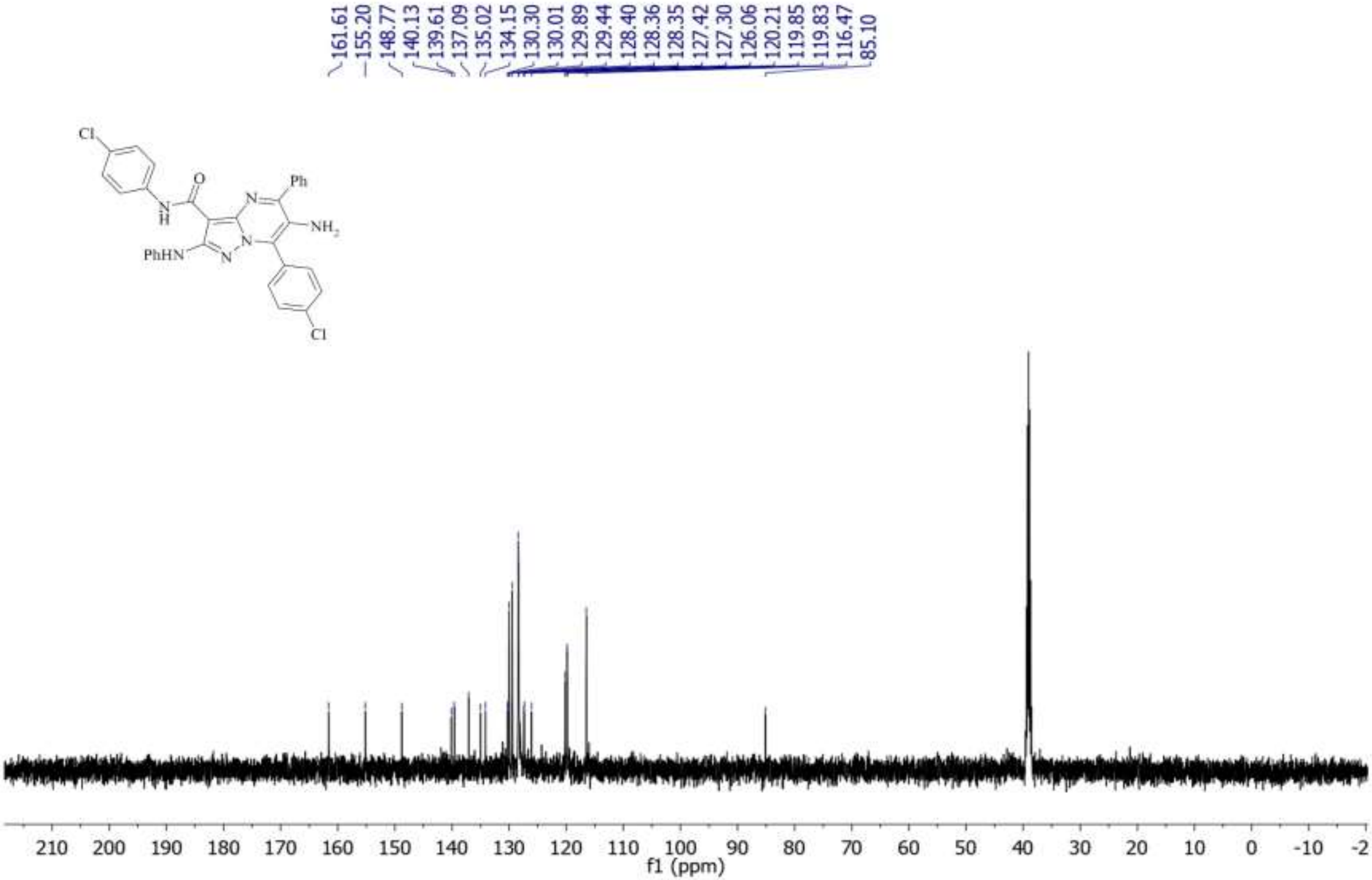

^{1}H NMR spectrum of 6-amino-7-(4-bromophenyl)-N-(4-chlorophenyl)-5-phenyl-2-(phenylamino)pyrazolo[1,5-a]pyrimidine-3-carboxamide (**3t**)

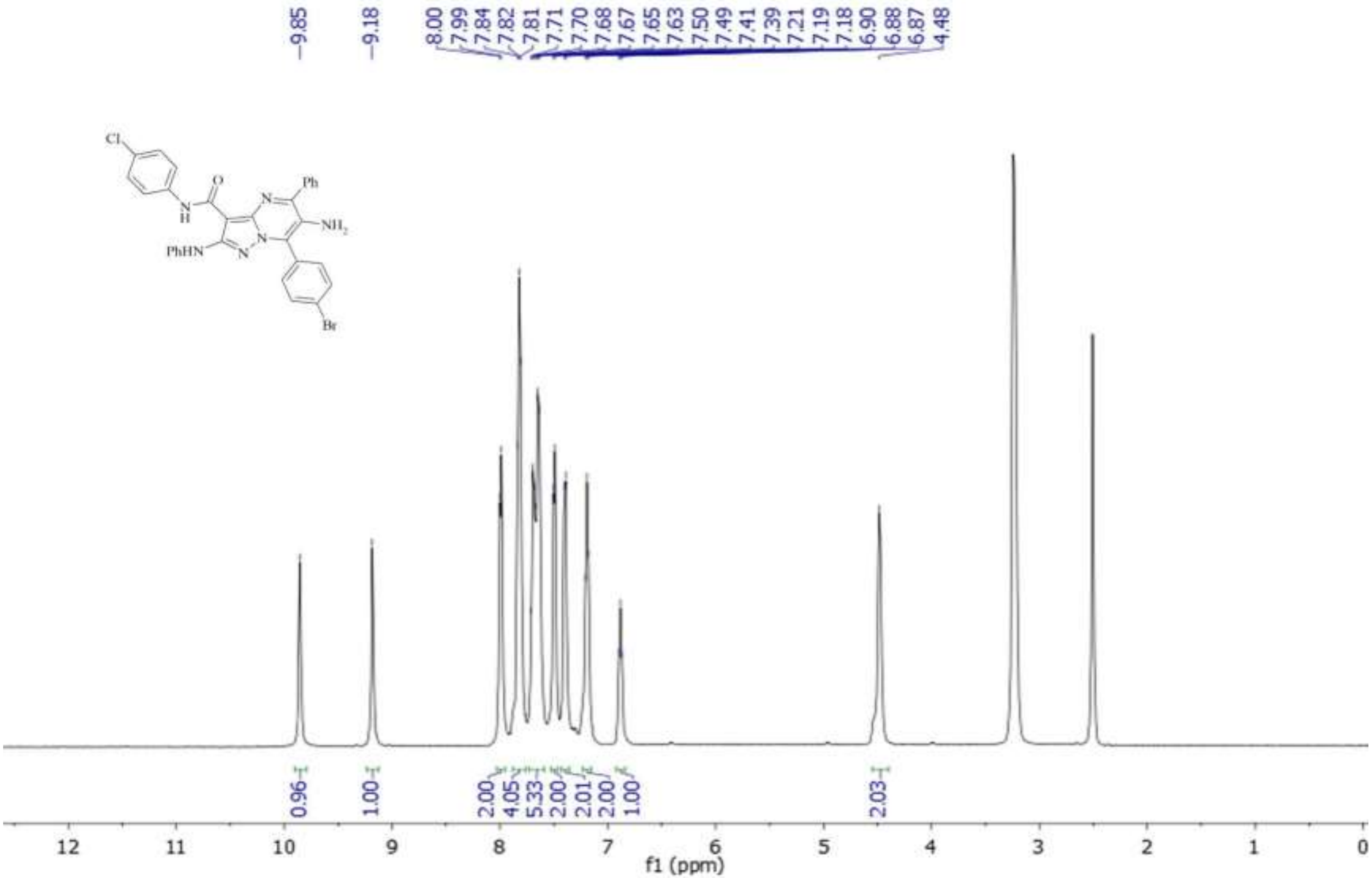

^{13}C NMR spectrum of 6-amino-7-(4-bromophenyl)-N-(4-chlorophenyl)-5-phenyl-2-(phenylamino)pyrazolo[1,5-a]pyrimidine-3-carboxamide (**3t**)

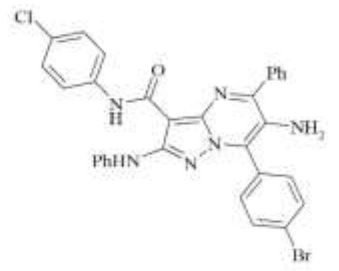

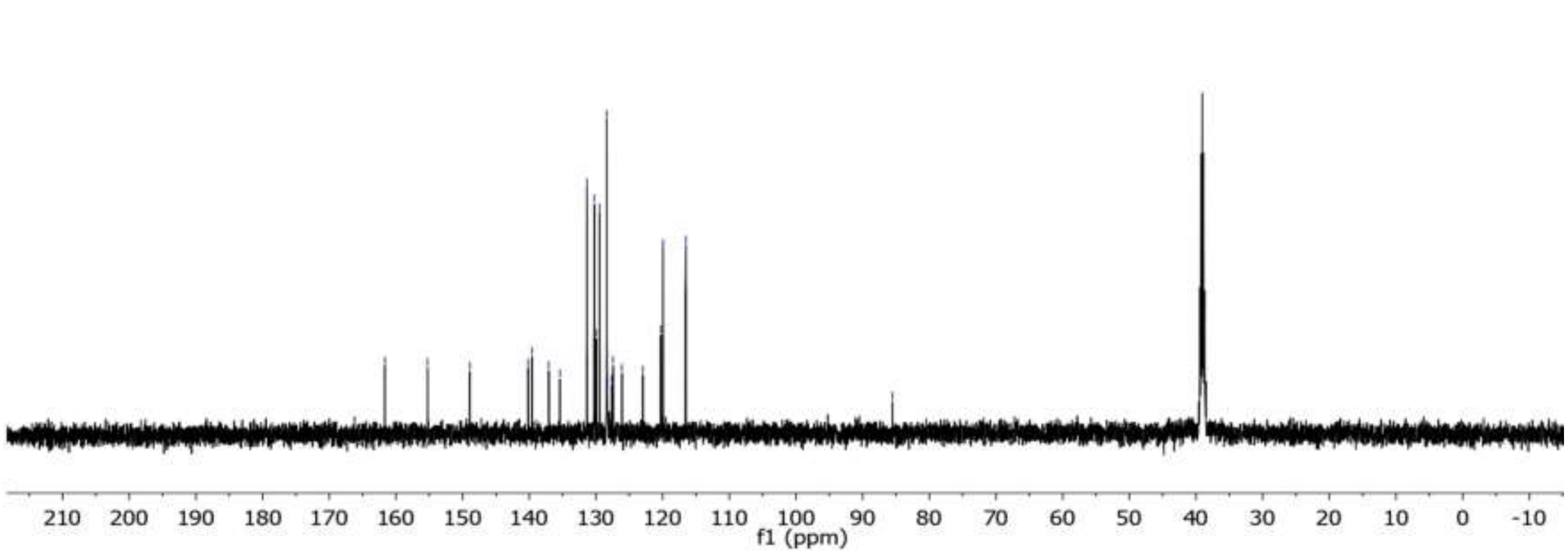

^{1}H NMR spectrum of 6-amino-N,7-bis(4-chlorophenyl)-2-(phenylamino)-5-p-tolylpyrazolo[1,5-a]pyrimidine-3-carboxamide (**3u**)

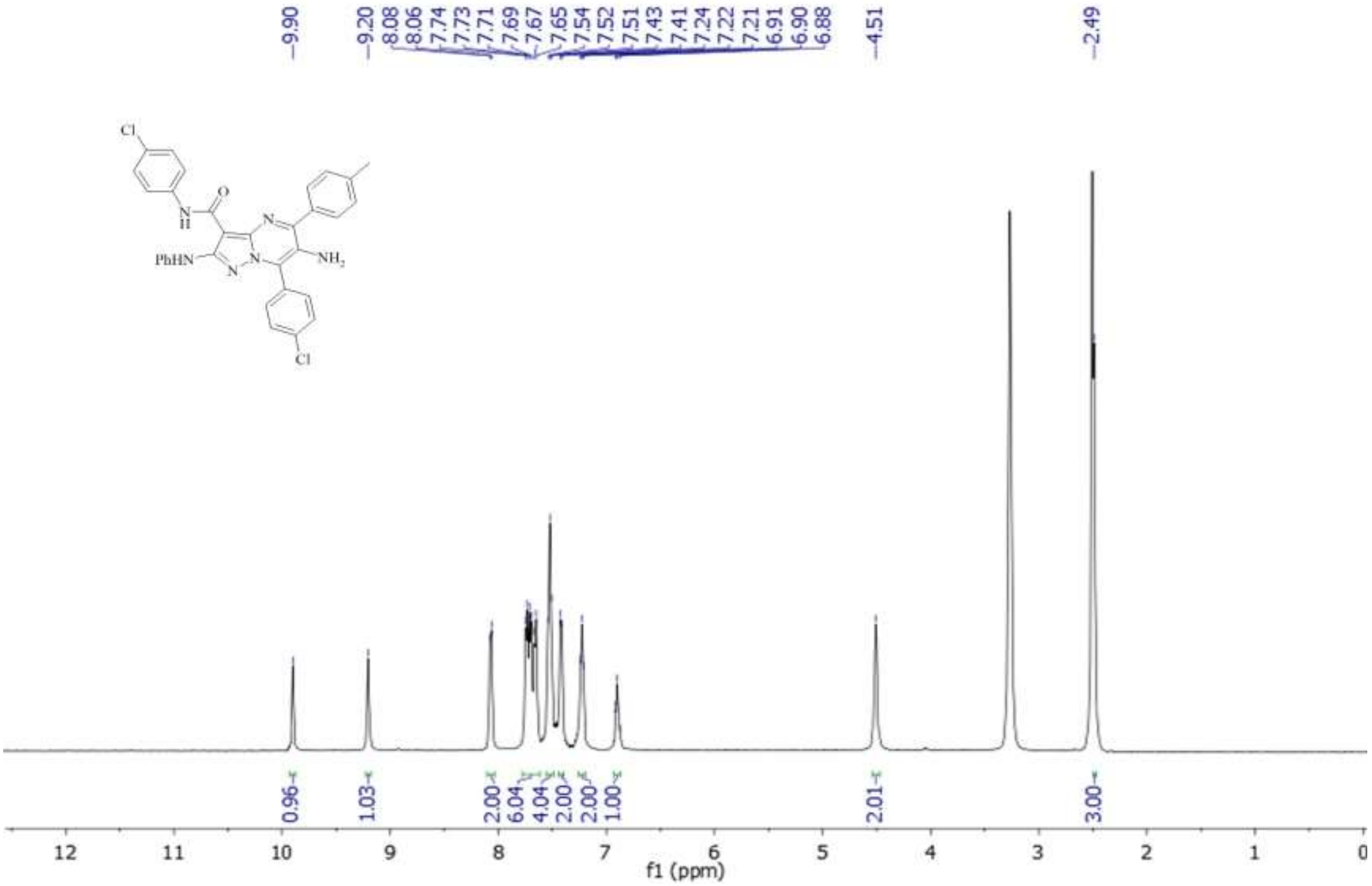

^{13}C NMR spectrum of 6-amino-N,7-bis(4-chlorophenyl)-2-(phenylamino)-5-p-tolylpyrazolo[1,5-a]pyrimidine-3-carboxamide (**3u**)

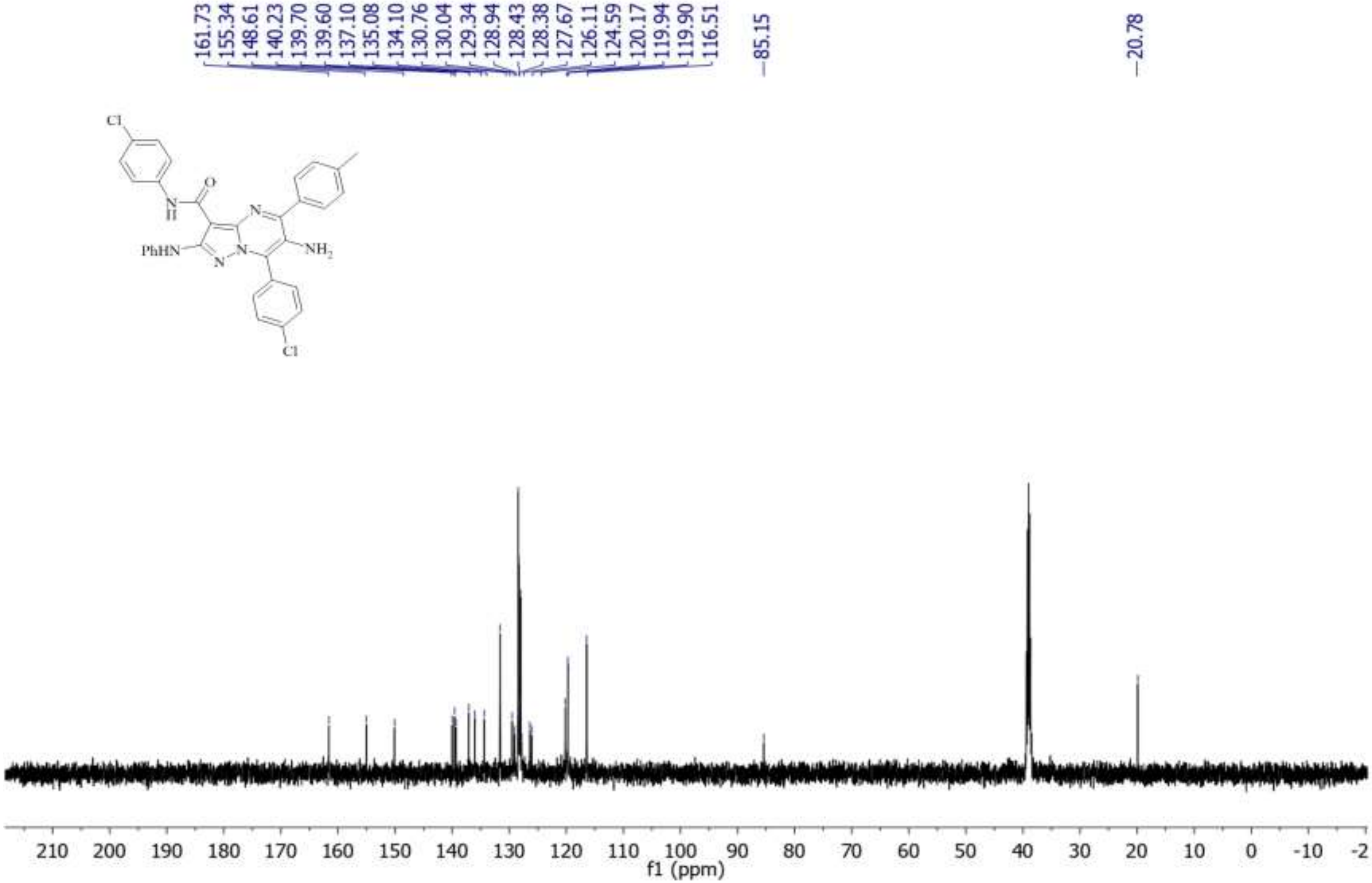

^{1}H NMR spectrum of 6-amino-7-(4-bromophenyl)-N-(4-chlorophenyl)-2-(phenylamino)-5-p-tolylpyrazolo[1,5-a]pyrimidine-3-carboxamide (**3v**)

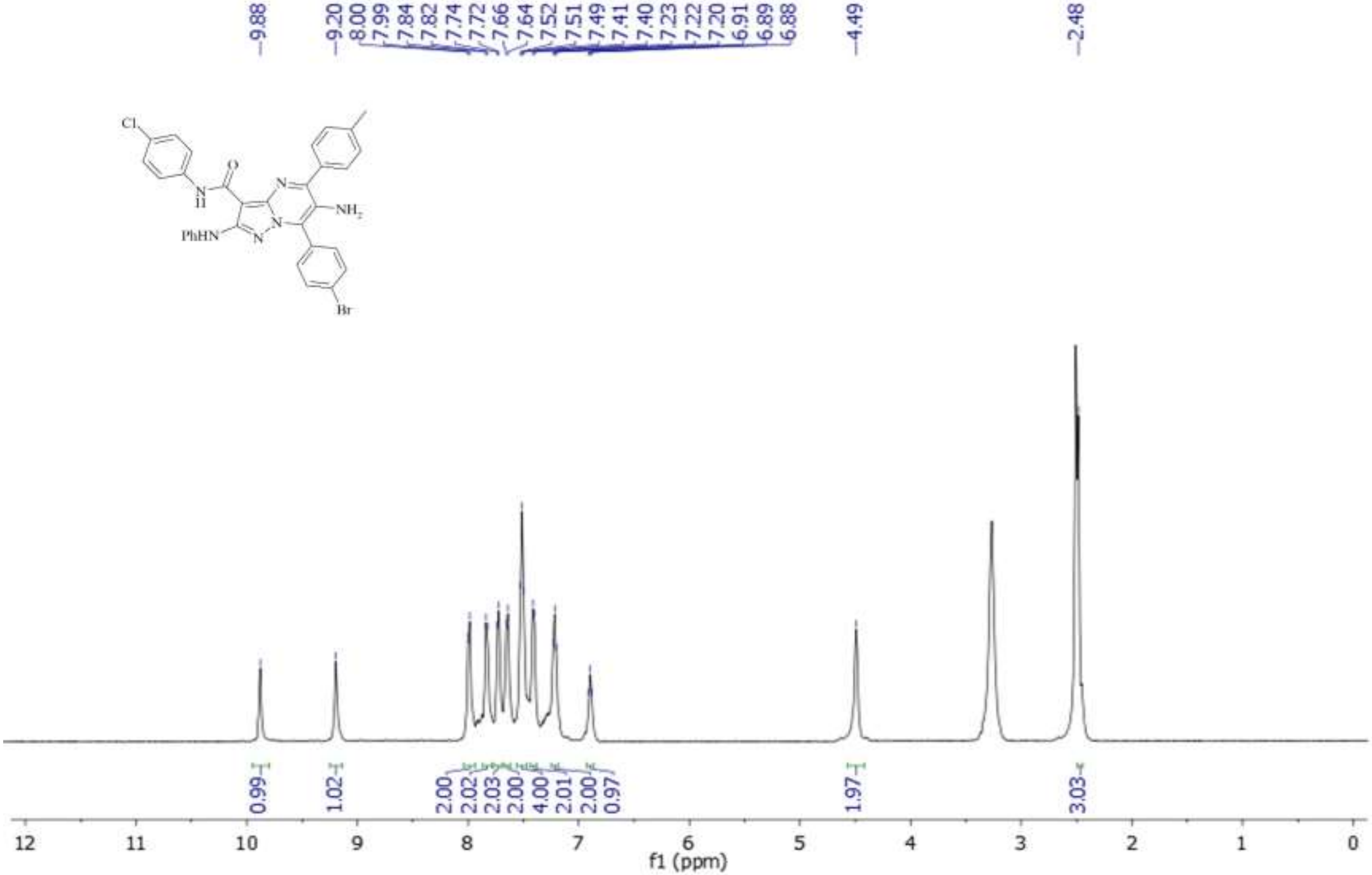

^{13}C NMR spectrum of 6-amino-7-(4-bromophenyl)-N-(4-chlorophenyl)-2-(phenylamino)-5-p-tolylpyrazolo[1,5-a]pyrimidine-3-carboxamide (**3v**)

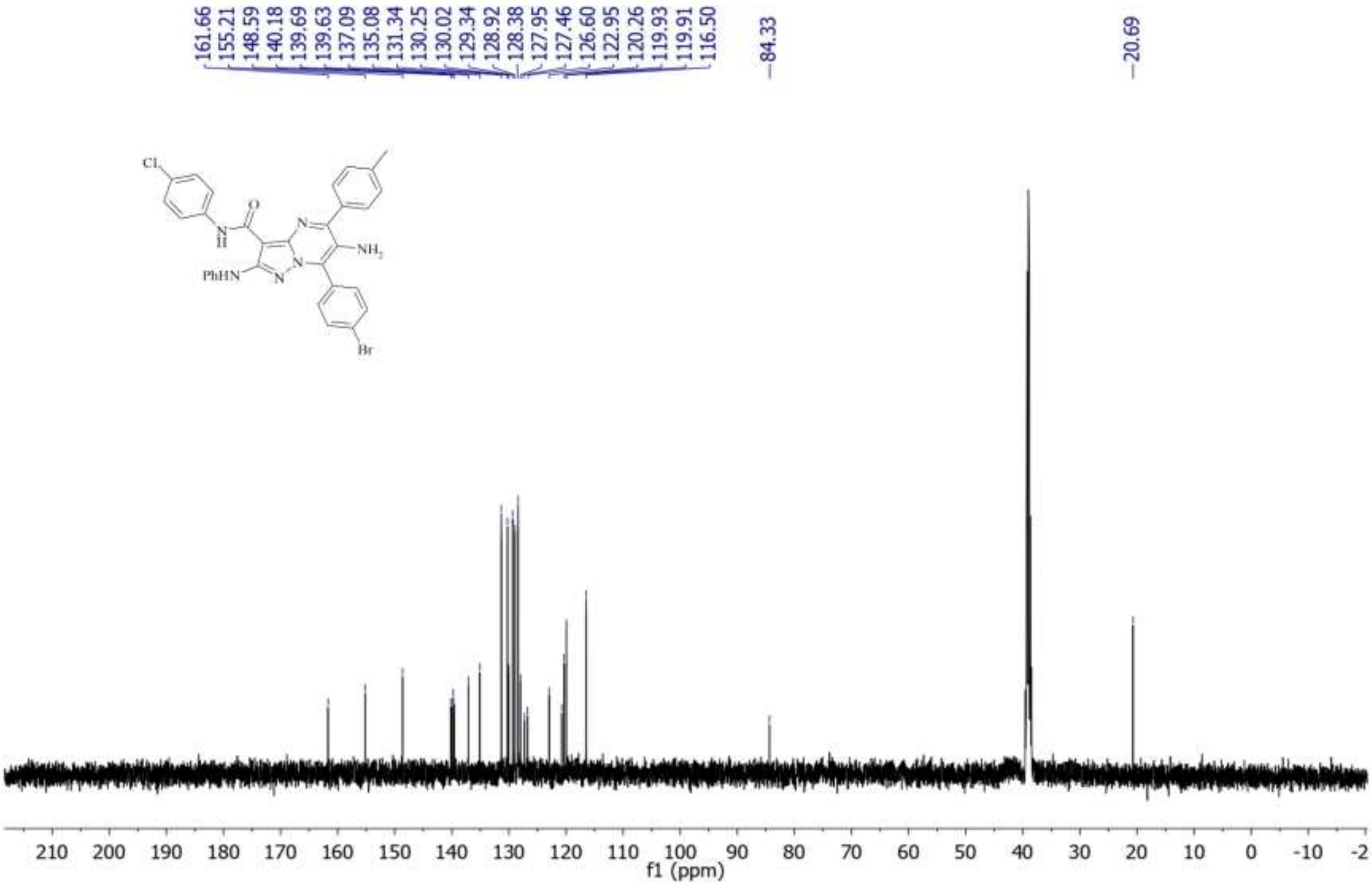

^{1}H NMR spectrum of 6-amino-N-(4-chlorophenyl)-5-(4-methoxyphenyl)-7-phenyl-2-(phenylamino)pyrazolo[1,5-a]pyrimidine-3-carboxamide (**3w**)

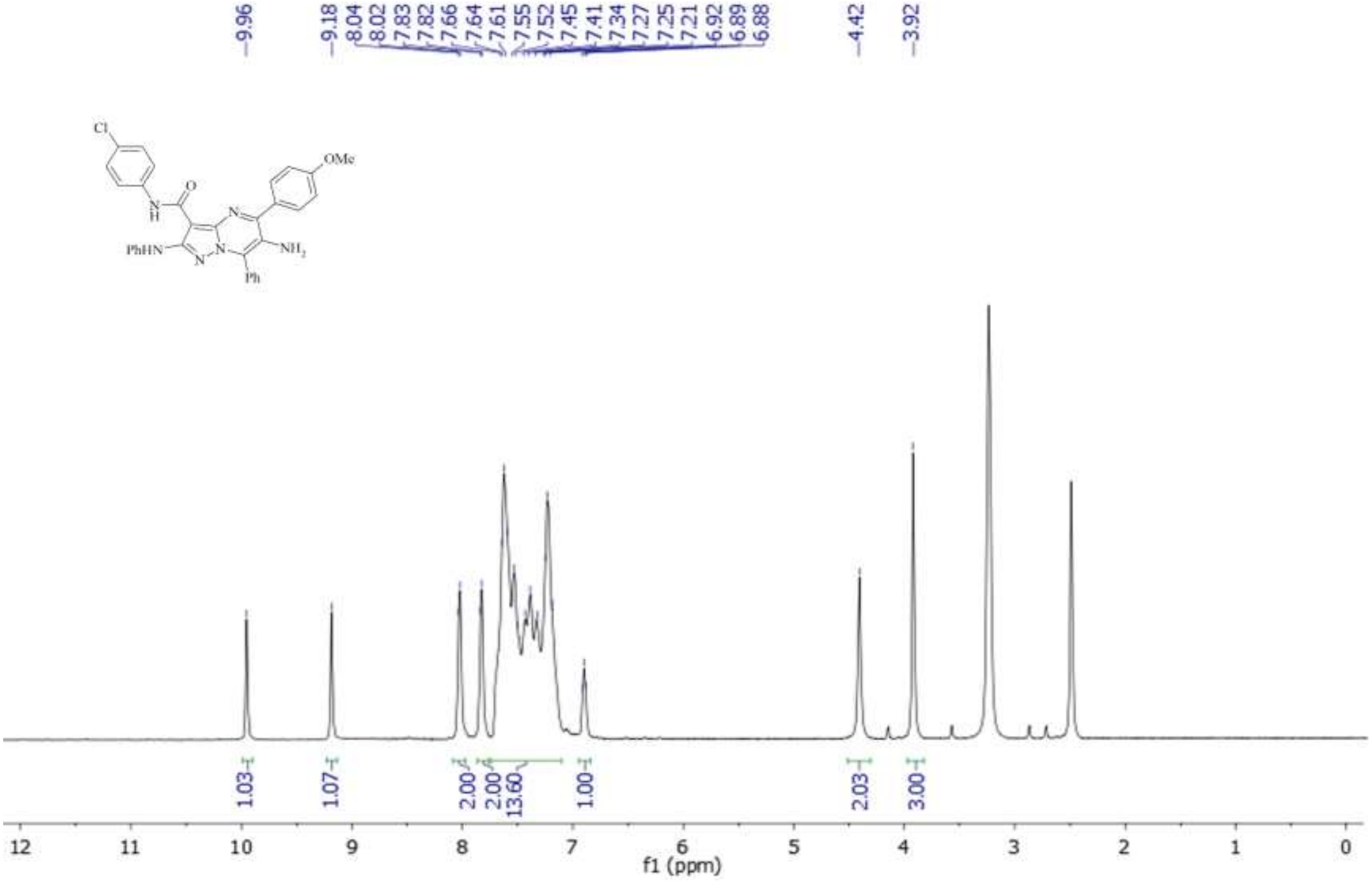

^{13}C NMR spectrum of 6-amino-N-(4-chlorophenyl)-5-(4-methoxyphenyl)-7-phenyl-2-(phenylamino)pyrazolo[1,5-a]pyrimidine-3-carboxamide (**3w**)

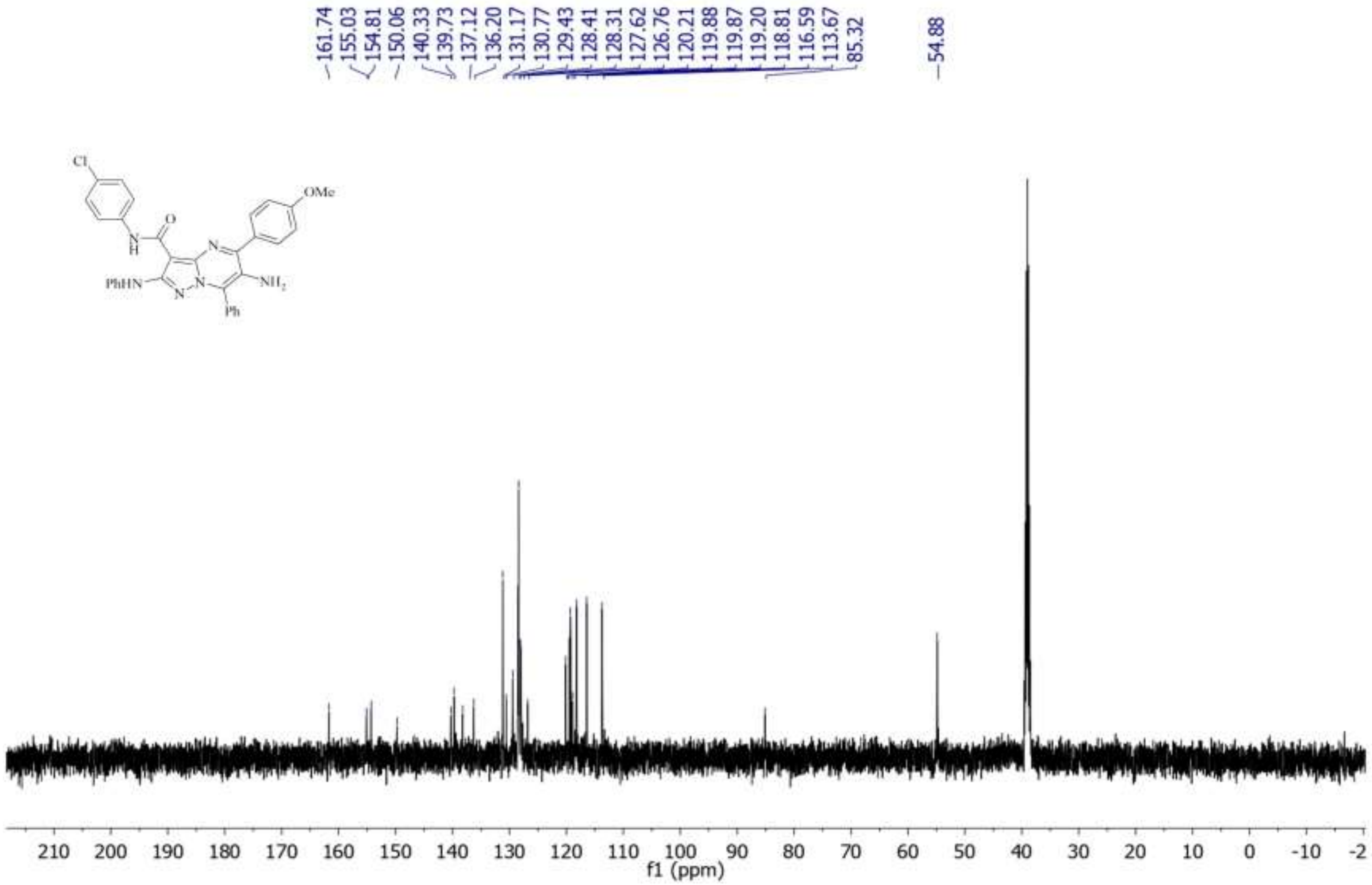

^{1}H NMR spectrum of 6-amino-N,5-bis(4-chlorophenyl)-7-phenyl-2-(phenylamino)pyrazolo[1,5-a]pyrimidine-3-carboxamide (**3x**)

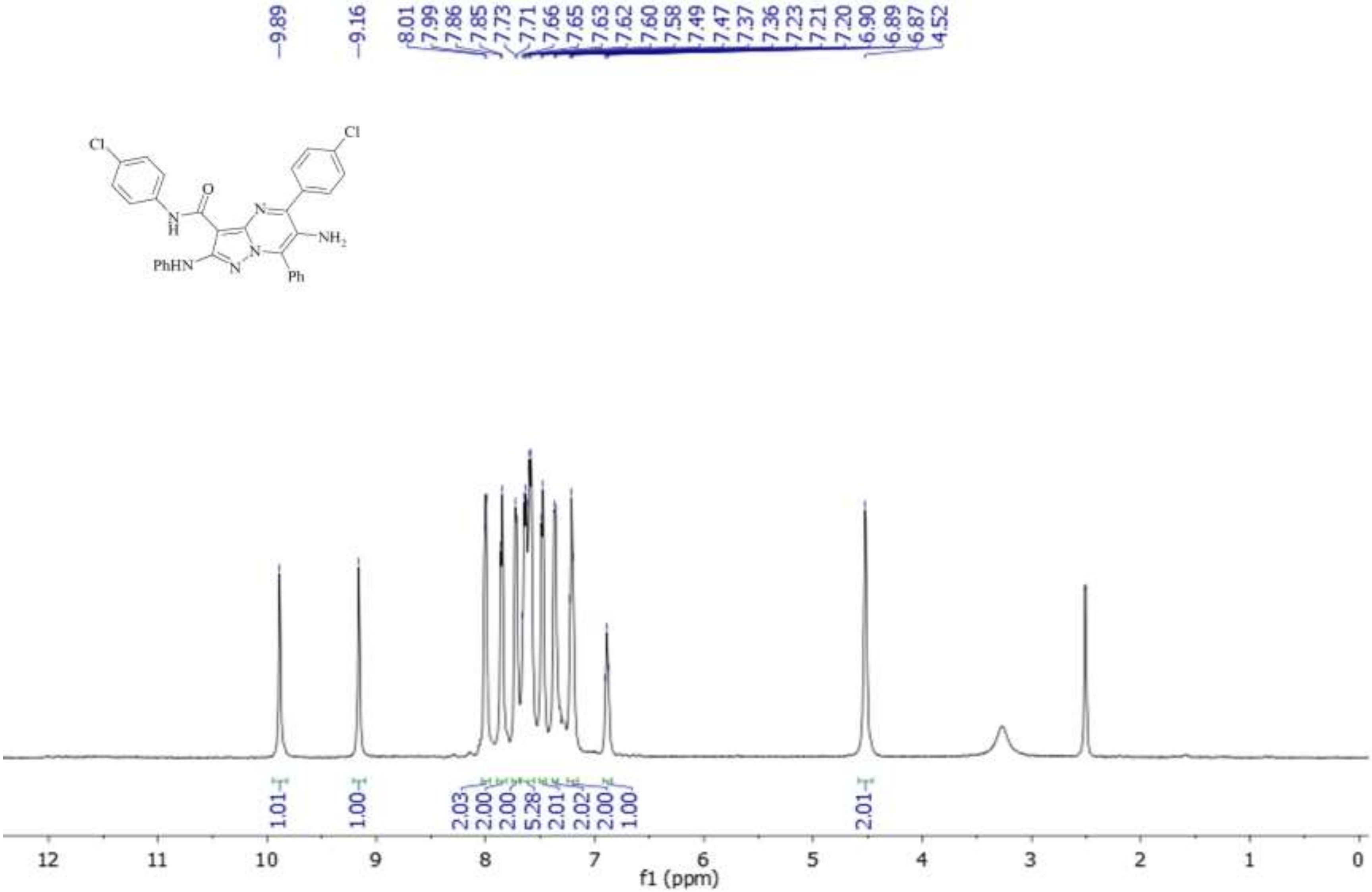

^{13}C NMR spectrum of 6-amino-N,5-bis(4-chlorophenyl)-7-phenyl-2-(phenylamino)pyrazolo[1,5-a]pyrimidine-3-carboxamide (**3x**)

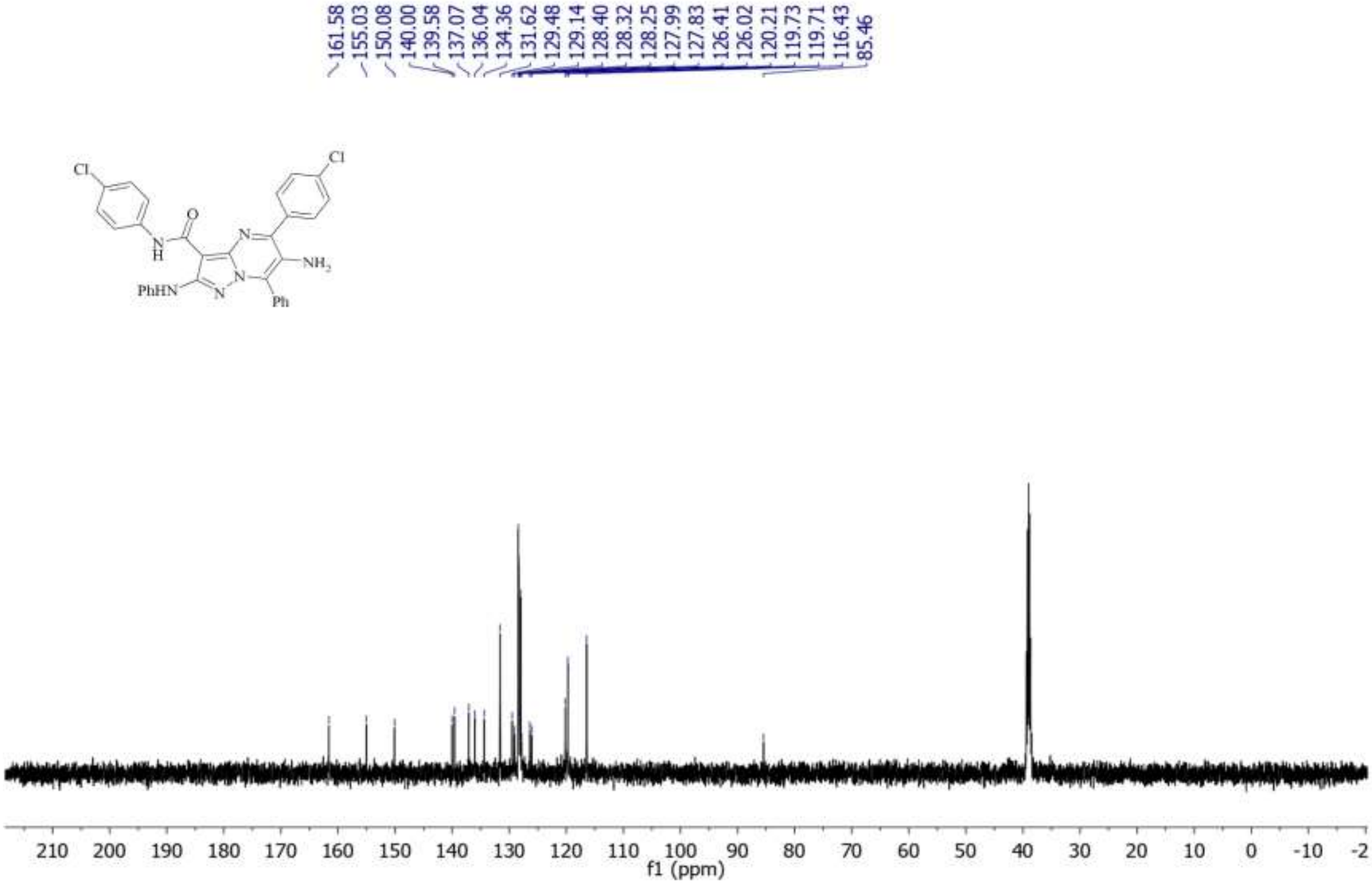

^{1}H NMR spectrum of 6-amino-N,5,7-tris(4-chlorophenyl)-2-(phenylamino)pyrazolo[1,5-a]pyrimidine-3-carboxamide (**3y**)

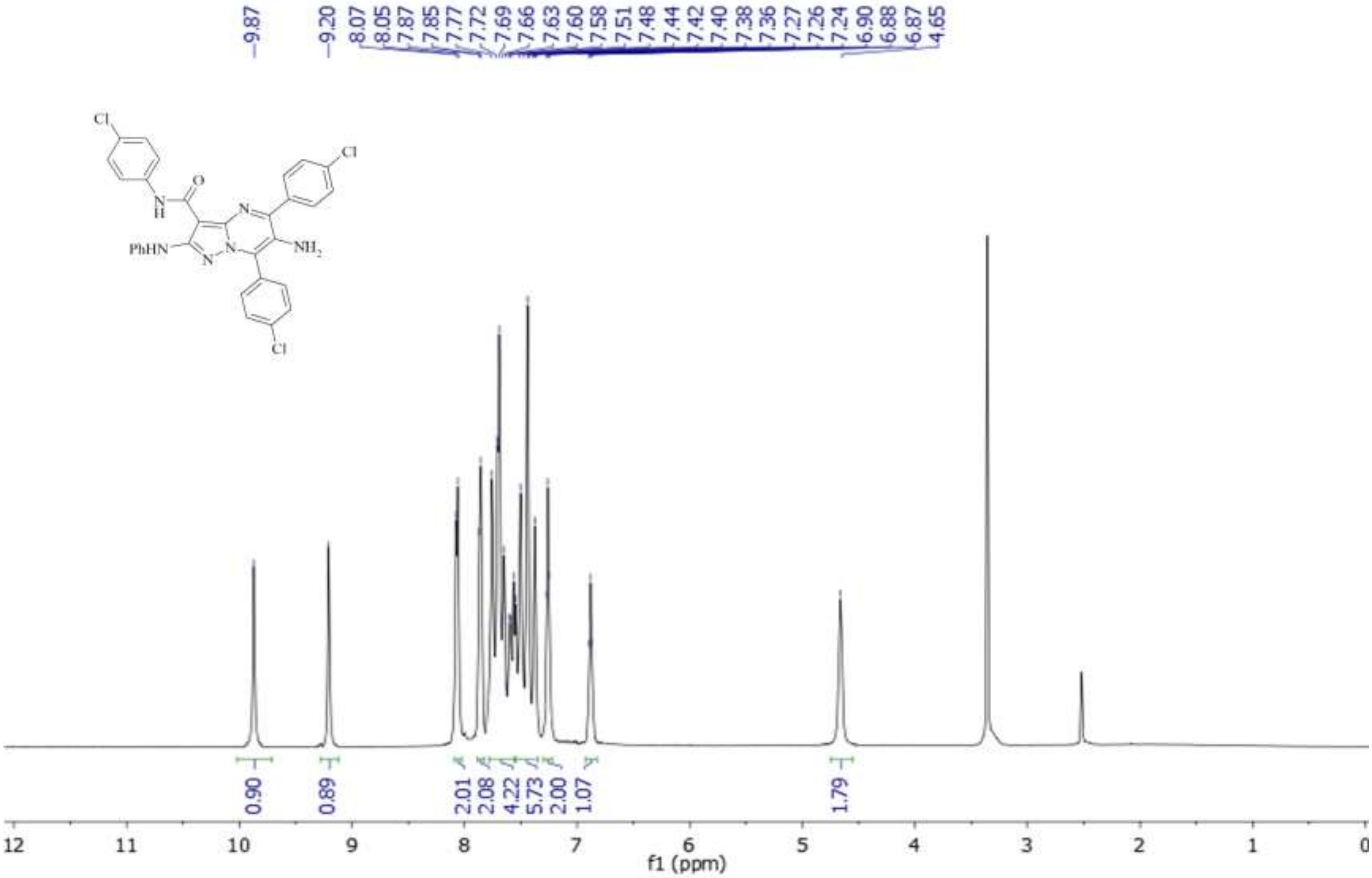

^{13}C NMR spectrum of 6-amino-N,5,7-tris(4-chlorophenyl)-2-(phenylamino)pyrazolo[1,5-a]pyrimidine-3-carboxamide (**3y**)

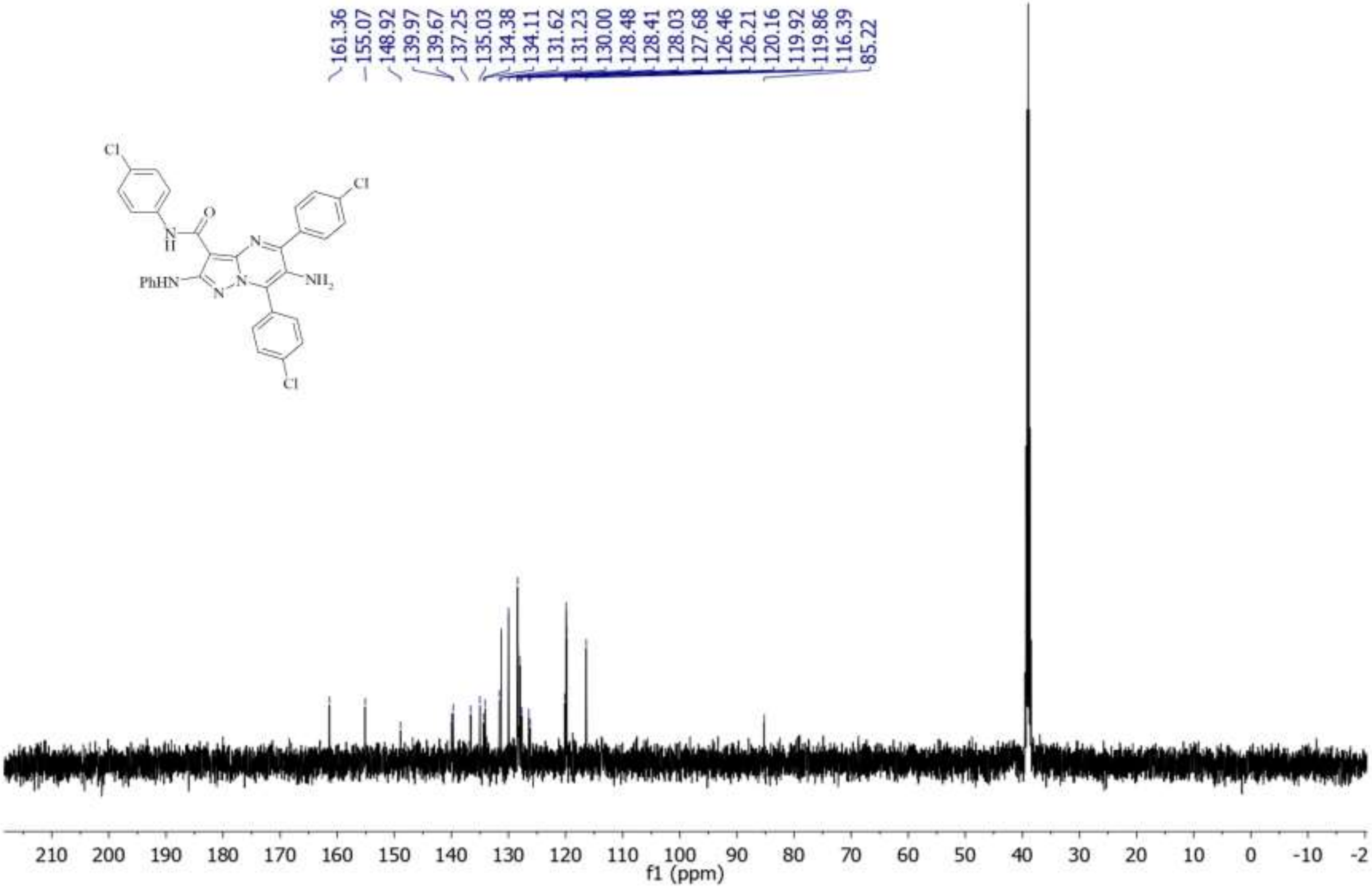

^{1}H NMR spectrum of 6-amino-N,5-bis(4-chlorophenyl)-2-(phenylamino)-7-(thiophen-2-yl)pyrazolo[1,5-a]pyrimidine-3-carboxamide (**3z**)

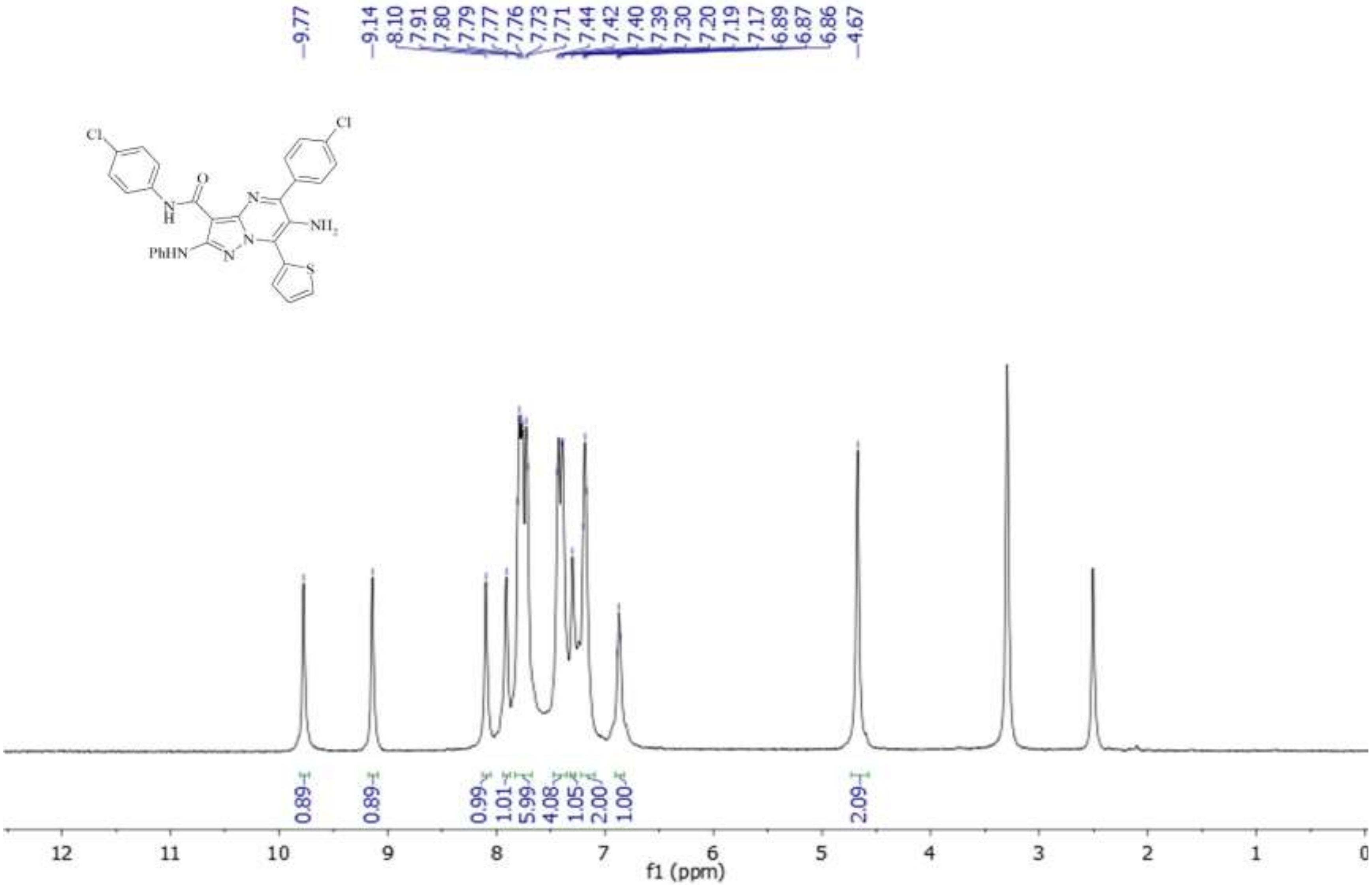

^{13}C NMR spectrum of 6-amino-N,5-bis(4-chlorophenyl)-2-(phenylamino)-7-(thiophen-2-yl)pyrazolo[1,5-a]pyrimidine-3-carboxamide (**3z**)

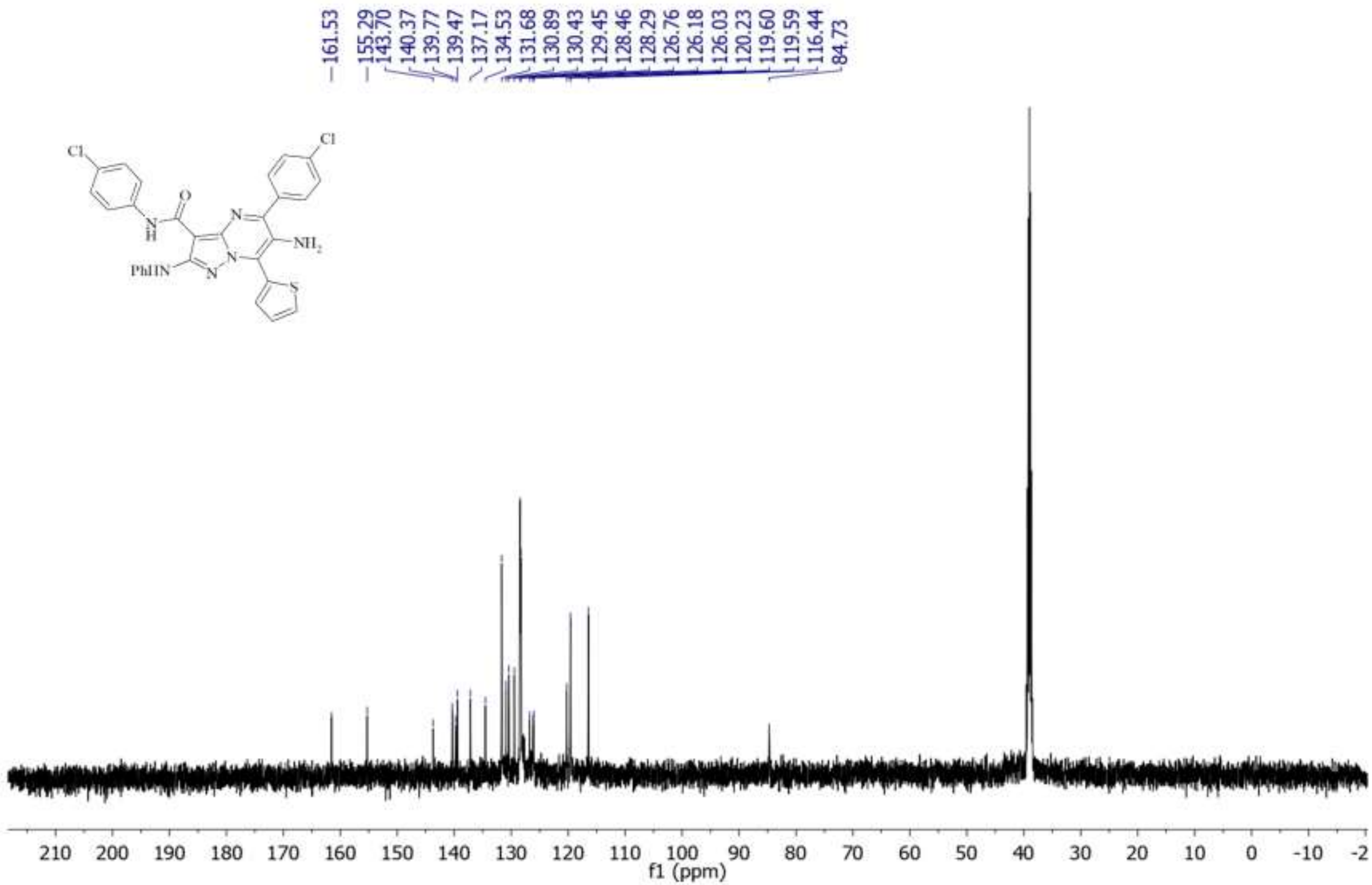

^{1}H NMR spectrum of Ethyl 6-amino-7-(4-chlorophenyl)-2,5-diphenylpyrazolo[1,5-a]pyrimidine-3-carboxylate (**3aa**)

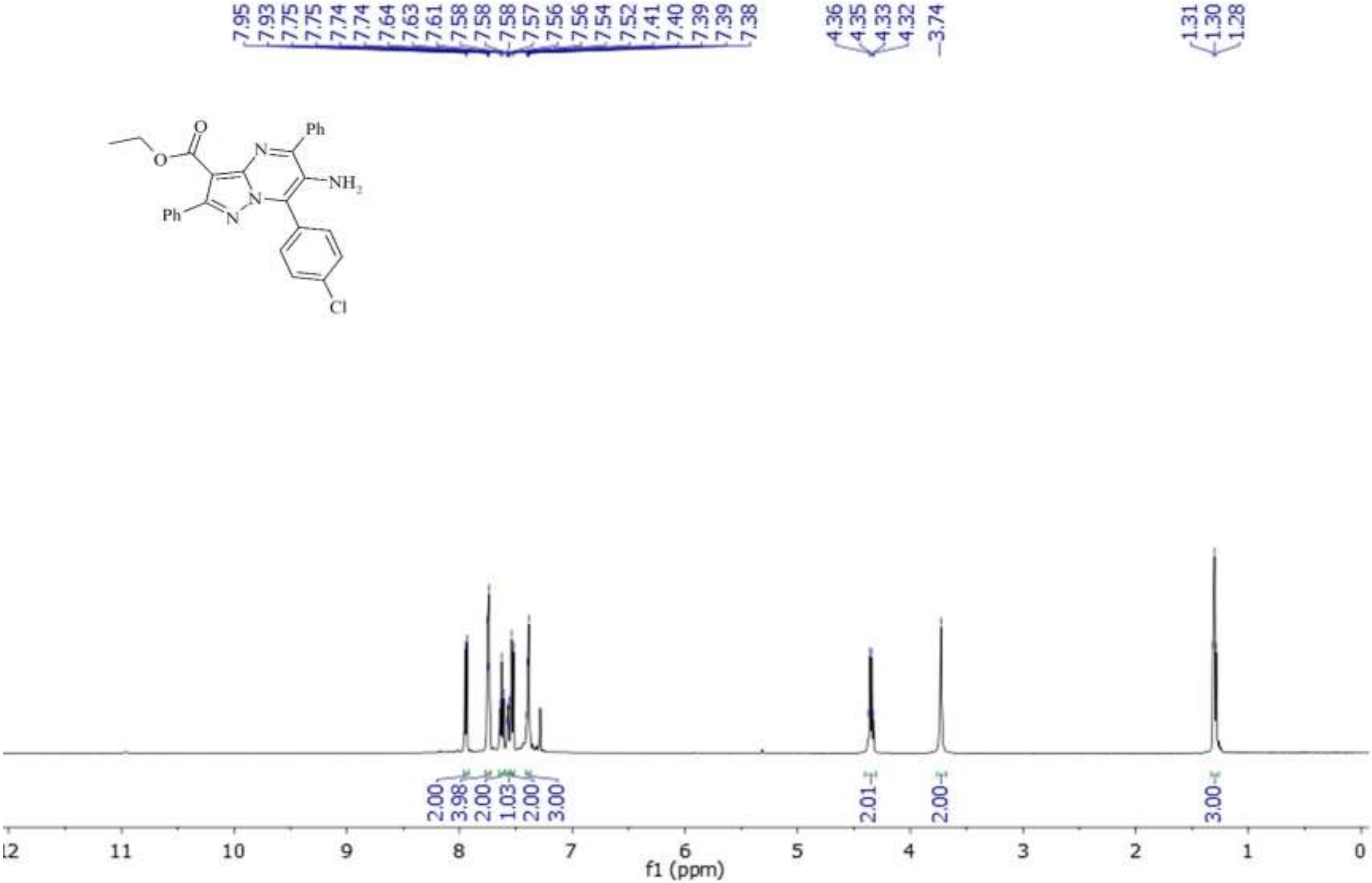

^{13}C NMR spectrum of Ethyl 6-amino-7-(4-chlorophenyl)-2,5-diphenylpyrazolo[1,5-a]pyrimidine-3-carboxylate (**3aa**)

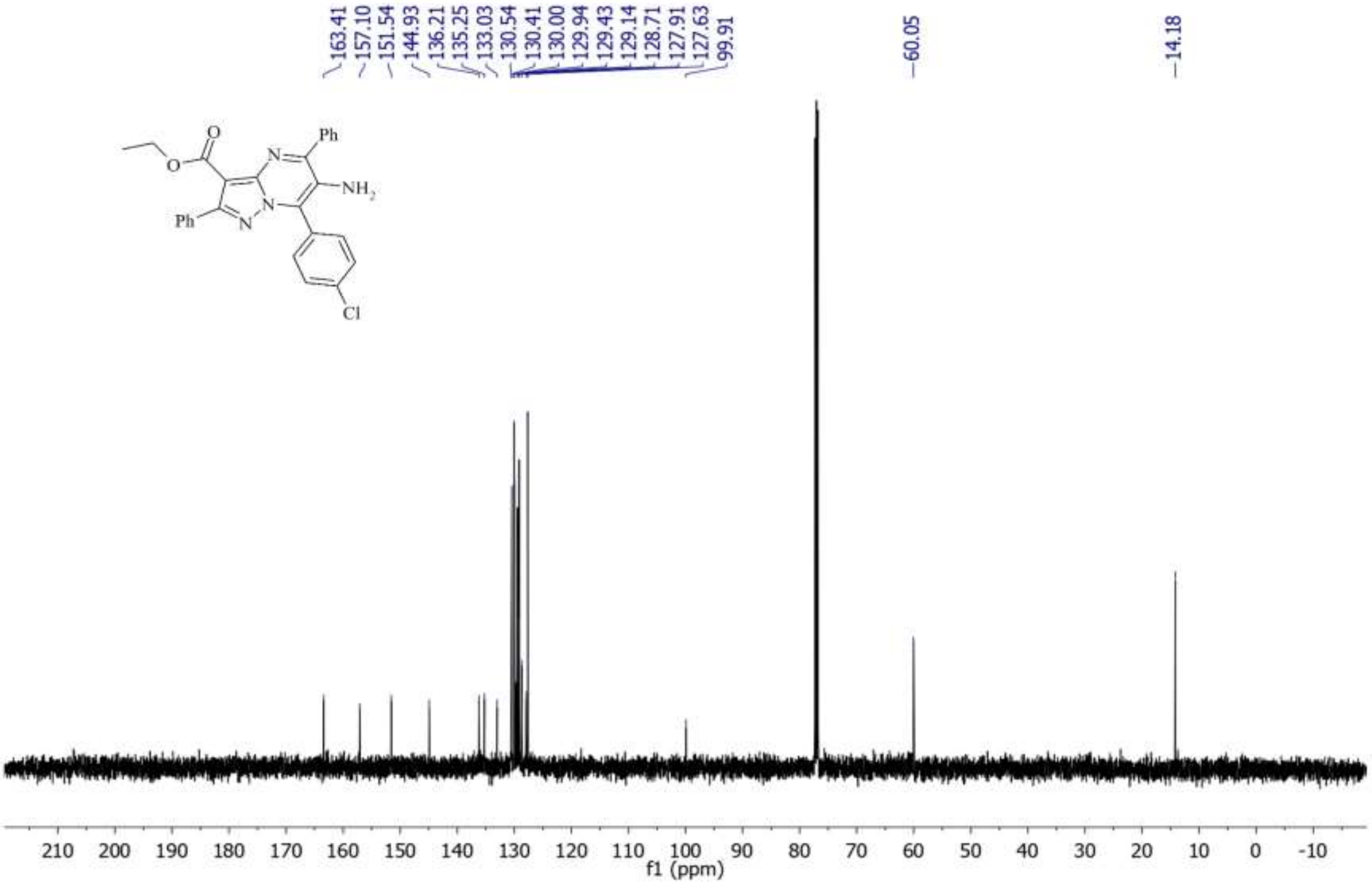

^{1}H NMR spectrum of ethyl 6-amino-7-(4-bromophenyl)-2,5-diphenylpyrazolo[1,5-a]pyrimidine-3-carboxylate (**3ab**)

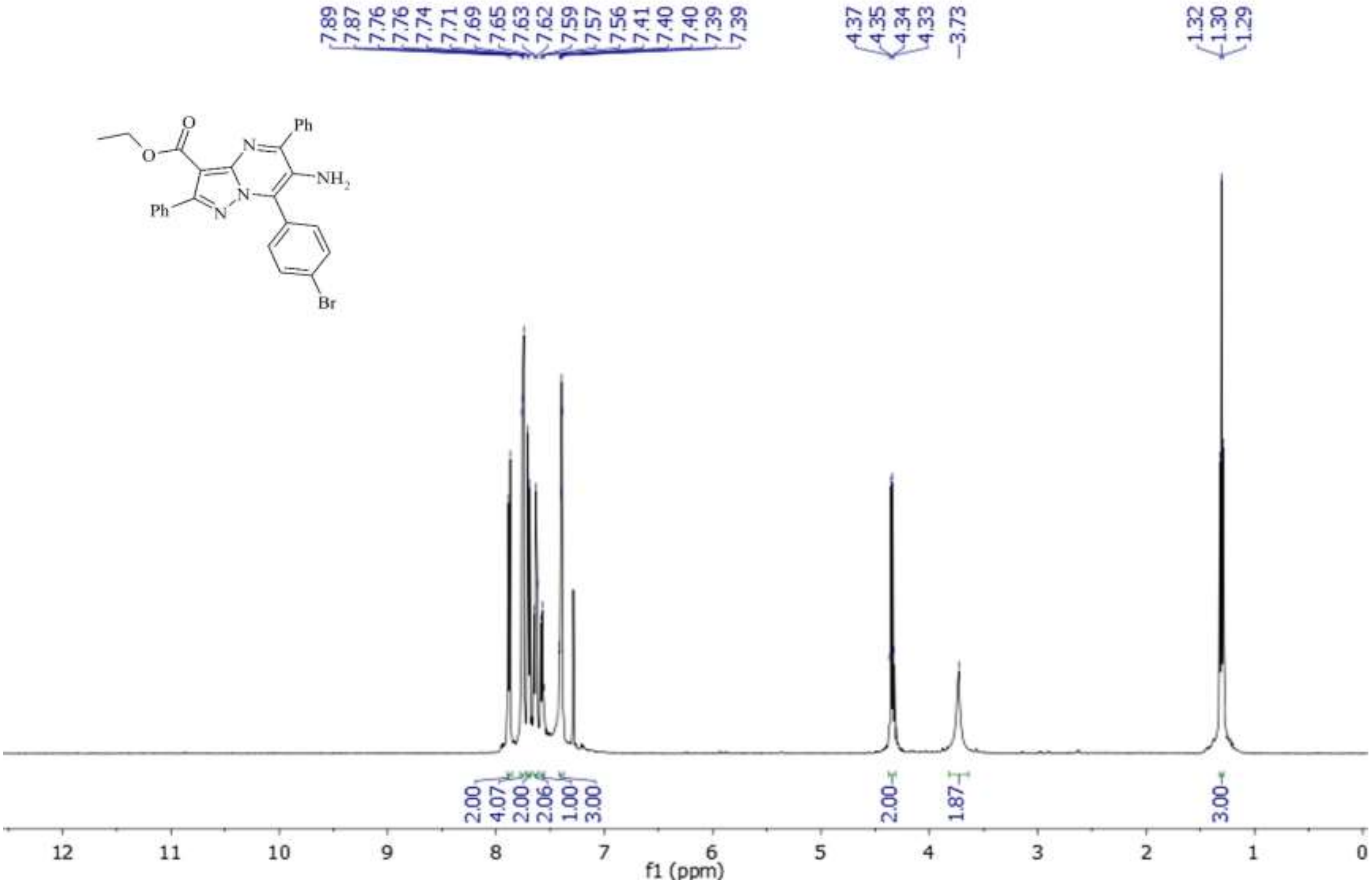

^{13}C NMR spectrum of ethyl 6-amino-7-(4-bromophenyl)-2,5-diphenylpyrazolo[1,5-a]pyrimidine-3-carboxylate (**3ab**)

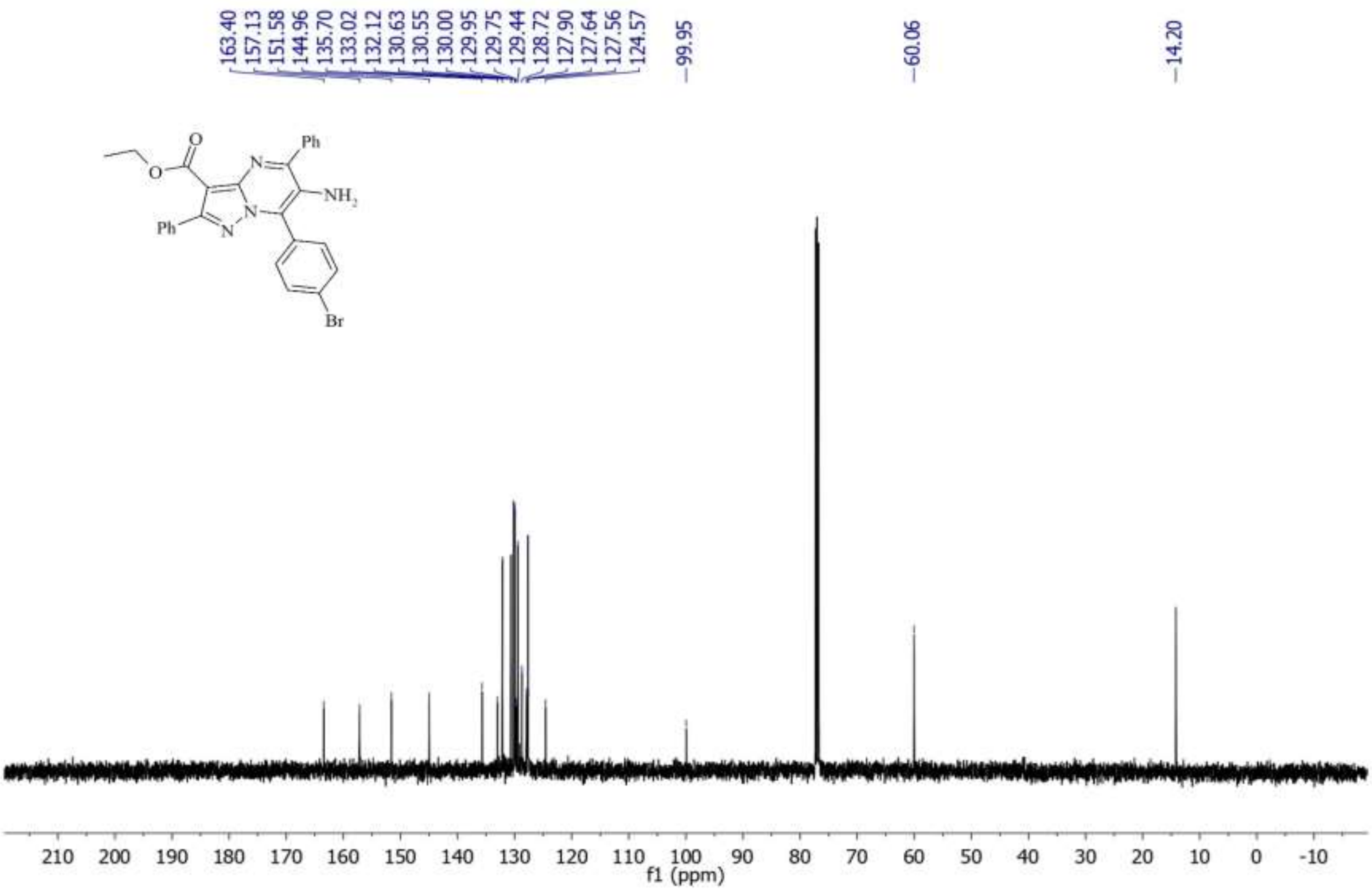

^{1}H NMR spectrum ethyl 6-amino-7-(4-chlorophenyl)-2-phenyl-5-p-tolylpyrazolo[1,5-a]pyrimidine-3-carboxylate (**3ac**)

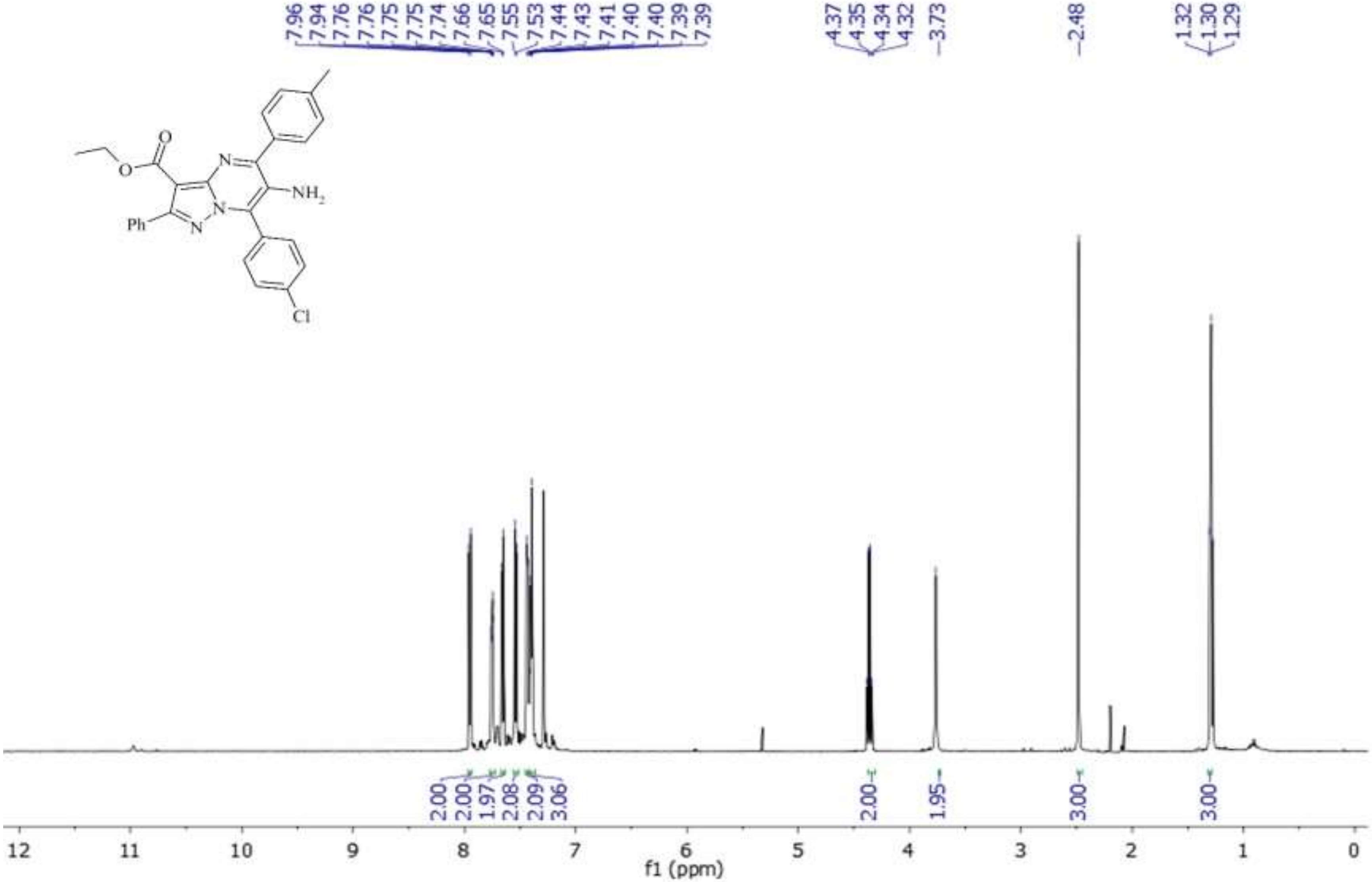

^{13}C NMR spectrum ethyl 6-amino-7-(4-chlorophenyl)-2-phenyl-5-p-tolylpyrazolo[1,5-a]pyrimidine-3-carboxylate (**3ac**)

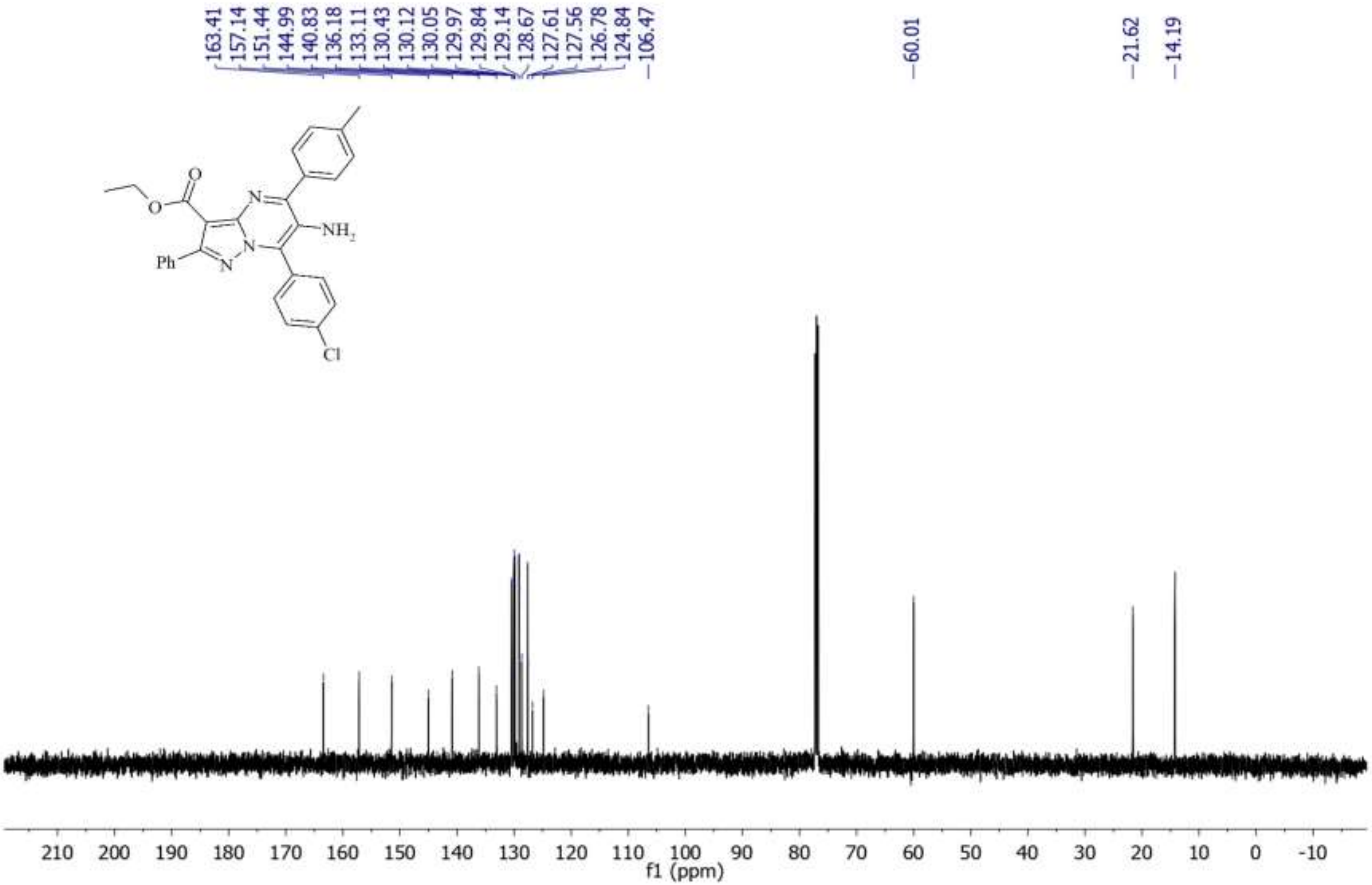

^{1}H NMR spectrum of ethyl 6-amino-7-(4-bromophenyl)-2-phenyl-5-p-tolylpyrazolo[1,5-a]pyrimidine-3-carboxylate (**3ad**)

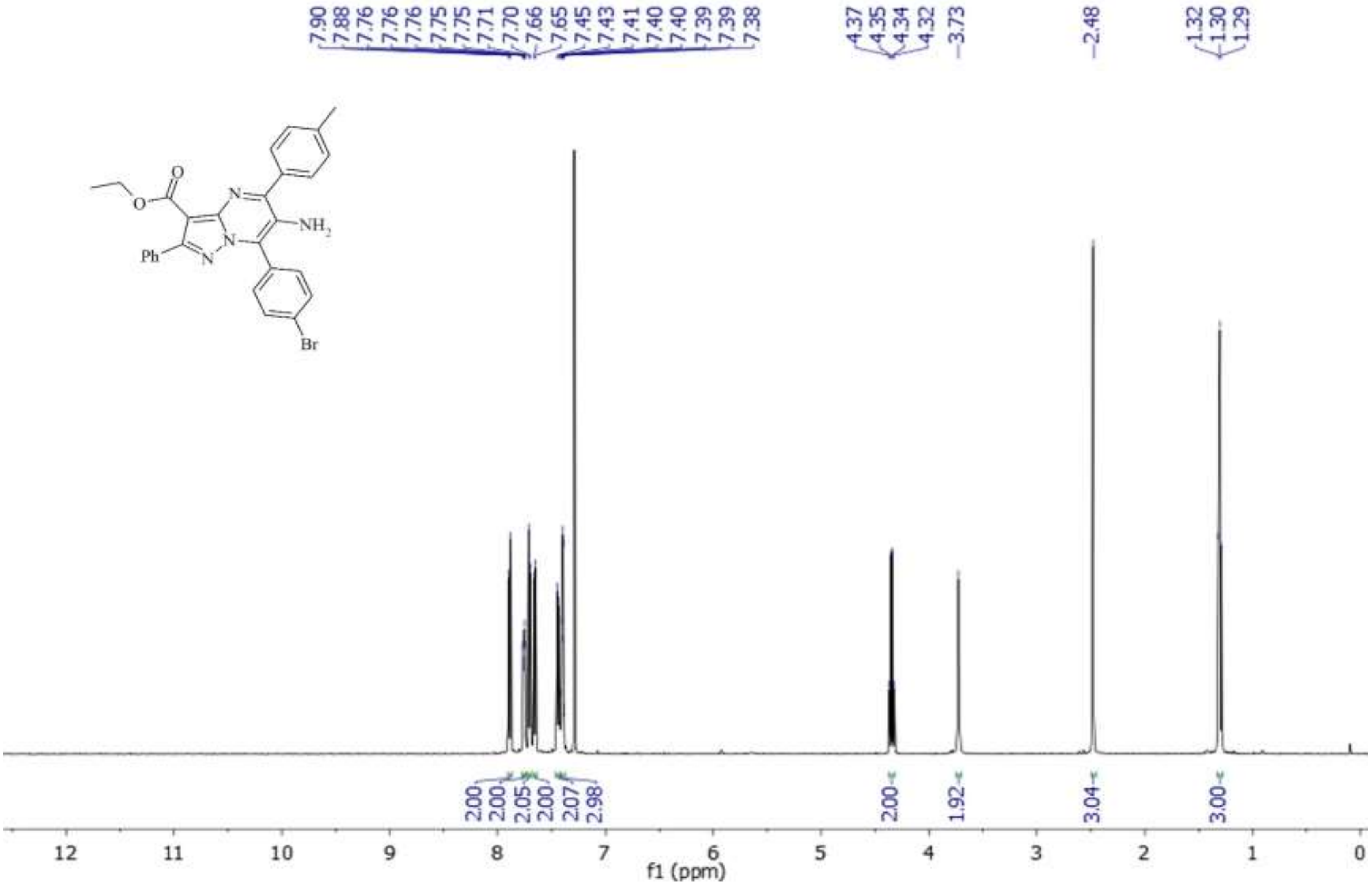

^{13}C NMR spectrum of ethyl 6-amino-7-(4-bromophenyl)-2-phenyl-5-p-tolylpyrazolo[1,5-a]pyrimidine-3-carboxylate (**3ad**)

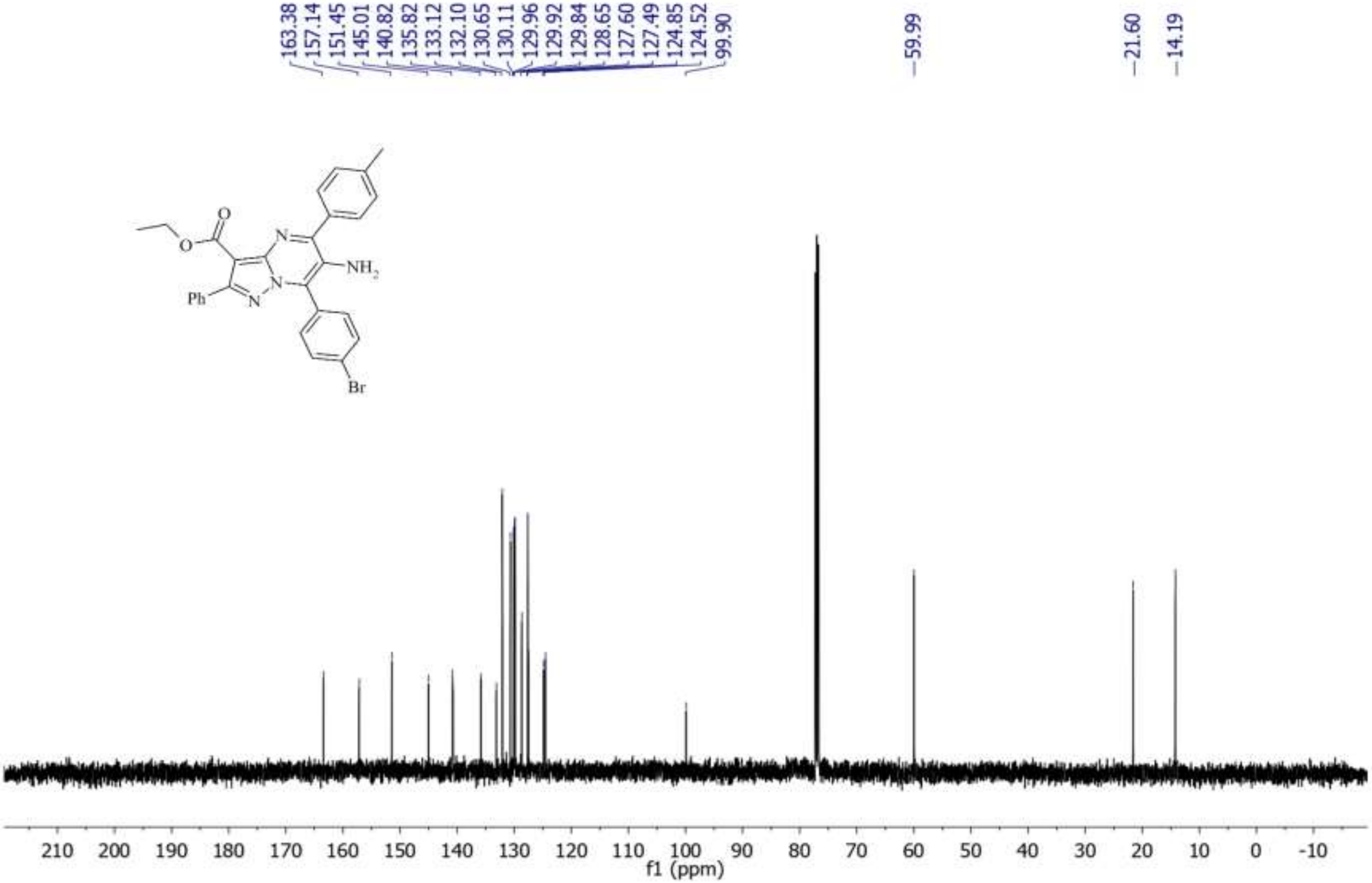

[1] H NMR spectrum of ethyl 6-amino-5-(4-methoxyphenyl)-2,7-diphenylpyrazolo[1,5-a]pyrimidine-3-carboxylate (**3ae**)

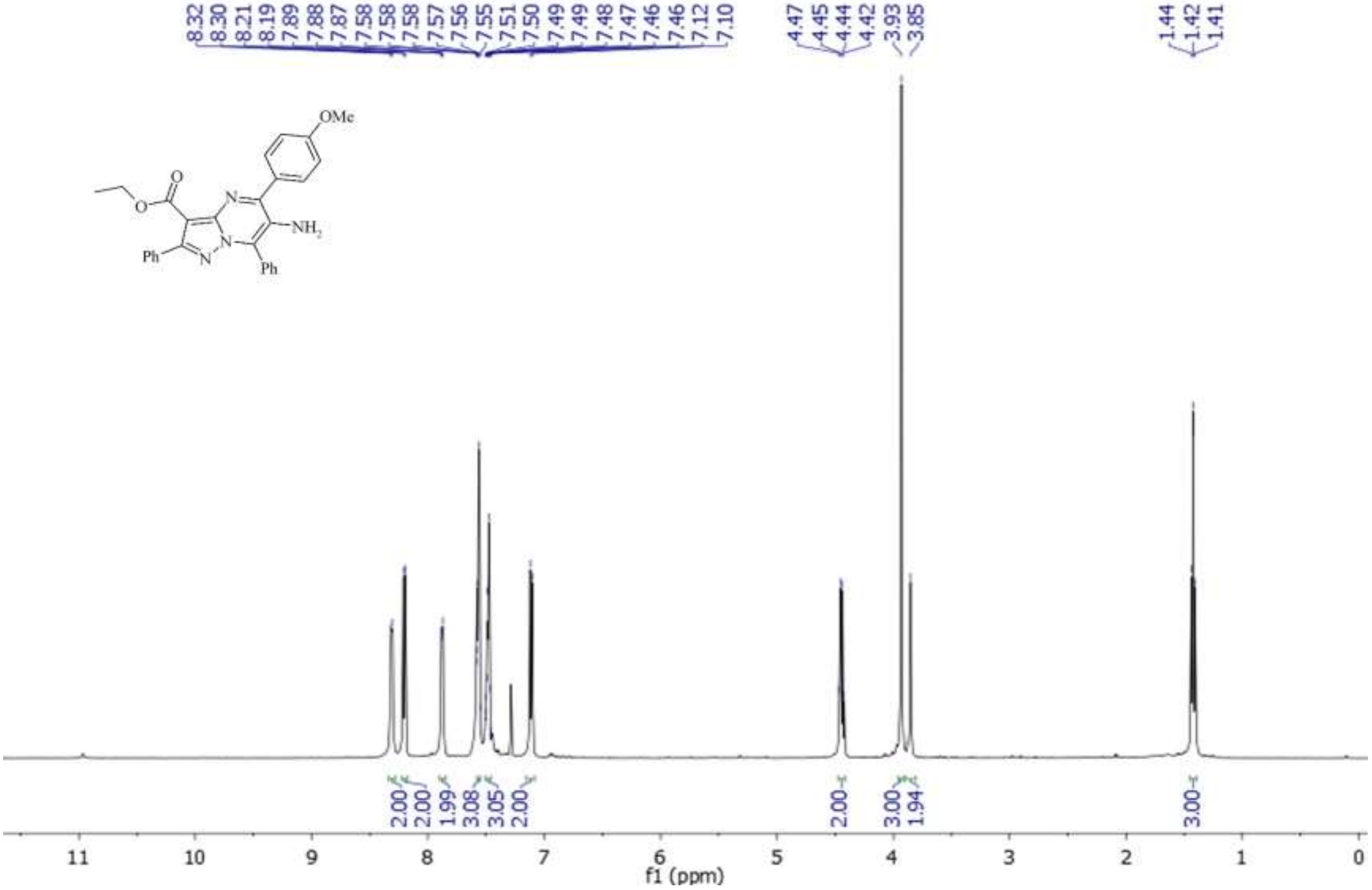

^{13}C NMR spectrum of ethyl 6-amino-5-(4-methoxyphenyl)-2,7-diphenylpyrazolo[1,5-a]pyrimidine-3-carboxylate (**3ae**)

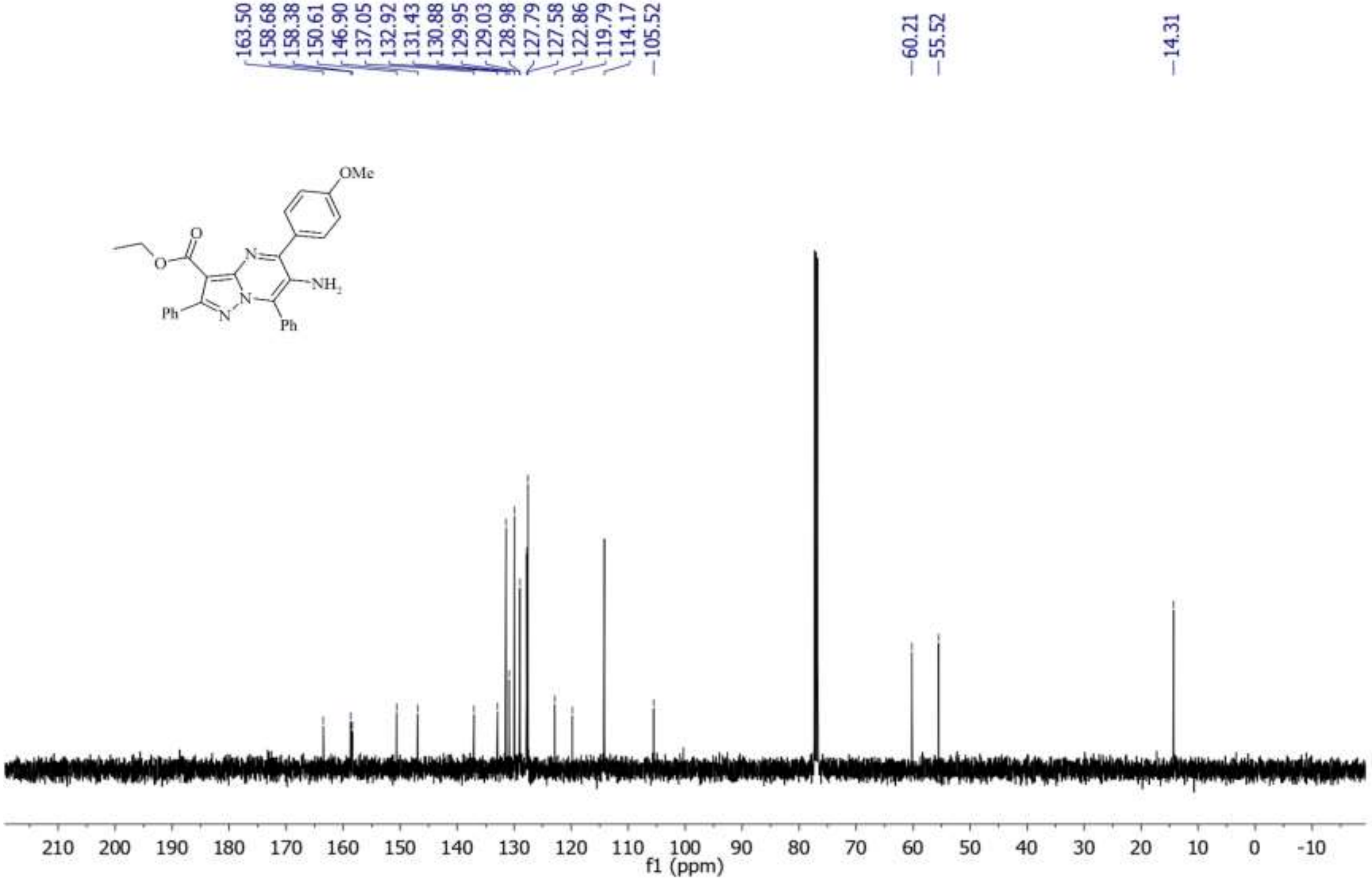

^{1}H NMR spectrum of ethyl 6-amino-7-(4-chlorophenyl)-5-(4-methoxyphenyl)-2-phenylpyrazolo[1,5-a]pyrimidine-3-carboxylate (**3af**)

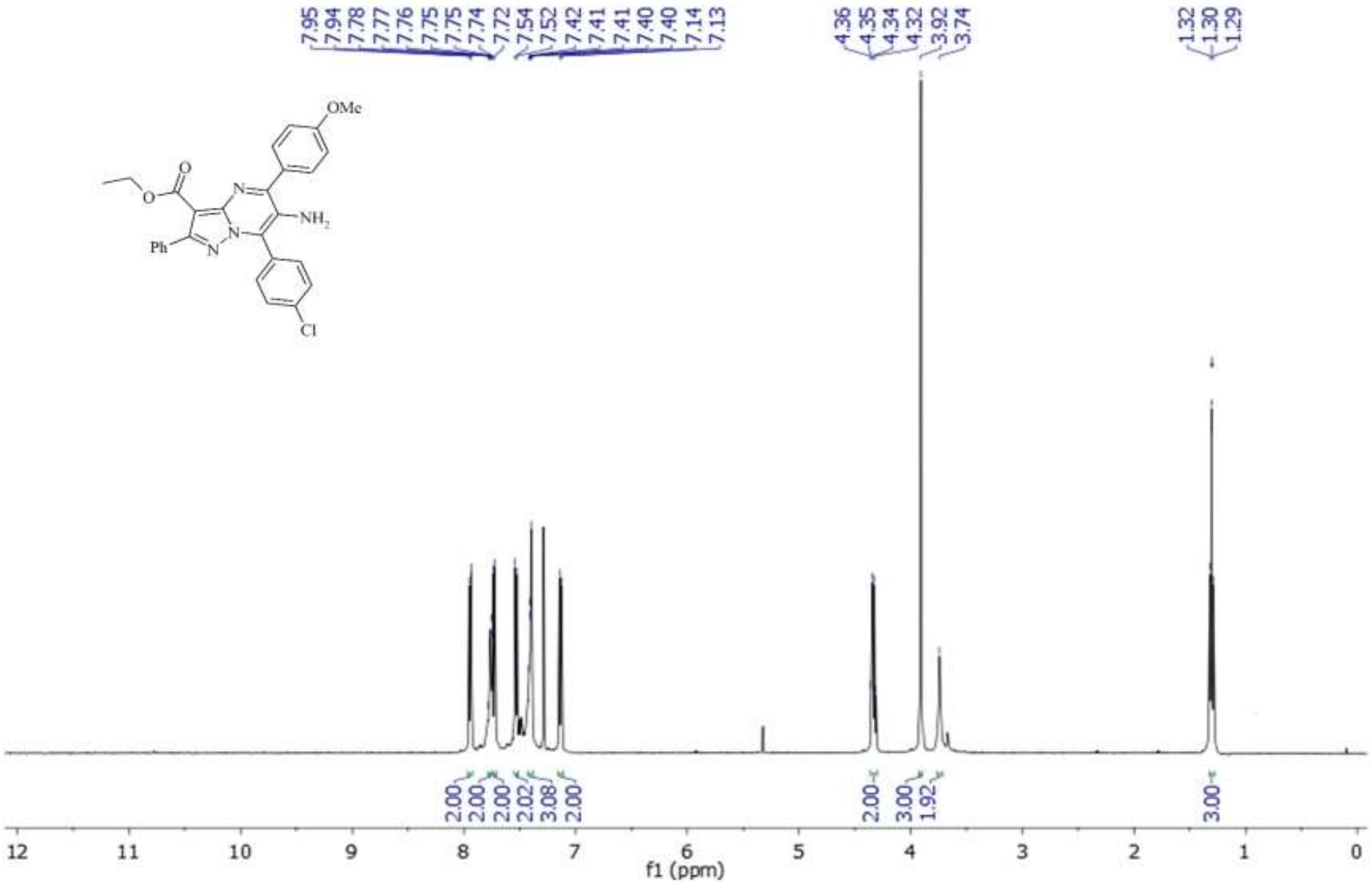

^{13}C NMR spectrum of ethyl 6-amino-7-(4-chlorophenyl)-5-(4-methoxyphenyl)-2-phenylpyrazolo[1,5-a]pyrimidine-3-carboxylate (**3af**)

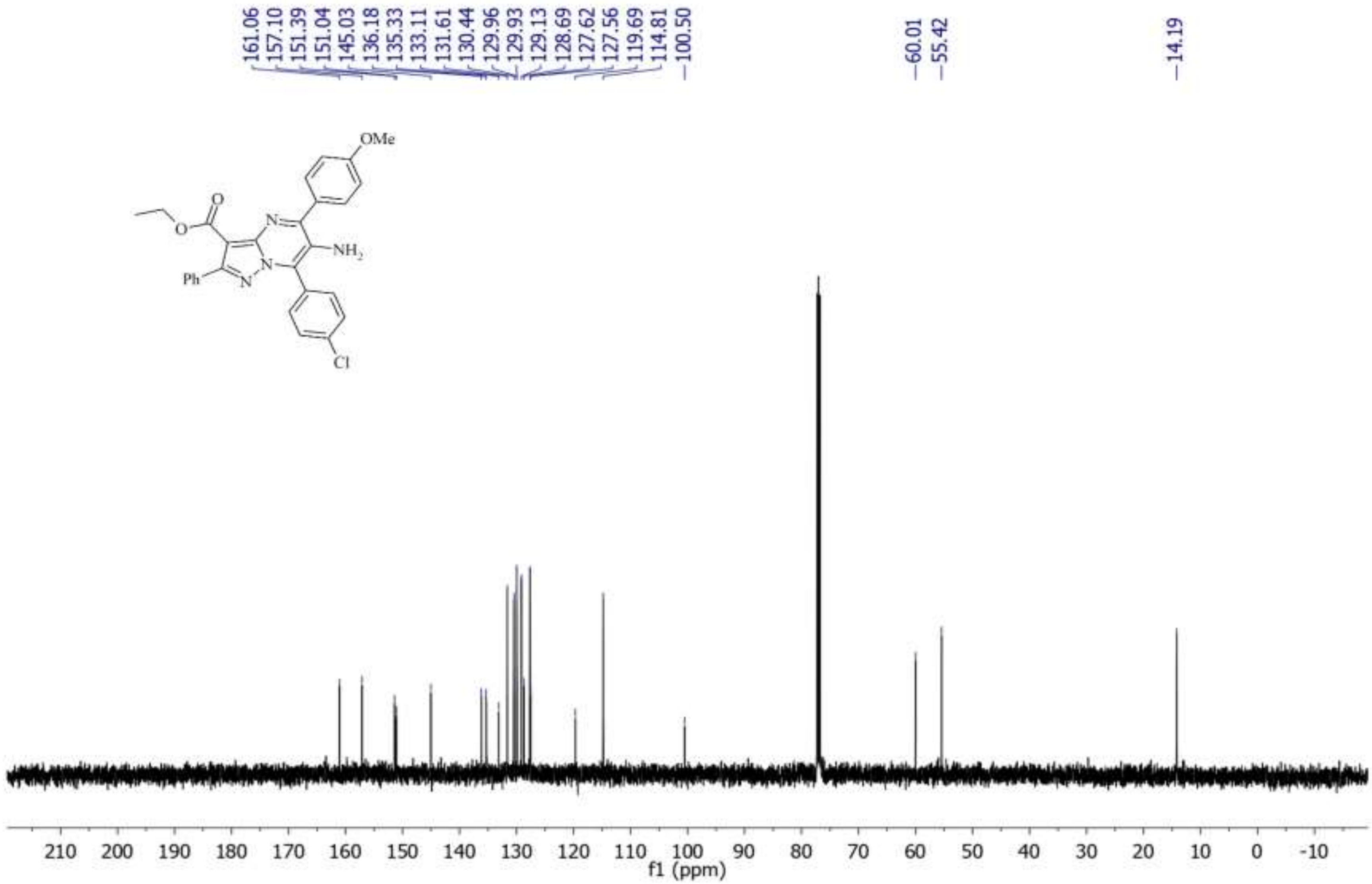

^{1}H NMR spectrum of ethyl 6-amino-5-(4-chlorophenyl)-2,7-diphenylpyrazolo[1,5-a]pyrimidine-3-carboxylate (**3ag**)

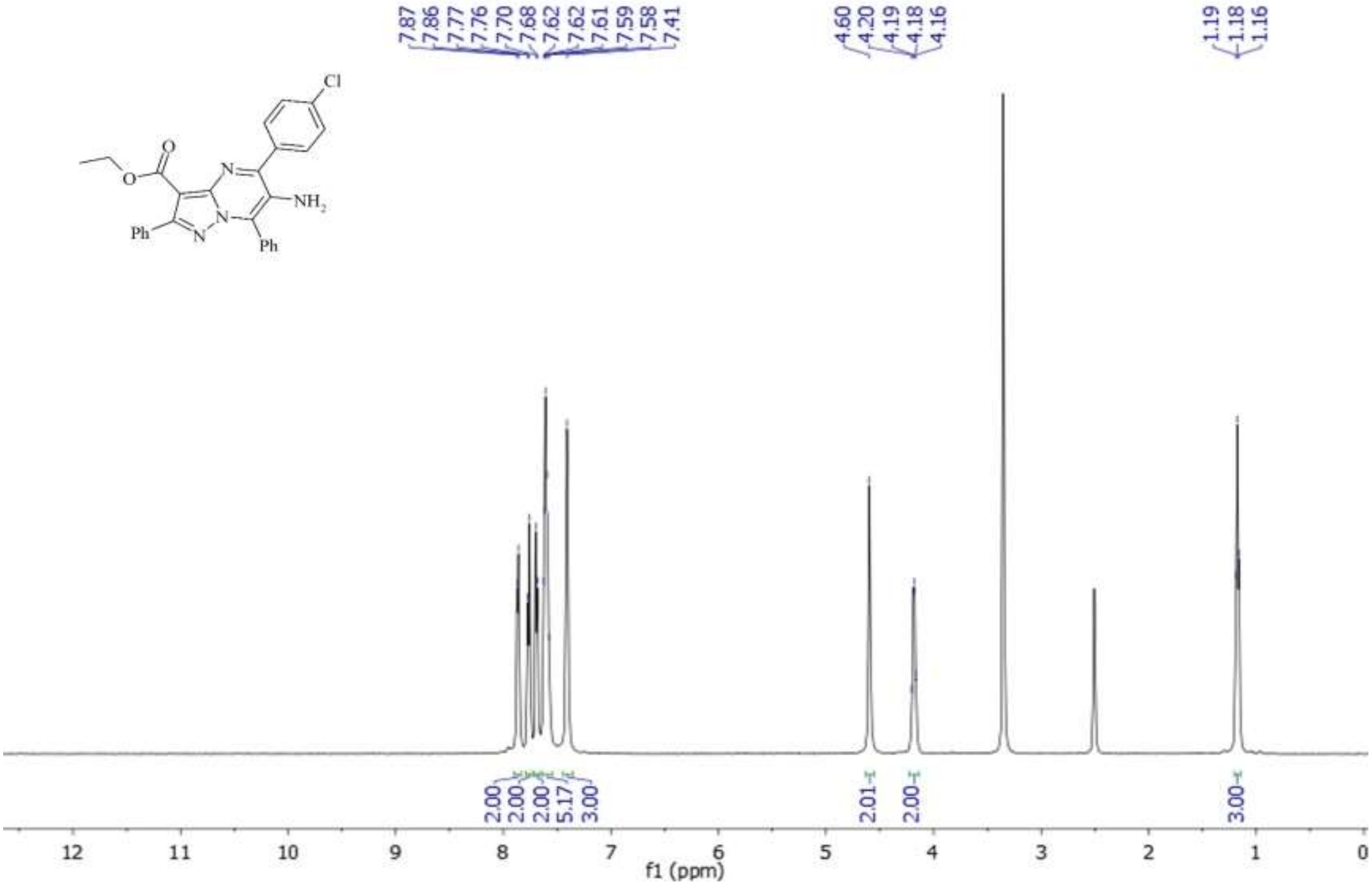

^{13}C NMR spectrum of ethyl 6-amino-5-(4-chlorophenyl)-2,7-diphenylpyrazolo[1,5-a]pyrimidine-3-carboxylate (**3ag**)

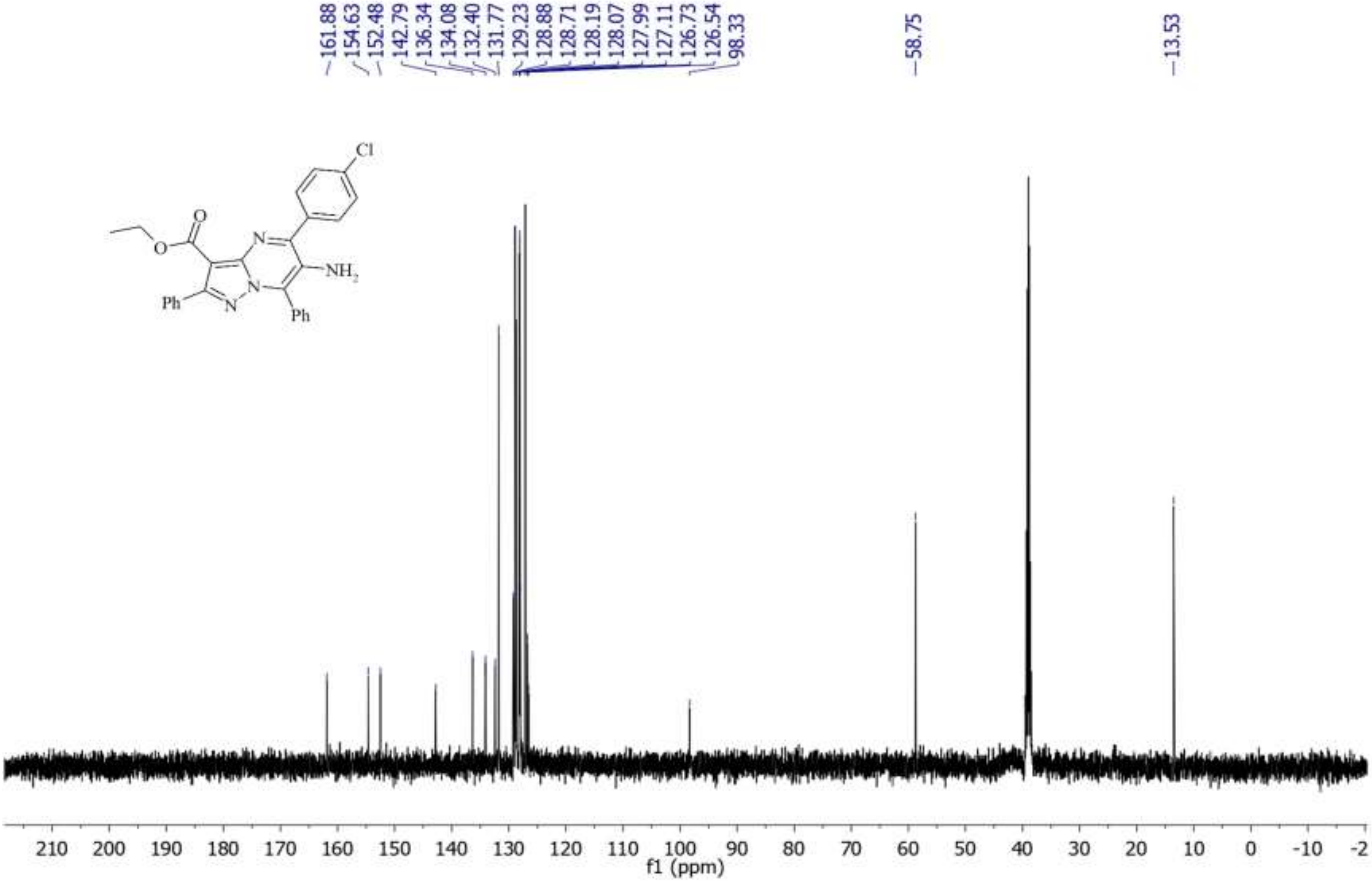

^{1}H NMR spectrum of ethyl 6-amino-5,7-bis(4-chlorophenyl)-2-phenylpyrazolo[1,5-a]pyrimidine-3-carboxylate (**3ah**)

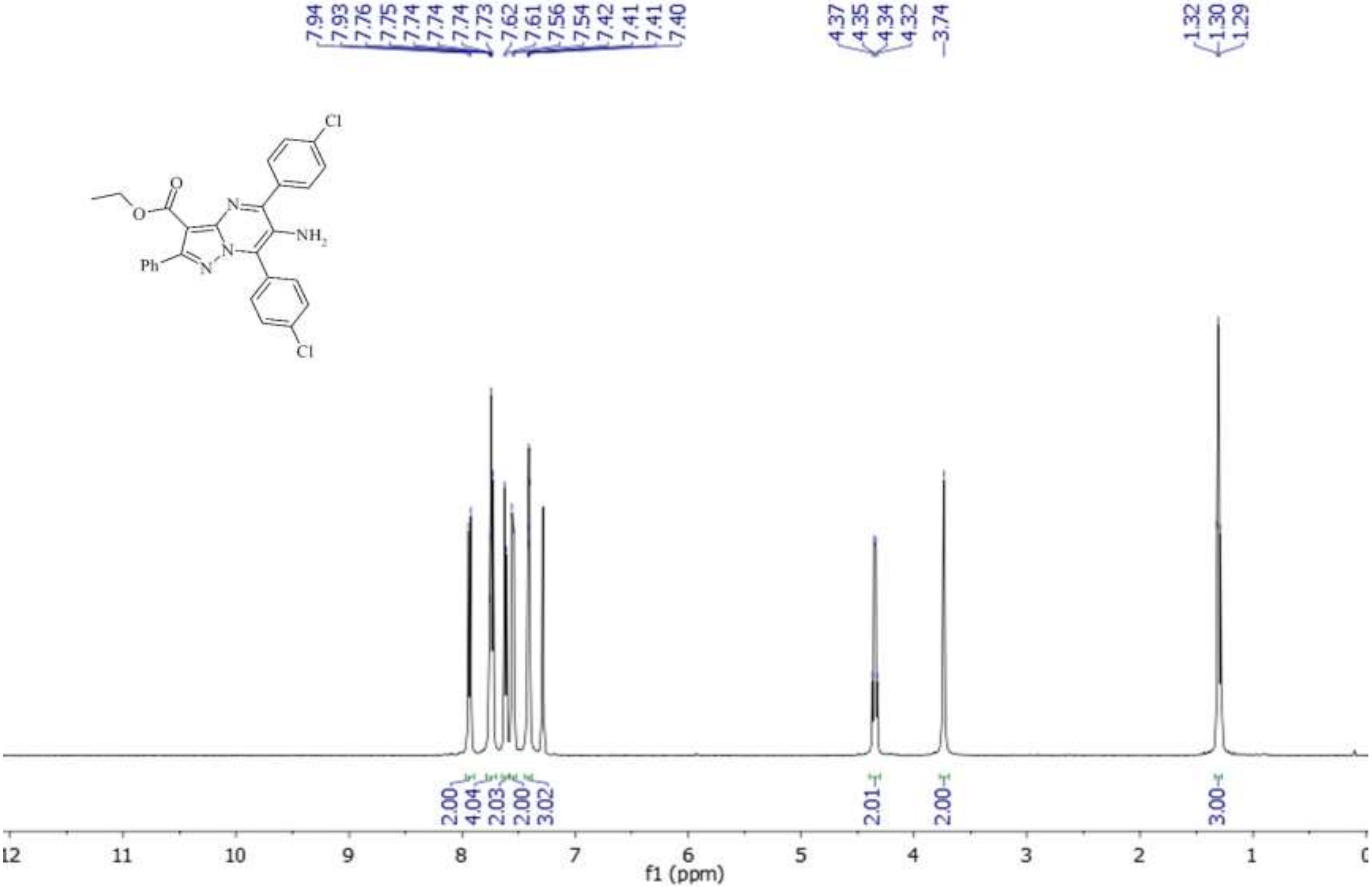

^{13}C NMR spectrum of ethyl 6-amino-5,7-bis(4-chlorophenyl)-2-phenylpyrazolo[1,5-a]pyrimidine-3-carboxylate (**3ah**)

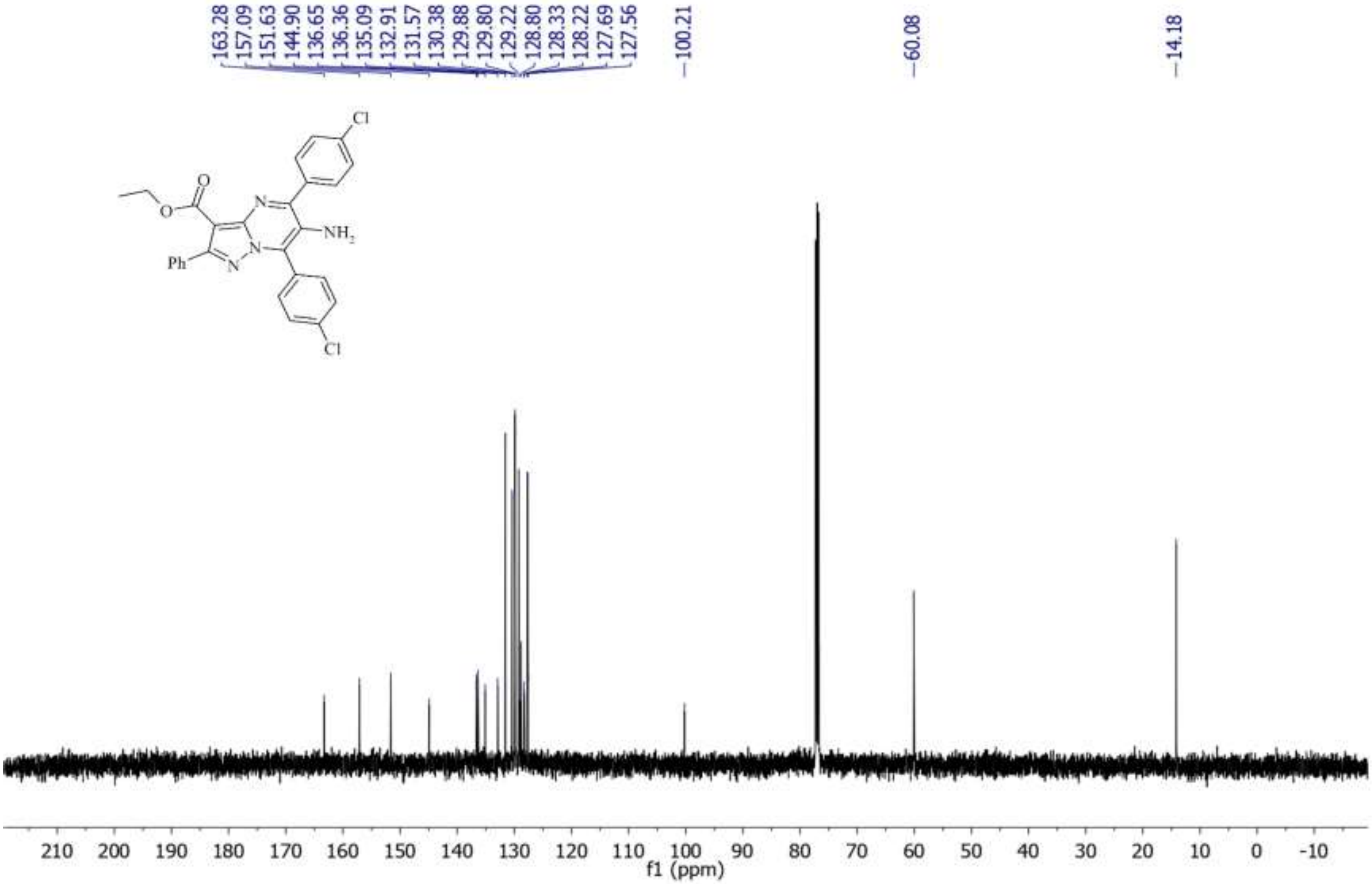

^{1}H NMR spectrum of ethyl 6-amino-5-(4-chlorophenyl)-2-phenyl-7-(thiophen-2-yl)pyrazolo[1,5-a]pyrimidine-3-carboxylate (**3ai**)

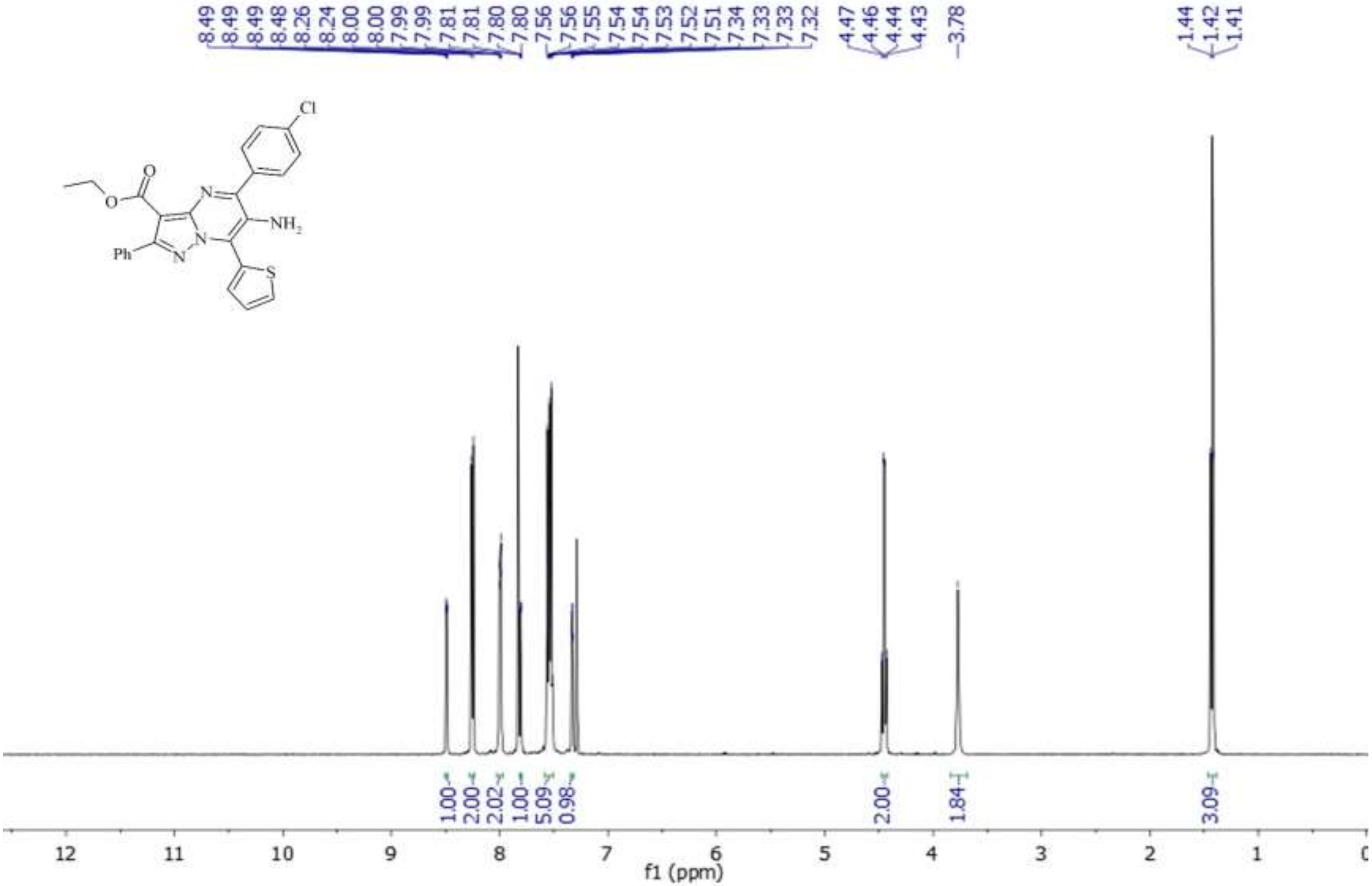

^{13}C NMR spectrum of ethyl 6-amino-5-(4-chlorophenyl)-2-phenyl-7-(thiophen-2-yl)pyrazolo[1,5-a]pyrimidine-3-carboxylate (**3ai**)

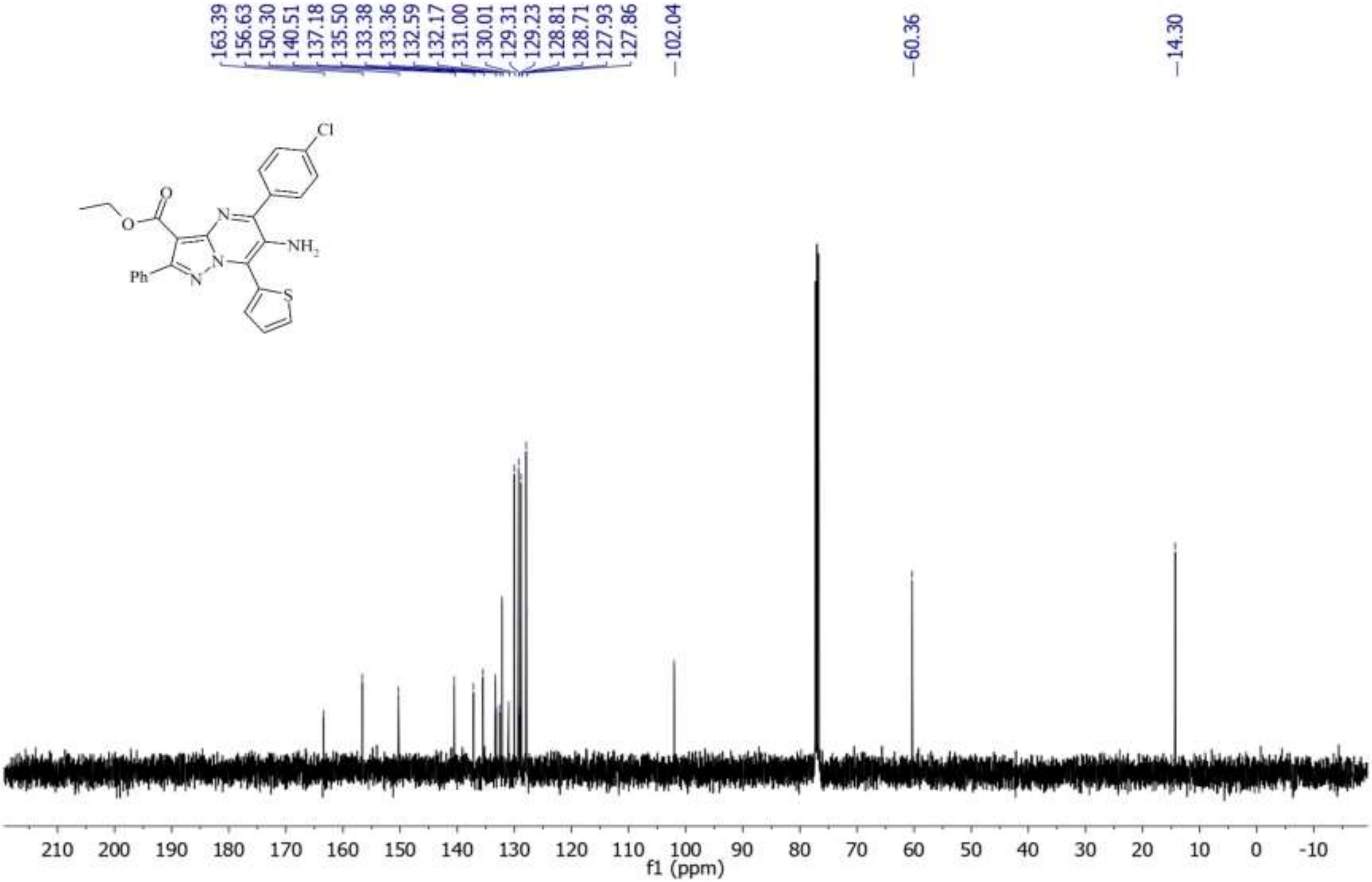

Supplement: Supplementary file 1 — Supplememtary information. [file 41598_2020_59079_MOESM1_ESM.pdf]
